# Payoff selectivity drives collective intelligence during dynamic resource tracking in humans

**DOI:** 10.1038/s41467-026-77762-z

**Published:** 2026-09-23

**Authors:** Valerii Chirkov, Ralf H.J.M. Kurvers, Pawel Romanczuk, Dominik Deffner

**Affiliations:** 1 Center for Adaptive Rationality, Max Planck Institute for Human Development, Berlin, Germany; 2 Science of Intelligence, Research Cluster of Excellence, Berlin, Germany; 3 Institute for Theoretical Biology, Department of Biology, Humboldt Universität zu Berlin, Berlin, Germany; 4 Department of Psychology, Philipps-University Marburg, Marburg, Germany

**Keywords:** Human behaviour, Computational biology and bioinformatics, Behavioural ecology

## Abstract

Humans’ abilities for complex social learning and collective adaptation stand out across species. Yet it is largely unknown how people integrate dynamically changing personal and social cues in realistic environments, resulting in collective intelligence or maladaptive behavior. Here, we report an online 3D immersive-reality experiment in which participants (*n* = 621) search for and track a mobile resource under realistic visual–spatial constraints. Participants complete the task either alone or in groups of five with different types of social information and resource speeds. Contrasting earlier work, participants in groups outperform solitary individuals, but only when payoff information was available. High-resolution visual field data and movement trajectories reveal that payoff visibility prompts adaptive recalibration of visual information flow, thereby enabling flexible adaptation. Computational models of fine-grained movement decisions and agent-based simulations show that payoff information lets participants dynamically identify and selectively respond to reliable social cues, thus unlocking the group’s collective potential.

## Abstract (web summary)

Humans rely on complex social learning and collective adaptation to navigate dynamically changing environments. The authors show that human collective intelligence in a dynamic, naturalistic task depends on access to payoff information.

## Introduction

Humans are uniquely flexible decision-makers, dynamically combining personal and social sources of information^1–4^. Social information can inform adaptive behavior and facilitate collective adaptation to fluctuating environments^5,6^, but not all social information is equally useful, and overreliance on others can result in maladaptive herding and collective breakdown^7–9^. Moving beyond cataloging social learning strategies^1,10^, recent studies have investigated the flexible integration of different social and asocial cues, linking individual-level strategies to collective adaptations^3,7,9^. Emerging theoretical frameworks suggest that the same cognitive mechanisms might underlie learning about both social and non-social environmental features, with individuals assessing social cues as indicators of potential rewards^11,12^.

The cues individuals rely on in realistic collective scenarios and how they arbitrate between dynamically changing social and non-social features to achieve collective intelligence remains poorly understood. For example, how do social foragers searching for fish or mushrooms integrate past experiences, ecological knowledge and recent reward signals (i.e., non-social features) with observed locations, movement directions and successes of others (i.e., social features); and how do the mechanisms underlying this integration lead to adaptive or maladaptive collective outcomes? Payoff information, such as observing a peer successfully catching a fish or filling their basket with mushrooms, is crucial for adaptive decision-making, as it allows individuals to exhibit payoff selectivity—the selective tuning of attentional and behavioral responses depending on others’ current payoffs. Payoff information also influences how information spreads between group members^1,10^. Payoff-biased social learning has been found to influence who individuals learn from in both laboratory experiments^3,13–15^ and real-world scenarios such as programming competitions^16^, underpinning the retention of beneficial behaviors and, thus, driving collective improvements^15,17,18^. However, payoff information is often not directly accessible in many realistic scenarios. Even when available, it frequently provides only an indirect and short-lived cue in temporally and spatially variable environments^2,19^. Moreover, payoff information might shape behavior at different levels and across different time scales, guiding how individuals direct their attention, seek out social cues, or integrate them into their decisions^20^. Lastly, in many scenarios, individuals might also selectively decide whether and when to share payoff information with others^21^, providing group members with additional social information and potentially also benefiting themselves in the long run^22^. It is not well understood how diverse payoff-based processes interact in spatio-temporally variable and uncertain environments to produce adaptive versus maladaptive collective outcomes^23^. Nor is it clear whether—and under what environmental conditions—explicit payoff information is required for collective intelligence, or whether simpler behavioral or location cues suffice.

Most previous experiments relied on relatively simplified paradigms in which participants chose from a limited set of options at fixed intervals and social information was presented in a structured and summarized format^24^. Such paradigms lack the visual–spatial richness of real-world social dynamics, where perceptual and spatial constraints fundamentally shape the costs and benefits of social information use^3,25^. These relatively simplified paradigms typically assume that relevant social features are predefined, instead of requiring participants to infer which features are reliably associated with success^12^. To understand the social-learning mechanisms governing adaptive and maladaptive outcomes in real-world collectives, paradigms that allow complex social dynamics to unfold within naturalistic environments are essential. By “naturalistic”, we do not mean scenarios that are simply more complex or appear more similar to the real world. We specifically refer to heterogeneous and dynamically changing environments characterized by (1) temporal and spatial autocorrelation of (private and social) information^26^, (2) perceptual and spatial constraints^27,28^, and (3) endogenous choice ecologies, where social and non-social features are shaped by one’s own previous actions as well as the actions of others—all key variables of real-world environments that typically lack in behavioral experiments^29^.

We used an online, large-scale immersive-reality approach to study how groups of human participants search for and track a mobile resource. Collective search provides an ideal testbed to study dynamic social learning and collective behavior in a controlled, yet ecologically relevant, context^3,4,9,25,30^. Previous theoretical work and experiments with fish have uncovered the self-organized “collective sensing” phenomenon, where collectives successfully detect and track a rewarding location in the environment using rudimentary processing of personal information^31–33^. Instead of solving the complicated inference problem of tracking shifting environmental gradients, individuals can collectively adapt to complex environments by responding to simple social cues, such as the location of other animals. In a series of experiments mirroring the original work on fish, Hawkins et al. (2023)^34^ showed that without payoff information, human participants could infer the location of a hidden scoring region based on observable movement trajectories, suggesting powerful social inference. This paradigm, however, used an unrealistic bird’s-eye view, representing participants as moving shapes on a flat surface. Participants could directly see when others stopped moving; while reliable, this type of cue for social inference is usually unavailable outside the lab. Moreover, people in the real world often need to balance personal and social information simultaneously. Monitoring one’s own personal reward rates as well as the behavior of others might interfere with the adaptive calibration of social information use. To study collective search and tracking in a more realistic scenario with a limited first-person field of view, realistic spatial constraints, and continuous integration of personal and social information, we developed a 3D immersive-reality experiment that created a natural trade-off between individual exploration of the environment and social learning^3,9,28,35^.

**Fig. 1:**
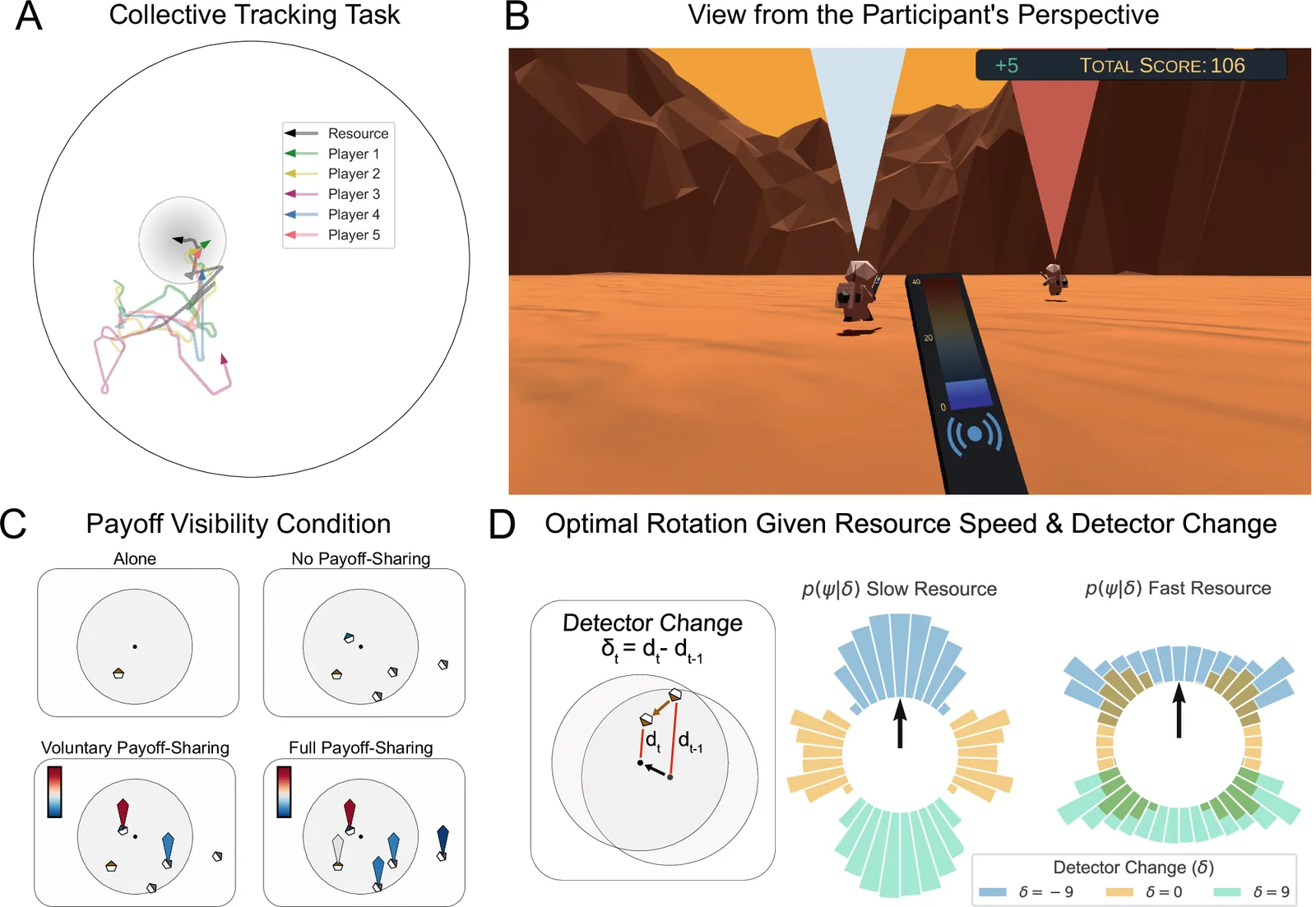
Collective tracking task. **A** Example trajectories of the resource (shaded gray disc) and five participants (colored lines) in a circular arena. Arrows indicate direction of movement. **B** Screen capture from the Voluntary Payoff-Sharing condition. Participants control virtual avatars from a first-person perspective and navigate the arena. Each avatar is equipped with a resource detector showing the rate of point collection (i.e., the proximity to the resource center); dark red (blue) indicates that the participant is close to (far from) the resource center. Total points are indicated in the top right corner. If available, other participants' current payoffs (i.e., their proximity to the resource center) is indicated by a colored beam above their avatar, following the same color scheme as for the detectors. Here, the participant on the right is closer to the resource center, providing high-quality social information. **C** Payoff visibility conditions. Alone: Participants are alone in the environment; No Payoff-Sharing: Participants are in groups of five and can observe others' positions, but not their current payoffs; Voluntary Payoff-Sharing: Participants are in groups of five and can continuously decide whether to share their current payoff (at a cost of 1 point/s); Full Payoff-Sharing: Participants are in groups of five and their payoff information is always shared (at no cost). **D** Left: Participants' private information consists of changes in their detector value (*δ*_*t*_), which indicates the change in distance to the resource center at time *t*. Middle and right: Probabilities of optimal rotation (*ψ*) as a function of detector value change (*δ*) from the previous time step (in seconds), mathematically derived and numerically computed for slow and fast resource movement. While the exact optimal rotation angle cannot be inferred from *δ*, the optimal rotation magnitude follows a simple heuristic: *ψ* scales linearly with *δ* (see Supplementary Note 1 for the mathematical derivation of the optimal rotation model). This means that sharp increase in points call for minimal rotation, whereas sharp decrease call for maximal rotation (3D avatar asset: Stylized Astronaut by PULSAR BYTES, used with permission).

Participants (*n* = 621) were instructed to search and track a hidden moving resource to maximize a monetary reward (Fig. 1; see “Methods” for details; Supplementary Video 1). The closer participants were to its center, the more points they collected. Participants could find and track the resource by looking at their avatar’s detector values, indicating their current distance to the resource center (Fig. 1B; Supplementary Videos 2 and 3). Across four conditions, participants completed the task by themselves (Alone, A), or in groups of 5 where they could see others but had no information about their payoffs (No Payoff-Sharing, NP), where others’ current payoffs were always indicated by a colored sign above their heads (Full Payoff-Sharing, FP), or where participants could freely decide whether and when to share their current payoffs at a small cost (Voluntary Payoff-Sharing, VP). Participants experienced either a slow or fast resource, moving at 40% or 80% of their avatar’s speed, respectively. Fig. 1D shows that the optimal rotation magnitude follows a simple heuristic: A rapid decrease in detector value (indicating moving away from the resource center) should prompt large rotations, while a sharp increase in detector value (indicating moving toward the center) should prompt continued movement in the same direction. The ranges of optimal rotations are narrower for the slow resource because there is less uncertainty about the location of the resource center (see Supplementary Note 1 for the mathematical derivation of the optimal rotation model and Figs. 1D and Supplementary Fig. 2 for illustration).

Based on the collective sensing literature, we predicted that search and tracking would be more efficient for collectives than for solitary individuals, resulting in better performance in the No Payoff-Sharing and Full Payoff-Sharing conditions compared to the Alone condition (see preregistration at AsPredicted: https://aspredicted.org/C62_M6Z). We further predicted that within our dynamically changing immersive environment, the location of others would be insufficient for full social inference, resulting in superior performance in the Full Payoff-Sharing condition compared to the No Payoff-Sharing condition. Finally, simulation work has shown that, under certain conditions, active sharing of payoff information is beneficial to both individuals and collectives in collective search, acting as a recruitment signal that facilitates the self-organization of groups^22^. To explore this, we included a non-preregistered Voluntary Payoff-Sharing condition. We did not formulate prior predictions on their resulting performance.

**Fig. 2:**
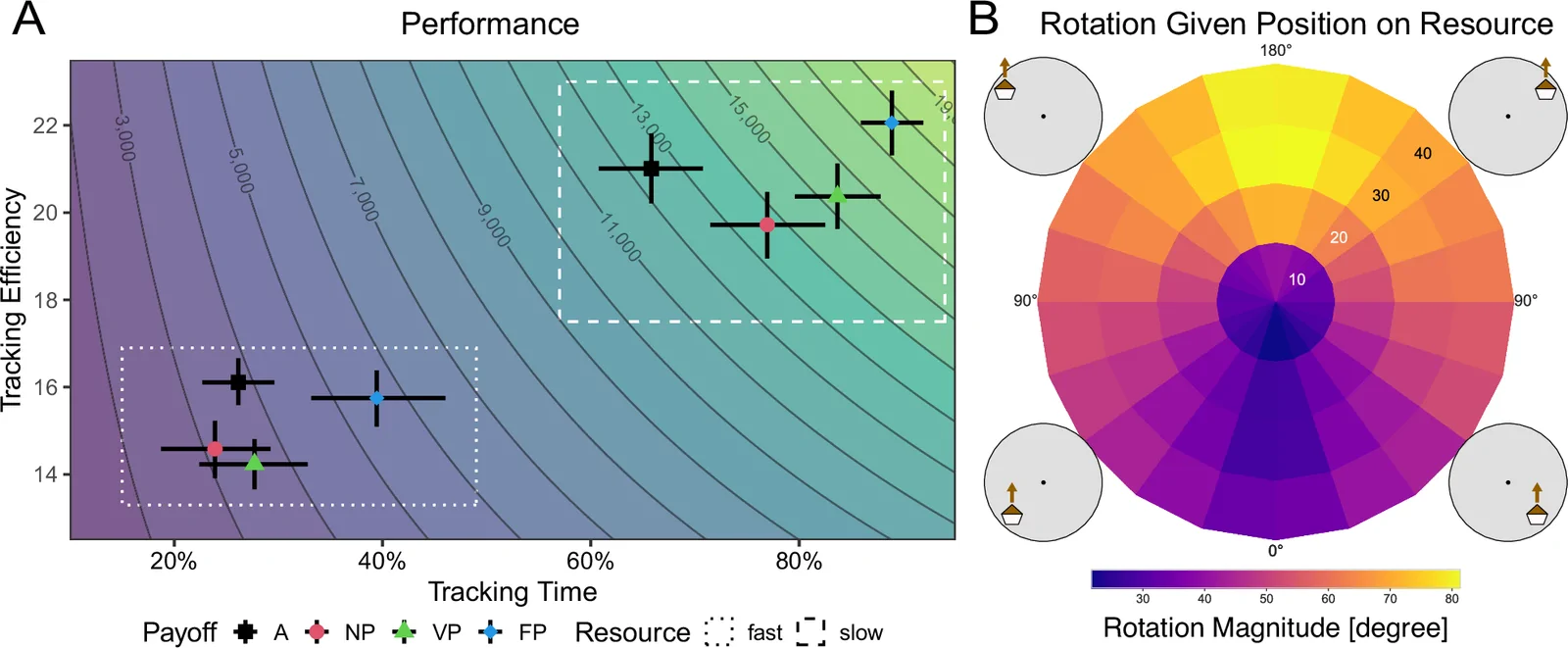
Collective tracking performance. **A** Mean tracking time, tracking efficiency, and total score (colored isoclines) per treatment. The dashed box indicates the slow resource environment, and the dotted box indicates the fast resource environment. A: Alone Condition; NP: No Payoff-Sharing; VP: Voluntary Payoff-Sharing; FP: Full Payoff-Sharing. Dots and error bars show posterior means and 90% highest posterior density intervals (HPDIs). Statistics are derived from *n* = 621 participants (Fast resource: A *n* = 73, NP *n* = 72, VP *n* = 86, FP *n* = 74; Slow resource: A *n* = 71, NP *n* = 80, VP *n* = 79, FP *n* = 86). **B** Participants' average rotation magnitude for different positions on the resource.

**Fig. 3:**
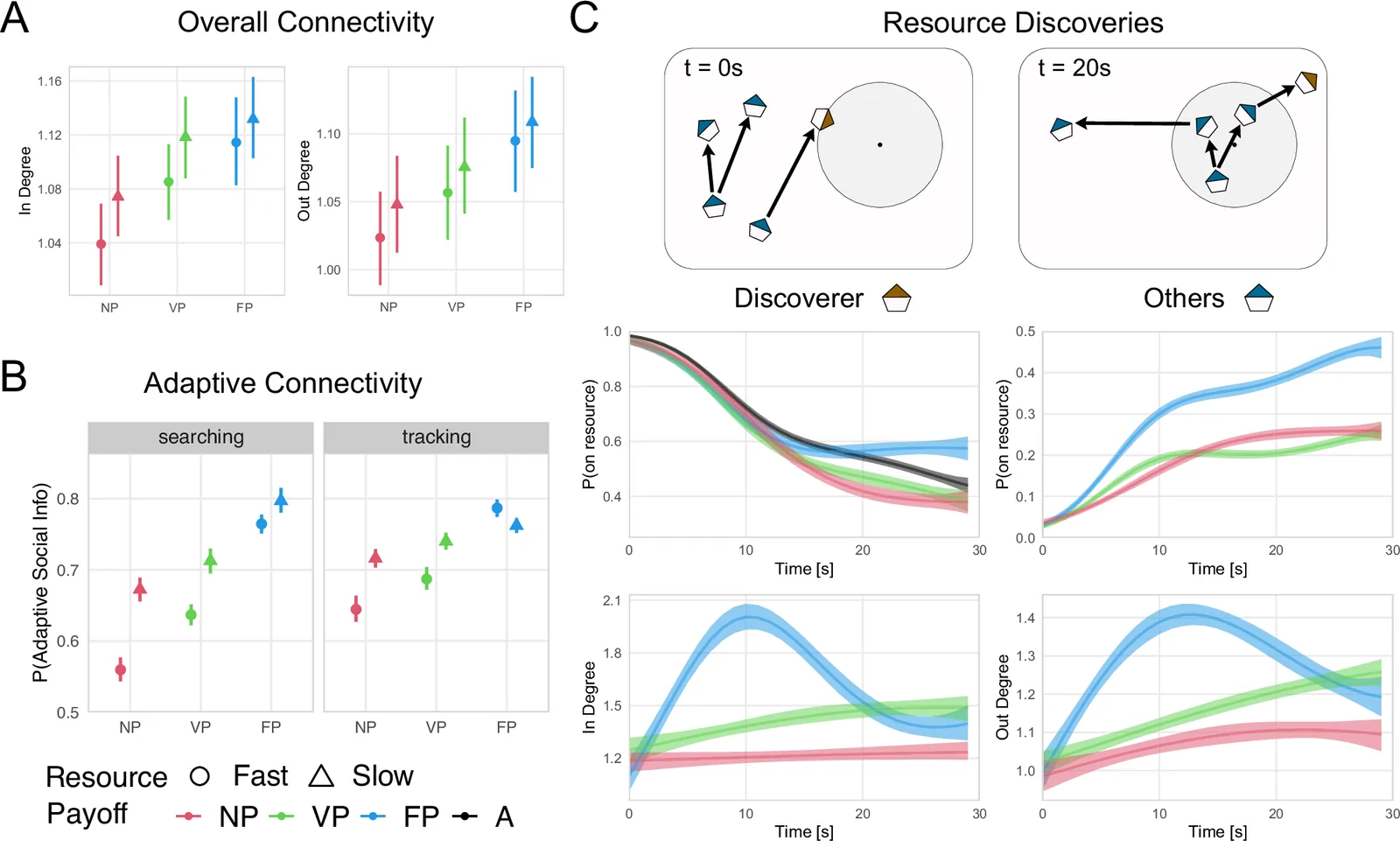
Visibility network analysis. **A** Average in-degree and out-degree per payoff-sharing condition. (B) Probability for participants who have one or more other participants in their field of view that at least one of these others is closer to the resource center than they are (adaptive social information), per payoff-sharing condition and for searching and tracking. For **A** and **B**, data are presented as posterior means and 90% highest posterior density intervals (HPDIs). **C** Top: Illustration of a resource discovery event, where a discoverer (brown) enters the resource and the others (blue) shift their visual attention (indicated by arrows) to the discoverer and approach the resource center 20 s later. Middle left: Probability of remaining on the resource for the initial discoverer, conditional on time since the discovery event. Middle right: Probability that the others (i.e., non-discoverers) are on the resource, conditional on time since discovery. Bottom left: Average in-degree of the discoverer over time. Bottom right: Average out-degree of others over time. Black line indicates participants in the Alone condition. Dark lines show posterior means of a Bayesian Gaussian process regression; shades represent 90% HPDIs. Statistics across all panels are derived from *n* = 621 participants (Group conditions [**A**–**C**]: Fast NP *n* = 72, VP *n* = 86, FP *n* = 74; Slow NP *n* = 80, VP *n* = 79, FP *n* = 86. Alone condition [Panel C]: Fast *n* = 73, Slow *n* = 71).

**Fig. 4:**
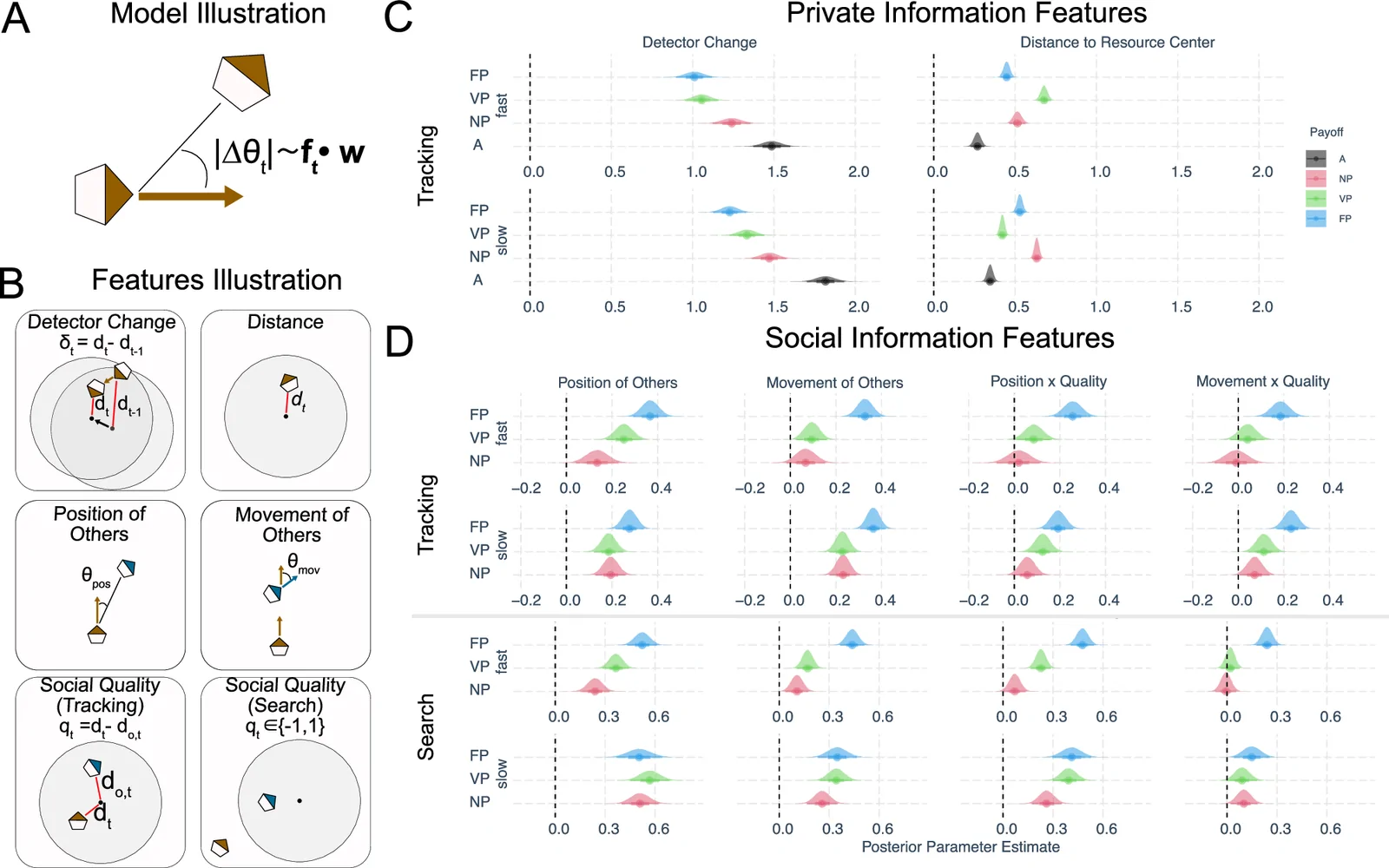
Computational model of movement decisions. **A** Schematic model illustration predicting the absolute rotation magnitude at each point in time based on a linear combination of dynamic private and social features. **B** Illustration of the private and social features used in the model. **C** Posterior distributions of the feature weights for private information features per payoff-sharing and resource condition. **D** Posterior distributions of the feature weights for social information features per payoff-sharing and resource condition. For the half-eye plots in **C** and **D**, the shaded density curves represent the full posterior distributions, the points represent the mean posterior estimates, the thick inner lines represent the 66% highest posterior density intervals (HPDIs), and the thin outer lines represent the 95% HPDIs. Statistics across **C** and **D** are derived from *n* = 621 participants (Fast resource: A *n* = 73, NP *n* = 72, VP *n* = 86, FP *n* = 74; Slow resource: A *n* = 71, NP *n* = 80, VP *n* = 79, FP *n* = 86).

**Fig. 5:**
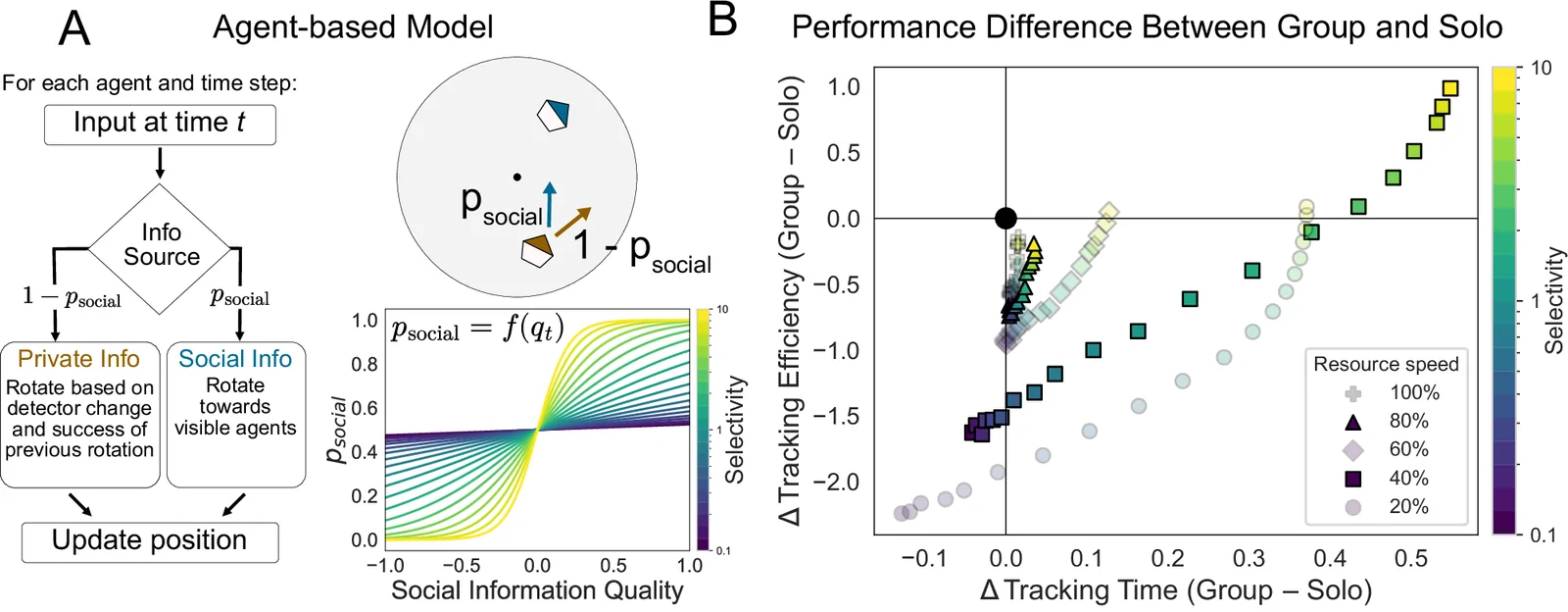
Agent-based simulation results. **A** Model illustration: At each time step, agents rotate based on either private information (with probability 1 − *p*_social_) or social information (with probability *p*_social_). Probability of acting based on social information (*p*_social_) depends on social information quality (*q*_*t*_). If agents followed social information, they rotated towards the center of the locations of other agents in their field of view. If they followed private information, the rotation magnitude was proportional to the change in detector value (*δ*_*t*_) and the rotation direction depended on the outcome of the previous rotation. Bottom right: Agents' selectivity to social information quality; colored lines represent degrees of selectivity. Highly selective agents (yellow) only followed social information if agents in their field of view were performing better than they were at the time; non-selective agents (blue) followed others independently of quality. **B** Average tracking time vs. tracking efficiency of groups relative to solitary agents (black dot). Colors show selectivity levels; opaque points correspond to the two experimental speeds (40% and 80%). Data points in **B** represent the average of *n* = 2000 independent simulation runs per parameter combination.

In this paper, we mechanistically link individual cognition, collective behavior and environmental variables, and show that payoff selectivity is the key driver of collective intelligence in dynamic spatial resource tracking. Concretely, our behavioral analyses (Fig. 2) show that sociality is a double-edged sword: Participants in groups can outperform solitary individuals through both superior tracking and search performance, but only when full payoff information is available; in the absence of payoff information, they sometimes even perform worse, especially when tracking a fast-moving resource. We leverage high-resolution time-series data of participants’ visual information and movement trajectories to study how key events (e.g., resource discoveries) trigger adaptive changes in visibility networks (Fig. 3)—a dynamic directed graph of ‘who sees whom’ at any given moment^36,37^. We find that payoff information boosts performance by allowing collectives to flexibly reorganize visibility networks over time and adaptively guide information flow between group members. We then design a computational model predicting movement decisions on a second-to-second resolution to test how private and social features jointly guide participants’ movement behavior (Fig. 4). Computational results confirm that payoff information unlocks collective intelligence by allowing participants to selectively tune their responses to the position and movement direction of successful peers. Finally, to link our mechanistic insights back to the observed collective dynamics and to gain generalizable insights beyond the specific settings of our experiment, we develop an agent-based simulation, which corroborates our finding that payoff selectivity is indeed the key factor promoting adaptive collective outcomes—but only in certain types of environments (Fig. 5).

## Results

### Performance depends on the social environment and resource speed

We start by comparing performance across the eight treatment conditions to show that collective intelligence only arises under certain circumstances. We use three performance metrics: tracking time (i.e., proportion of time participants spent on the resource), tracking efficiency (i.e., average number of points collected when participants were on the resource), and total number of points, which is a function of both components (color gradient in Fig. 2A). Using hierarchical Bayesian regressions (see Methods), we found that, unsurprisingly, participants in the slow resource environment collected more points than those in the fast resource environment (posterior mean contrast slow-fast = 10,679.04 points, 90% Highest Posterior Density Interval [HPDI] = [9,789.29, 11,544.90] points; Fig. 2A; for all subsequent contrasts reported in the text, values in brackets represent the 90% HPDI; see Supplementary Figs. 3, 5, 6 for participant-level scores, tracking times, and tracking efficiencies).

In the slow resource environment (dashed box in Fig. 2A), participants in all three group conditions collected more points compared to solitary participants (posterior mean contrast NP-A = 1286.73 and 90% HPDI = [ − 790.97, 3366.14] points, posterior mean contrast VP-A = 3137.53 and 90% HPDI = [1126.32, 5079.08] points; posterior mean contrast FP-A = 5236.22 and 90% HPDI = [3373.83, 7129.94] points). Participants in groups with full payoff information (FP) collected more points than participants in groups with no payoff information (posterior mean contrast FP-NP = 3949.48 and 90% HPDI = [1876.26, 6081.79] points), with performance in the voluntary payoff condition (VP) falling in between. Importantly, while the VP condition showed intermediate performance, it did not consistently differ from the NP and A conditions. This lack of a reliable difference was not merely an artifact of the incurred signaling costs. Re-estimating performance using gross scores (i.e., adding the signaling costs back) showed similar qualitative results, as the maximum possible signaling cost was small relative to the overall benefits of full information sharing (see Supplementary Table 3 and Supplementary Note 2 for details on payoff sharing behavior). Groups outperformed singletons mainly because participants in groups spent more time on the resource (posterior mean contrast NP-A = 1.67 and 90% HPDI = [0.56, 2.71] min; posterior mean contrast VP-A = 2.68 and 90% HPDI = [1.75, 3.63] min; posterior mean contrast FP-A = 3.47 and 90% HPDI = [2.63, 4.32] min). Only participants in the FP condition had higher tracking efficiency than singletons (posterior mean contrast = 1.05 and 90% HPDI = [ − 0.02, 2.16] points/sec), whereas participants in the NP and VP conditions tended to perform worse than singletons (posterior mean contrast NP-A =  − 1.29 and 90% HPDI = [ − 2.38,  − 0.16] points/sec; posterior mean contrast VP-A =  − 0.64 and 90% HPDI = [ − 1.73, 0.46] points/sec).

In the fast resource environment (dotted box in Fig. 2A), only participants in FP groups collected more points than solitary ones (posterior mean contrast = 1710.26 and 90% HPDI = [572.81, 2,803.50] points). Participants in VP groups performed on par with singletons (posterior mean contrast =  − 298.66 and 90% HPDI = [ − 1169.61, 537.86] points), and participants in NP groups performed worse than singletons (posterior mean contrast =  − 834.66 and 90% HPDI = [ − 1654.19,  − 35.59] points). The superior performance of participants in FP groups resulted from them spending more time on the resource while achieving equal tracking efficiency compared to singletons. Participants in NP and VP groups, on the other hand, had similar tracking times but substantially worse tracking efficiency than singletons, resulting in worse overall performance (see Supplementary Tables 2, 6, and 7 for statistical comparisons of scores, tracking times, and tracking efficiencies). Participants in the FP condition had the lowest variability in points collected in both resource environments, whereas single participants had the highest (Supplementary Table 5), suggesting that collective foraging can buffer the risks of searching for resources in dynamic environments^38^. Performance at both the individual and group levels remained stable over the course of the experiment (see Supplementary Fig. 4 and Supplementary Table 4 for individual and Supplementary Fig. 30 and Supplementary Table 16 for collective performance comparisons). To explicitly quantify collective intelligence, we compared interacting groups against synthetic “nominal groups” of randomly assembled solitary participants. Measuring the median distance to the resource demonstrated that FP groups cohesively outperformed this baseline, remaining substantially closer to the center in both fast (posterior mean contrast = 20.77 and 90% HPDI = [9.91, 31.46] m) and slow (posterior mean contrast = 13.58 and 90% HPDI = [9.69, 17.35] m) environments (Supplementary Fig. 29, Supplementary Table 15). In sum, these results suggest that in slow-changing environments, it is beneficial to be in a group. In fast-changing environments, however, merely observing the location of others may lead to maladaptive use of social information, which can only be offset by additional payoff information.

Our mathematical derivation (Fig. 1D; Supplementary Note 1) revealed a simple heuristic for optimal rotation for tracking the resource: Rotate if the detector value decreases, and continue in the same direction if it increases. Across conditions, participants followed this heuristic (Fig. 2B; see Supplementary Fig. 7 for condition-specific results). When they were moving towards the center of the resource, they displayed a relatively small angle of rotation; when they were moving away from the center, they displayed a large angle of rotation.

### Payoff information shapes the structure and adaptability of visibility networks

We used visibility network analysis to show that payoff information increased adaptive visual connectivity and boosted performance by allowing collectives to flexibly reorganize visibility networks over time^36,37^. A visibility network is a time-varying, directed graph where an arrow *i* → *j* at time *t* means *i* could see *j* at time *t* (see “Methods” for details). Due to the spatial constraints of the environment, participants could see at least one other group member for about 57% of the task, most frequently observing just one or two peers (see Supplementary Table 12). At both resource speeds, participants in FP groups had higher overall connectivity, with both higher in- and out-degrees, than did participants in NP and VP groups (Fig. 3A; see Supplementary Tables 8 and 9 for statical comparisons). This suggests that participants looked more at others when information about others’ payoffs was available. In all conditions, we observed a strong negative correlation between participants’ in- and out-degrees (Supplementary Fig. 9), replicating previous results from an immersive foraging experiment with static rewards^3^. This suggests an asymmetry in the flow of social information, with some participants being frequently observed by others while rarely observing others themselves, and other participants frequently observing others while rarely being observed themselves. Participants’ in-degree and out-degree were systematically related to their performance in almost all conditions (Supplementary Fig. 10). Participants who collected more points paid little visual attention to others (low out-degree), but received a lot of visual attention from others (high in-degree). High-performing participants became focal points. These effects were strongest in the FP conditions, and weakest in the NP conditions, suggesting that the availability of high-quality social information provided more scope for adaptive selective visual attention.

We next quantified the differences in quality of social information across conditions. To capture the potential for beneficial social information use, for all times when participants had one or more other participants in their field of view, we calculated the probability that at least one of these other participants was closer to the resource center than the focal participant. Across resource speeds, and when both searching and tracking, participants in the FP groups were more likely to observe others in better positions than themselves compared to participants in the NP and VP groups suggesting increased payoff selectivity in visual attention (difference in probability posterior mean contrasts fast/searching: posterior mean contrast FP-NP = 0.20 and 90% HPDI = [0.18, 0.23], posterior mean contrast FP-VP = 0.13 and 90% HPDI = [0.11, 0.15]; slow/searching: posterior mean contrast FP-NP = 0.12 and 90% HPDI = [0.10, 0.15], posterior mean contrast FP-VP = 0.08 and 90% HPDI = [0.06, 0.11]; fast/tracking: posterior mean contrast FP-NP = 0.14 and 90% HPDI = [0.12, 0.16], posterior mean contrast FP-VP = 0.10 and 90% HPDI = [0.08, 0.12]; slow/tracking: posterior mean contrast FP-NP = 0.05 and 90% HPDI = [0.03, 0.06], posterior mean contrast FP-VP = 0.02 and 90% HPDI = [0.01, 0.04]; Fig. 3B; see Supplementary Table 10 for statistical comparisons). In sum, payoff information increased overall social connectivity (Fig. 3A), as well as the adaptiveness of social connectivity (Fig. 3B).

We studied the temporal adaptation of visibility networks by analyzing how participants changed their visual attention in situations where one participant discovered the resource while the others were searching for it (Fig. 3C; see “Methods” for details). The frequency of such events was much higher in the fast resource environment (829 instances for fast vs. 261 for slow; see Supplementary Table 11), since participants were more likely to lose (and rediscover) the resource when it moved quickly. We therefore focus on the fast resource environments in the main text (see Supplementary Fig. 12 for results for the slow environments). In the FP condition, participants who discovered the resource (the “discoverer”) received much more visual attention (i.e., in-degree) shortly after discovery—peaking approximately 10 s after discovery—compared to discoverers in NP and VP groups (posterior mean contrast FP-NP = 0.80 and 90% HPDI = [0.72, 0.88] in-degree; posterior mean contrast FP-VP = 0.62 and 90% HPDI = [0.54, 0.70] in-degree; Fig. 3C, bottom left). The other participants (“others”) increased their visual attention to others (i.e., out-degree) after the resource discovery. Comparing the in-degree of discoverers and others shows that others oriented specifically toward the discoverer (see Supplementary Figs. 13, 14). Again, this effect was much stronger for participants in the FP groups compared to participants in the NP and VP groups (posterior mean contrast FP-NP = 0.32 and 90% HPDI = [0.29, 0.36] out-degree; posterior mean contrast FP-VP = 0.27 and 90% HPDI = [0.23, 0.30] out-degree; Fig. 3C, bottom right). Notably, the VP condition also revealed distinct outcomes depending on whether the discoverer shared their payoff or not, replicating between-condition differences: Discoverers who shared their payoffs received more visual attention and others increased their outward attention (see Supplementary Fig. 15 for fast resources and Supplementary Fig. 16 for slow resources), suggesting that payoff sharing triggered adaptive network responses.

This reconfiguration of visibility networks after resource discoveries had tangible adaptive consequences. The probability that “others” would be on the resource increased more markedly over time in the FP condition compared to the other conditions (Fig. 3C, top right). Moreover, this greater availability of local social information allowed discoverers in FP groups to stay on the resource for longer compared to solitary individuals who discovered the resource, whereas discoverers in other group conditions lost it sooner (Fig. 3C, top left; see Supplementary Figs. 15, 16 for corroborating results comparing sharing and non-sharing events in the VP condition). These results underscore how continued access to payoff information enabled FP groups to selectively adapt their visibility networks over time based on observed payoffs and achieve a higher tracking time than participants in NP and VP groups (Fig. 2A). Others were able to capitalize on the discoveries of group members, and even discoverers themselves benefited from the recruitment of social information to their local environment.

### Computational model of movement decisions

To show how behavioral outcomes arose from participants’ fine-grained decisions, we developed a computational model of movement decisions that formalizes hypotheses about the relative importance participants place on different private and social cues (Fig. 4). We modeled participants’ absolute changes in movement direction as a linear combination of private and social features. We used the magnitude of rotations instead of directions because our mathematical derivations showed that changes in detector values only provide information on how much an optimal agent should rotate, and not in which direction (see Supplementary Note 3 and Supplementary Figs. 17, 18 for an analysis of rotation direction that reaches the same main conclusions). Search and tracking were analyzed in separate models because different sets of features were available for each state.

#### Private information features

To study how participants responded to their personal rewards, we quantified two features: The distance feature reflects a participant’s distance to the resource center, which they can infer from their absolute detector value, and the detector change feature reflects the change in this distance since the previous time step (in seconds), which is the key factor determining optimal tracking according to our analytical derivation. Note that both features were only available to participants when they were on the resource.

#### Social information features

To study how participants responded to different visible social cues, we quantified four social features. The position of others represents the angular difference between a participant’s heading direction and the average position of others within a participant’s field of view, thus indicating where others are. The movement direction of others reflects the angular difference between a participant’s heading direction and the average movement direction of others within their field of view, indicating where others are going. We also included the interactions between each of these features and social information quality. For tracking, social information quality is computed as the difference between one’s own distance to the center and the average distance of visible others. For searching, the value of social information quality is set to 1 if another visible participant is currently on the resource and to −1 otherwise. These interactions allow for payoff selectivity, the adaptive tuning of social information use depending on the performance of others (which may or may not be directly visible, depending on the condition). All features were normalized to a range between [ − 1, 1] to allow for comparisons of all shared features across conditions.

#### Model weights

When tracking the resource, participants in all conditions rotated more if they were further from the resource center and if their distance to the center had increased within the last second (i.e., their payoffs decreased; see Fig. 4B). Participants responded most strongly to detector changes in the alone condition; participants in the FP groups responded least strongly. This reduced reliance on private cues in the presence of increasingly reliable forms of social information confirms the attentional and/or behavioral trade-off between private and social cues and raises a key question: If optimal rotation during tracking is purely determined by the current change in payoffs and FP groups were least sensitive to this key piece of private information, how were they able to outperform all other conditions in slow environments and match singletons in fast environments?

The answer lies in their more selective use of social information. Overall, participants used social information to inform their movement choices (all social information feature weights greater than 0) and social information features influenced participants’ rotation decisions similarly across both tracking and search states and across both resource speeds (Fig. 4C). However, there were crucial differences among payoff sharing conditions. Participants in all group conditions relied on the position of others: They rotated more if others were in the periphery of their field of view. Participants in FP groups tended to respond most strongly to others’ positions (except when searching in the slow resource environment). Mirroring the behavioral results, the behavior of participants in VP groups tended to fall in between that of the FP and NP groups. Participants also relied on the movement direction of others, rotating more if their movement direction differed greatly from the average movement direction of others in their field of view. Once again, participants in the FP condition responded most strongly to the movement direction of others compared to NP and VP conditions, and the performance of participants in VP groups tended to fall in between that of the FP and NP groups.

Next, we turn to the interaction between the social features and social information quality in order to examine the payoff selectivity in participants’ reliance on social information. Participants in FP groups flexibly tuned their social information use, responding more strongly to the position and movement direction of others if they provided higher-quality information. In the slow resource environment, participants in NP groups, where payoff information was not explicitly shared, also adjusted their behavior based on the payoffs of others. This finding suggests that they were able to infer the success of others solely based on their behavior—albeit to a limited degree. In the fast resource environment, however, only participants with visible payoff information could flexibly adjust their reliance on the behavior of others, suggesting important limits to social inference in fast-changing environments. More generally, differences in use of social cues between payoff-sharing conditions were much more pronounced in fast resource environments (both during tracking and searching) than in slow resource environments.

To identify the adaptive consequences of decision-making, we used the random effects of the feature weights from the multilevel computational model (visualized in Supplementary Figs. 19 and 20) to predict success in terms of tracking efficiency and tracking time (see Supplementary Note 4). Higher detector sensitivity was generally associated with higher tracking efficiency across all conditions. Following others’ positions was associated with lower tracking efficiency, whereas aligning with others’ movement direction was associated with higher efficiency (see Supplementary Fig. 21), suggesting that where others were going was a more reliable cue for success than where they were. Importantly, payoff selectivity with respect to both position and movement direction of others was reliably associated with tracking efficiency, again confirming that selective tuning of social information use depending on payoffs was crucial for success (see Supplementary Fig. 22 for results on searching that confirm the core insights).

Finally, to test whether participants’ strategies evolved over the course of the 15-minute session, we divided the data into two temporal epochs (first vs. second half) and re-estimated our computational models with temporal interaction terms. We found a general, slight decrease in baseline rotation over time, potentially reflecting increased proficiency or fatigue. Participants in the group conditions generally became more reliant on social information and used social features more selectively during search as the experiment progressed, while their decision weights during the tracking phase remained stable overall (see Supplementary Note 5, Supplementary Figs. 23–24, Supplementary Tables 13–14).

In sum, the computational model highlights the critical role of payoff selectivity in shaping participants’ movement decisions. When payoff information was available, participants overall relied more on social cues. They modulated their social information use depending on information quality by selectively using social cues when others were performing better than themselves. The model also allowed us to examine how flexibly participants integrated social and private information features, revealing a dynamic trade-off based on contextual factors. While our main model captures the average influence of social cues, the physical structure of the observed crowd could further modulate how these cues are weighted. To test this, we fitted an extended model to instances where participants observed multiple peers simultaneously, assessing the impact of crowd size and movement cohesion (see Supplementary Note 6). We found that participants dynamically adjusted their heuristics based on crowd structure: when observing highly cohesive groups, participants devalued the group’s spatial position and were more influenced by the group’s unified movement direction (Supplementary Figs. 25–26). This demonstrates that participants selectively utilized movement cues when the crowd exhibited behavioral consensus. Additionally, we verified that physical distance does not devalue social cues; a supplementary analysis showed that participants were robustly influenced by—and in the FP condition, even more responsive to—distant peers during search (see Supplementary Note 6 and Supplementary Fig. 27).

### A mechanistic agent-based simulation

To confirm that payoff selectivity was the key causal factor determining collective outcomes and to gain generalizable insights beyond the two resource environments and group size studied in the experiment, we developed a mechanistic agent-based simulation of the task. At each simulation time step, agents decided on their next movement direction based on either private or social information, with the social probability (*p*_social_) depending on the quality of available social cues (i.e., the difference in current payoffs between agents and observed others), and their selectivity in responding to those cues (Fig. 5A). High selectivity meant that agents only used social information if others were doing better than they were at the time. If agents followed social information, they compromised between their previous movement direction and the average position of others. When agents instead relied on private information (with probability 1 − *p*_social_), they followed the heuristic identified before (see Figs. 1D and 2B): They continued in the same direction if they were approaching the resource center and rotated if they moved away from the center (see “Methods” for a detailed description).

Figure 5 B plots tracking time and tracking efficiency for social agents compared to solitary agents (large black dot) across a range of resource speeds (shape) and degrees of selectivity (color). Unsurprisingly, agents that were highly selective in their social information use spent more time on the resource and had a higher tracking efficiency. While agents in groups outperformed singletons across a broad range of selectivity values in terms of tracking time, only highly selective social agents outperformed singletons in terms of tracking efficiency. The costs and benefits of sociality were also modulated by the speed of the resource. In fast-moving environments (80% and 100% of agents’ speed), social agents spent slightly more time on the resource compared to singletons, with only small effects of selectivity. In slow-moving environments (20% and 40% of agents’ speed), selectivity had a much greater influence on tracking time: Social agents spent either less or substantially more time on the resource compared to singletons, depending on the degree of selectivity. Crucially, social agents outperformed singletons in both tracking time and efficiency only when the resource moved at a moderately slow pace (40% of agents’ speed) and when social information was used selectively.

These simulation results largely match our experimental findings and help explain them in more detail. In our experimental slow-moving environment (with the resource moving at 40% of agents’ speed), participants in FP groups outperformed singletons in both performance metrics (Fig. 2A), providing a direct match with the only resource speed at which this is predicted to happen for highly selective agents. In the simulation, relatively non-selective social agents could also spend more time on the resource than singletons, confirming the empirical finding that—at least in slow environments—participants in NP and VP groups outperformed the tracking time of singletons. However, non-selective agents could not match the tracking efficiency of singletons either empirically or in the simulation. Additional simulations (see Supplementary Note 7 and Supplementary Fig. 28) showed that the benefits of selectivity were strongest for imperfect individual trackers. This result suggests that selective social learning is particularly consequential when private cues are unreliable or ambiguous. Agents with high detector sensitivity performed well with less-selective social strategies, indicating that strong reliance on private information could mitigate the risks of maladaptive social influence. Finally, we tested whether the results are consistent across different group sizes (ranging from 3-99; see Supplementary Fig. 31). While the core results remained the same, larger group sizes led to an increased risk of maladaptive herding for non-selective agents.

## Discussion

Flexible social learning is often a shortcut to better decision making. It underlies humans’ collective adaptability and, ultimately, our success as a species^5,6,10^. Yet little is known about how collective intelligence emerges in naturalistic mobile human groups dealing with dynamically changing environments. There is also limited knowledge of how people in such complex tasks integrate dynamic personal and social features, and how these strategies interact with environments, leading to adaptive or maladaptive collective outcomes.

Our immersive-reality experiment shows that groups can outperform singletons in such complex environments, under specific conditions: Only when participants could observe others’ payoffs continuously did groups outperform singletons. Contrasting previous findings from the collective-sensing literature in animals^31^ and in humans using more abstract experiments^34^, groups performed worse than singletons when they only had access to the locations of others—especially when tracking a fast-moving resource. Visibility network analysis and computational modeling confirmed that these differences in collective outcomes stem from the way people balance private and social cues, with full payoff information allowing for rapid reconfiguration of visibility networks and selective tuning of social information use. Indeed, previous immersive experiments have also failed to discover performance benefits of collectives compared to singletons^3,9^. By systematically manipulating payoff visibility, we are able to mechanistically demonstrate the specific conditions required for collective intelligence to emerge.

To be successful in our paradigm, participants needed to find efficient strategies for both searching the resource and tracking its center. Our analysis reveals that payoff selectivity is key for both components of adaptive behavior. During the search phase, when a participant discovered the resource, payoff information allowed groups to flexibly adjust their visual connections and orient themselves towards the discoverer, enabling them to rapidly converge on the resource location^39^. In contrast, without reliable payoff information, resource discoveries went mostly unnoticed by other group members, making it impossible to reap the collective benefits of costly search^25^. During the tracking phase, payoff information allowed participants to flexibly exploit social information, relying on others’ behavior only when it provided adaptive information. In fact, payoff selectivity allowed participants in groups to achieve a higher tracking efficiency compared to solitary individuals (Fig. 2A), while being less sensitive to otherwise essential private information about changes in reward rates (Fig. 4C). They did this by following both the locations and movement directions of others, primarily when group members were better positioned than they were. Furthermore, when facing multiple peers, participants dynamically modulated their reliance on the positions and movement direction of others based on the cohesion of the observed crowd. They relied more heavily on the movement direction of the cohesive crowd, devaluing their spatial position. Similarly, physical distance did not strictly devalue social cues; participants actively utilized information from distant peers during search, likely because they provided valuable information about previously unexplored areas. Because, unlike previous experiments, our task involved an inherent trade-off between private and social information use, following others without a quality filter backfired in groups without reliable payoff information, lowering their performance compared to singletons. Moreover, this maladaptive overuse of social information was amplified by greater environmental volatility: Since the resource location moved faster, positional cues were no longer reliable predictors of future resource locations, whereas visible payoffs still provided real-time information on the environmental target, thereby ensuring collective adaptation even in rapidly changing conditions.

Our agent-based simulations not only confirmed the decisive role of payoff selectivity for collective performance, they also clarified the role of the environment. During search, when private information is absent, low-quality social information also provides beneficial hints about resource location, resulting in collective improvements in tracking time (i.e., time on the resource) across a wide range of environments. During tracking, participants need to strategically balance private detector feedback and social cues, limiting the environments in which social information—even if used selectively—is beneficial. When the resource moves slowly, private information is sufficient for tracking the resource and social cues offer little additional value. When the resource moves quickly, on the other hand, reliance on potentially outdated social cues at the expense of private information impairs tracking and frequently leads to losing the resource. Between those extremes lies an ecological “sweet spot”—corresponding to the slow-resource condition in the experiment—where social information use not only benefits searching but also tracking the resource. Because private cues can be ambiguous and mapping observed detector changes to optimal movement decisions can be cognitively demanding, simply copying high-performing neighbors overcomes this deficiency and increases performance beyond the level of singletons.

Future work could systematically vary group size, which proved important for collective-foraging dynamics in simulations^25,27^, in order to investigate how the costs and benefits of social information use across environments might change as groups become larger. Moreover, we only considered groups of identical agents. Systematically examining the role of individual heterogeneity within groups—both synthetic and human—may reveal a more complex picture of the optimality and trade-offs that individuals face in collective search and tracking. Furthermore, future studies that integrate fully immersive virtual reality headsets and eye-tracking could build on these findings to gain a more detailed understanding of how visual salience, gaze behavior and apparent object size continuously influence social attention. Importantly, by showcasing a way of studying human behavior beyond the highly constrained paradigms often used in cognitive science, psychology, and economics, our work also paves the way towards analyses of collective human adaptation outside of the lab, for example, during social foraging^4^.

In line with simulation work predicting that active payoff sharing can evolve by recruiting group members as potentially useful sources of social information^22^, results from the Voluntary Payoff-Sharing condition showed that participants were indeed willing to pay a personal cost to actively share payoff information with others. This active payoff sharing not only resulted in more adaptive visual information flow following resource discoveries and more selective tuning of social information use, but also improved overall performance compared to groups without the ability to share payoffs. By imposing a small individual cost for sharing information, we introduced a social dilemma reflecting the free-rider problem and the tragedy of the commons, ensuring a tangible trade-off compared to a no-cost scenario. Because the value of social information in our task is highly context-dependent—for example, signaling a lack of reward is useful during search, but signaling far from the resource during the tracking phase is not—determining a strictly optimal signaling baseline is complex. In this study, we focused on a relatively short time frame yet found indications of temporal adaptation in collective behavior strategies in both signaling (Supplementary Note 2) and information usage patterns (Supplementary Note 5). This suggests an exciting opportunity for further research into the interplay between individual and collective learning dynamics and how these strategies evolve during longer, multi-session interactions. Such experimental setups could also shed light on the multi-generational cultural evolutionary processes underlying many real-world cultural adaptations, e.g., ref. ^40^. Future work could also explore sharing tendencies in groups of different sizes—simulations predict sharing to decline in larger groups^22^—to isolate whether payoff sharing is driven by reciprocity, normative pressures within the group, or stable altruistic tendencies. Intriguingly, collective foraging has recently been posited as a powerful framework to model the evolution of prosocial communication and human language^41^, generating predictions that can be tested using our framework.

Multiple—only partly overlapping—definitions of the term “collective intelligence” have been used in the literature, spanning multiple disciplines^6^. Woolley et al. (2010)^42^, for example, focus on the transferability of group performance across domains independently of individual ability, and Burton et al. (2024)^43^ concentrate on the comparison of performance between solitary individuals and groups, with collective intelligence being evidenced by participants in groups, on average, outperforming singletons. Especially strict definitions of collective intelligence demand that groups must show performance levels that go beyond the capacity of single individuals^44^. While our work does not assess the transferability of collective performance across tasks^42^, both our experimental and simulation work demonstrates the conditions under which groups perform better (or worse) than solitary individuals (in line with Burton et al. (2024)^43^). Importantly, it is this increase in average performance (together with the observed reduction in variance^45^) that is key for natural or cultural selection to favor increased reliance on social cues. The experiment did not provide evidence for collective intelligence in the strictest sense as exceeding the capacity of any individual (see Supplementary Fig. 3), most likely due to the increased attentional and cognitive demands of simultaneously integrating multiple sources of private and social information in a novel task. Still, alleviating such demands, our agent-based simulations revealed that the identified processes can, in principle, also generate performance levels exceeding the capacities of even the most-skilled individuals.

Beyond introducing a naturalistic 3D paradigm for studying dynamic collective adaptation and providing computational tools for analyzing complex social interaction data, the core contribution of our study lies in mechanistically linking individual cognition, collective behavior and environmental variables, explaining the emergence of adaptive or maladaptive collective outcomes. This work demonstrates that collective intelligence does not automatically emerge under naturalistic conditions as soon as multiple individuals are grouped together—instead, it depends on the specific way that personal and social cues are integrated and is sensitive to environmental volatility. These results mirror results from geography and economics demonstrating how socially optimal or suboptimal location decisions arise from the dynamic acquisition and processing of limited information as well as changing network influences—albeit at very different temporal and spatial scales^46,47^. By combining behavioral and dynamic visibility-network analyses with fine-grained computational modeling and agent-based simulations, we provide multiple complementary lines of evidence identifying payoff selectivity as the key driver of collective intelligence in spatial search and tracking. These findings pinpoint the conditions under which shared information transforms a group of individuals into an intelligent collective, and pave the way for future investigations of social interactions in naturalistic groups.

## Methods

### Participants and design

This research complies with all relevant ethical regulations and was approved by the Institutional Review Board of the Max Planck Institute for Human Development (number: A 2022-26). Data collection was conducted over three periods in 2023. On April 11-12, data were collected for the A, NP, and FP conditions (both fast and slow). On May 3, data were collected for the VP condition (both fast and slow). Roughly half of the samples within each condition were collected in these first two periods (*n*=329 participants). The remaining half of the samples in all conditions were collected from November 13–17. We recruited 796 participants online from Prolific (aged 18–65 years, fluent in English, with a minimum Prolific approval rate of 95%), who provided informed consent before starting the experiment. We restricted the age to 18–65 years to minimize variance from differences in motor skills. For each experimental condition (e.g., Full Payoff Sharing and slow resource), participants were recruited separately within a given time interval, typically 2 h. To maximize the number of complete groups, we opened 50 slots simultaneously per experimental condition on Prolific. This number was dictated by the bandwidth capabilities of the game server. Participants could only join newly forming groups before the start of the experiment; once a session had begun, no additional participants could enter. If a participant dropped out during the experiment, this participant was not replaced.

We removed participants who did not complete the experiment (*n* = 21); who had a very low frame rate ( > 5% below the target of 10 Hz), causing faulty rendering of the environment (*n* = 27); who did not move for more than 25% of the experiment (*n* = 29); who were assigned to group conditions but could not be matched within 10 min (*n* = 59); and who were in groups with more than one invalid participant (*n* = 39). This resulted in a final sample size of 621 (female: 239; male: 381; prefer not to say: 1; *M*_age_= 33.56, *SD*_age_ = 10.17; see Supplementary Table 1 for an overview of the number of participants for each of the eight conditions). Demographic information, including sex and age, was determined via self-reporting on the Prolific platform. Sex and gender were not considered in the study design, and sex- and gender-based analyses were not performed, as the primary focus of the study was on general mechanisms of collective spatial search and tracking. Participants earned a baseline of £3 for completing the study and could earn a bonus of up to £2 based on their individual performance (i.e., total score). Participants in the top 10% of performance within each condition received a £2 bonus, participants in the 10–50% range received a £1 bonus, and participants in the 50–75% range received a £0.50 bonus. Our study protocol was preregistered on April 5, 2023 (https://aspredicted.org/C62_M6Z, AsPredicted 127762). We deviated from the preregistered protocol in four instances. First, we added a non-preregistered Voluntary Payoff-Sharing condition to explore the dynamics of active payoff sharing without prior performance predictions. Second, the sample size was increased from 260 to 621 participants to maximize the number of complete groups. Third, we adjusted our exclusion criteria by raising the inactivity threshold to 25% (as the 5% threshold was overly restrictive for a 15 min collective search task) and implementing technical exclusions for low frame rates to prevent hardware limitations from skewing behavior. Finally, instead of analyzing the simple average distance from the resource center, we assessed performance via tracking time, tracking efficiency, and total score to provide a more detailed, mechanistic understanding of search and tracking behaviors. Because these updated metrics encompass the originally planned distance variable, no originally planned analyses were omitted.

Participants completed 15 min of the task in a 4 × 2 between-subjects design (Fig. 1C). Participants were allotted to one of four payoff conditions, completing the task by themselves (Alone, A), in groups where others’ locations but not payoffs were available (No Payoff-Sharing, NP), in groups where others’ payoffs were also available (Full Payoff-Sharing, FP), or in groups where participants could decide whether to share their payoff information (Voluntary Payoff-Sharing, VP). A group size of five was chosen for all interactive conditions because it provides a balanced rate of social encounters (see Supplementary Table 12), aligns with standards in interactive group experiments e.g., refs. ^3,9,13,48^, and mitigates the technical risks of network latency and participant disconnection that are inherent to larger, real-time, 3D, multiplayer setups. In the NP condition, participants could observe and interact with their group members in real time, but could not see how many points they were currently collecting. In the FP condition, they could also observe their group members’ current payoffs (indicated by a colored beam above avatars; see Fig. 1). Participants could thus directly observe the resource quality at each group member’s location, provided they were in their field of view. In the VP condition, participants could continuously decide whether they wanted to share their current payoff information or not at a cost of 1 point per second. Sharing was initiated by pressing the space bar, which activated a visible payoff beam above the avatar, similar to the Full Payoff-Sharing condition. Once activated, sharing continued until the space bar was pressed again, with each sharing episode lasting at least one second, even if participants immediately stopped.

Participants were tasked to search and track either a slow or fast resource. The slow resource was fairly easy to follow: It moved at 2.8 m/s, corresponding to 40% of participants’ speed. The fast resource was fairly difficult to follow: It moved at 5.6 m/s, corresponding to 80% of participants’ speed. The resource movement followed a Lévy walk, with step lengths drawn from a power-law distribution with a shape parameter *α* = 1 (limited to steps between 1 and 100 m), and movement directions sampled from a uniform distribution^49–51^. If the resource center approached the boundary of the environment, the movement direction was resampled. Qualitatively, resource movement consisted of frequent short steps and infrequent long steps (an example of a resource movement trajectory is available at https://youtu.be/pHE5aOMb1_M or as Supplementary Video 1).

### Experimental procedure

After reading the instructions and passing comprehension checks, participants completed a tutorial to familiarize themselves with the task and controls. Participants in group conditions then entered a virtual waiting room in order to be matched with other participants. The experiment started after a group of five was formed. Participants who could not be matched within 10 min were financially compensated and removed from the experiment.

In the experiment, participants were immersed in a 3D environment, freely navigating an astronaut avatar from a first-person perspective (90^∘^ horizontal and 60^∘^ vertical field of view; these values are the default settings in Unity game engine^52^) through a circular crater on a distant planet (400 m diameter; see Fig. 1 and videos of the experiment at https://youtu.be/AcfIw_qWR_Q and https://youtu.be/xY7nzh4vF4o, or Supplementary Videos 2 and 3). This field of view also imposes perceptual constraints on participants, who must actively rotate in order to gather social information.

Participants could move left, forward, or right using the “A,” “W,” and “D” keys, respectively, or using the corresponding arrow keys. They could move at 7 m/s and rotate at 150^∘^/s, and were instructed to locate and track a hidden, mobile resource in order to collect points and maximize their bonus payment. The resource was circular and had a 40 m radius, yielding a payoff of 40 points/s in the center, linearly decreasing to one point/s at the edge. Such distance-dependent payoffs reflect the fundamental properties of foraging systems where the exploitation potential of a patchy resource is greatest in its center and decreases towards the periphery^53^. Avatars held a resource detector indicating the resource quality (i.e., payoff) at their current location (Fig. 1B). Movement towards the resource center was indicated by increasingly higher values and deeper reds on the resource detector. At the beginning of the experiment, the resource was placed in the center of the arena and participants were initialized on the resource facing its center.

The study was implemented using the Unity game engine (v2021.3.14f1)^52^ and compiled to run in a web browser using WebGL technology. The resulting WebGL build was integrated into a web interface using React JS (v18.2.0) and Next.js (v13.1.51) (via the React Unity WebGL (v9.4.0) module) and hosted on an NGINX server. The astronaut avatar was based on the ‘Stylized Astronaut’ model by PULSAR BYTES. PlayFab^54^ provided the game server responsible for synchronizing the participants. Communication between the client and the server was handled by the Mirror (v2022.10.0) Unity package^55^. Participant data was recorded locally and uploaded to the PlayFab server at the end of the experiment for the alone condition. For the group conditions, data was recorded on the PlayFab game server, alongside the resource traces.

#### Data

At a sampling interval of 10 Hz, we recorded the positions (i.e., x- and z-coordinates) of the resource center and participants’ avatars, the rotations (around the y-axis) of participants’ avatars, the scores that participants received at each time step, and whether or not the participants were sharing their payoffs at that moment (in the Voluntary Payoff-Sharing condition). From this raw data, we reconstructed the movement trajectories and visual information of each participant using Python 3.9^56^ and Python libraries numpy (v1.24.2)^57^, pandas (v1.5.3)^58^, and xarray (v2023.4.0)^59^.

### Data analysis

#### Behavioral analyses

To quantify how participants’ performance differed among experimental conditions while also accounting for participant- and group-level variability, we applied multilevel Bayesian Beta regression models to our three performance measures: tracking time, tracking efficiency, and total number of points. Tracking time represents the proportion of time participants spent on the resource. Tracking efficiency was calculated as the average number of points collected when participants were on the resource.

#### Dynamic visibility network analysis

To analyze the structure of social interactions, we constructed dynamic visibility networks for each group at each one-second interval ^36,37^. (Note that our spatial definition of visibility networks differs from the use of ‘visibility graphs’ in time-series analysis; e.g., ref. ^60^). In our dynamic visibility network and computational models, visual access was defined strictly based on the 90^∘^ forward-facing field of view, without imposing a maximum distance cut-off. This design choice reflects the visual rendering of the virtual environment, which lacks atmospheric perspective, fog, or physical obstacles, allowing avatars—and particularly the bright payoff beams in the FP and VP conditions—to contrast strongly against the barren landscape, maximizing their saliency. A participant *j* was considered visible to participant *i* if *j* was within *i*’s 90^∘^ forward-facing field of view. This condition is met if the absolute angular difference between participant *i*’s heading direction (*θ*_*i*_) and the angle towards participant *j* (*θ*_*i*→*j*_) is less than 45^∘^^25,35,37^:

1 $$\left\vert {\theta }_{i\to j}-{\theta }_{i}\right\vert < 4{5}^{\circ }.$$

The angle from *i* to *j* is calculated from their respective positions, **p**_*i*_ = (*x*_*i*_, *y*_*i*_) and **p**_*j*_ = (*x*_*j*_, *y*_*j*_), as:

2 $${\theta }_{i\to j}={{{\rm{atan2}}}}({y}_{j}-{y}_{i},{x}_{j}-{x}_{i}).$$

This pairwise check for all participants at each time step generated a directed graph (a dynamic network), where nodes represent participants and a directed edge from *i* to *j* indicates that *i* could see *j*. From these networks, we computed two key metrics for each participant at each time step: in-degree (the number of individuals observing them) and out-degree (the number of individuals they observed). These networks were constructed and analyzed using the NetworkX (v3.0) Python package ^61^.

To analyze overall network connectivity (Fig. 3A) we applied a Bayesian Poisson regression; to analyze adaptive connectivity (Fig. 3B) we applied a Bayesian logistic regression, in both cases accounting for individual- and group-level heterogeneity.

*Resource discovery events*. To investigate the temporal dynamics of visibility network adaptation, we analyzed resource discovery events in which one group member, the “discoverer,” discovers the resource while the other group members are searching for it (Fig. 3C). These events were characterized by a specific set of conditions: all group members were searching (i.e., were not on the resource) for at least 5 s, and exactly one participant entered the resource and stayed on it for at least 5 s. For these events, we analyzed changes in visibility and performance in the 30 s following the resource discovery event (see Supplementary Fig. 11 for an illustration). In case of overlapping events, only the first event was used. We used Gaussian process function learning through the gp function in brms^62^ to estimate how visibility networks and performance changed over time for the “discoverer” and for “others.”

#### Computational model of rotation decisions

To better understand individual movement decisions at a mechanistic level, we developed a computational modeling framework that sequentially predicts participants’ movement decisions based on a linear combination of private and social information features. Movement decisions consist of two components: rotation direction (whether participants turn clockwise or counterclockwise) and rotation magnitude (how strongly they adjust their direction). We present the analysis of rotation direction in the Supplementary Note 3; in the main text, we focus on rotation magnitude, which can be expressed more directly in relation to private and social information features. Although behavior was recorded at 10 Hz, for modeling purposes we discretized it into 1 s intervals. At each interval, the absolute rotation magnitude is modeled as following the framework in refs. ^3,4,9^:

3 $$| \Delta {\theta }_{t}| \sim {{{\rm{Exponential}}}}({\lambda }_{t}),\qquad \log {\lambda }_{t}={w}_{0}+{{{{\bf{f}}}}}_{t}^{\top }{{{\bf{w}}}},$$

where **f**_*t*_ represents the vector of private and social features observed at time *t* and **w** denotes the corresponding feature weights. The components of **f**_*t*_ are introduced in the following subsections. The model assumes that participants integrate both types of information to decide how much to deviate from their current movement direction. This framework allows us to quantify the relative influence of different, dynamically changing private and social cues across experimental conditions (see Fig. 4 for illustrations).

*Private information features*. The private information features capture how participants responded to changes in their own detector readings and positions relative to the resource. These features were only present during tracking phases.

The first private information feature is the detector change (*δ*_*t*_), which represents the change in distance to the resource center between consecutive time steps:

4 $${\delta }_{t}={d}_{t}-{d}_{t-1},$$

where *d*_*t*_ is the detector value (distance to the resource center) at time *t*. A negative detector change (*δ*_*t*_ < 0) indicates a decrease in distance (i.e., moving closer to the resource), while a positive detector change (*δ*_*t*_ > 0) indicates an increase in distance (i.e., moving away from the resource). According to our analytical derivation, optimal trackers should rotate more if their detector change indicates movement away from the resource center and less if the detector indicates movement towards the center (see Supplementary Note 1). This feature is thus critical for tracking across experimental conditions, as it directly informs participants about performance.

The second private feature is the absolute distance to the resource center (*d*_*t*_). This feature does not provide additional directional information, but it can still influence behavior, as indicated by the behavior rotation analysis (Fig. 2B).

*Social information features*. The model also incorporates a set of social features that participants might use in both tracking and search phases. These features are calculated based on the average position and average direction of motion of others within the focal participant’s field of view. We used an averaged metric to ensure model parsimony, as the empirical distribution of visibility events indicated that encountering large clusters within the limited field of view was relatively infrequent. Specifically, while participants observed one or two other peers for  ≈ 42% of the total time, encounters with larger groups of three or four individuals accounted for only  ≈ 15% of the time (see Supplementary Table 12). However, to account for instances where multiple peers were visible, we conducted supplementary analyses incorporating both crowd size and movement cohesion to ensure that simple averaging did not obscure higher-order structural effects (see Supplementary Note 6).

The first social feature is the position of others (*θ*_pos,*t*_), representing the angular difference between the focal participant’s heading direction and the average position of others within the field of view:

5 $${\theta }_{{\mbox{pos}},t}=| {\theta }_{{\mbox{group}},t}-{\theta }_{t}|,$$

where *θ*_*t*_ is the current heading direction of the focal participant and *θ*_group,*t*_ is the angle towards the average position of visible others. Letting the participant’s position be **p**_*t*_ = (*x*_*t*_, *y*_*t*_) and the average position of the *N* visible others be $({\bar{x}}_{t},{\bar{y}}_{t})$, this angle is calculated as

6 $${\theta }_{{\mbox{group}},t}={{{\rm{atan2}}}}({\bar{y}}_{t}-{y}_{t},{\bar{x}}_{t}-{x}_{t}).$$

Larger values indicate that others are positioned at a larger angle from the current direction of motion of the focal participant. If participants use this feature, they rotate less when others are near the center of their field of view and more when others are toward the periphery of their field of view.

The second social feature is the movement direction of others (*θ*_mov,*t*_), reflecting the angular difference between the focal participant’s heading direction and the average movement direction of others in the field of view:

7 $${\theta }_{{\mbox{mov}},t}=| {\bar{\theta }}_{m,t}-{\theta }_{t}|,$$

where ${\bar{\theta }}_{m,t}$ is the average movement direction of others in the field of view at time *t*. Large values indicate that others are moving against the focal participant’s current direction, while small values indicate that others are moving in the same direction as the focal participant. This feature might serve as a proxy for others’ beliefs about the location of the resource, as the observer can infer the assumed location of the resource center based on the direction others are moving in.

*Social information quality*. To investigate how social information quality influences participants’ reliance on social cues across conditions, we included interactions of both social features with social information quality. For tracking, relative social information quality (*q*_*t*_) is defined as

8 $${q}_{t}={d}_{t}-{\bar{d}}_{o,t},$$

where *d*_*t*_ represents the participant’s distance from the resource center and ${\bar{d}}_{o,t}$ represents the average distance of others within the participant’s field of view from the resource center. Positive values (*q*_*t*_ > 0) indicate that others are closer to the resource center, while negative values (*q*_*t*_ < 0) indicate that others are farther away from the resource center.

For searching, relative social information quality is represented as a binary variable,

9 $${q}_{t}\in \{-1,1\},$$

where *q*_*t*_ = 1 indicates that the average position of others in the focal participant’s field of view is on the resource at time *t*, and *q*_*t*_ = − 1 indicates they are not.

Note that social information quality could only be directly and consistently accessed in the FP experimental condition (via the colored beam). In the NP condition it had to be inferred.

To ensure that shared feature weights could be interpreted across conditions and to facilitate model convergence, all features were normalized to a range between [ − 1, 1].

#### Model fitting

All models were fitted in a Bayesian framework using Hamiltonian Monte Carlo in Stan^63^, implemented in R (v4.5.3) with the brms (v2.22.0)^62^. We analyzed the data using Bayesian multilevel models, including varying intercepts for experimental groups or individual participants to account for the nested structure of the data. We used weakly informative, regularizing priors for all parameters, specifically ${{{\mathcal{N}}}}(0,0.5)$ for population-level coefficients, ${{{\mathcal{N}}}}(0,1)$ for intercepts, and appropriate weakly informative distributions for variance and dispersion parameters (e.g., Gamma or half-Normal). These priors were selected to provide moderate regularization and restrict predictions to plausible ranges, which we verified using prior predictive checks. Posterior predictive checks and visual inspection of the trace plots, and Gelman-Rubin convergence diagnostics (all $\hat{R}$ statistics within acceptable ranges) confirmed model convergence. To summarize the posterior distributions, we report the posterior means and 90% highest posterior density intervals (HPDIs) of the differences between conditions (i.e., posterior mean contrasts) using tidybayes (v3.0.7)^64^ and emmeans (v1.11.1)^65^ R packages following Heiss (2021) ^66^. The tidyverse (v2.0.0)^67^ packages were used for performing data manipulation and generating the final visualizations.

### Agent-based simulation

To link the identified mechanisms to the observed collective dynamics and extend our results beyond the specific settings of the experiment, we developed an agent-based simulation, mirroring the structure of the experiment using the mesa (v2.2.4) framework^68^. In the simulations, agents are assumed to search for and track a mobile resource, which moves according to the same Lévy-walk process used in the experiment.

Agents are assumed to move at a constant speed *v*, updating their position **p**_*t*+1_ at each time step *t* based on their heading angle *θ*_*t*_^69,70^:

10 $${{{{\bf{p}}}}}_{t+1}={{{{\bf{p}}}}}_{t}+v\cdot \Delta t\cdot \left(\begin{array}{r}\cos ({\theta }_{t})\\ \sin ({\theta }_{t})\end{array}\right),$$

where **p**_*t*_ = (*x*_*t*_, *y*_*t*_) is the position at time *t*, and *Δt* is the time step duration. During tracking (i.e., when both private and social information is available), the agent’s new heading angle *θ*_*t*+1_ is determined based on its current heading *θ*_*t*_ and a chosen orientation change *Δθ*_*t*_:

11 $${\theta }_{t+1} \sim {{{\mathcal{N}}}}({\theta }_{t}+\Delta {\theta }_{t},{\sigma }^{2})$$

where *σ* = 10^∘^, and *Δθ*_*t*_ is chosen probabilistically between social and private information:

12 $$\Delta {\theta }_{t}=\left\{\begin{array}{ll}\Delta {\theta }_{t}^{\,{\mbox{social}}\,},\quad &\,{\mbox{if }}R < {p}_{{{{\rm{social}}}}},\\ \Delta {\theta }_{t}^{\,{\mbox{private}}\,},\quad &\,{\mbox{if }}R\ge {p}_{{{{\rm{social}}}}},\end{array}\right.$$

where *R* ~ *U*(0, 1), $\Delta {\theta }_{t}^{\,{\mbox{social}}\,}$, and $\Delta {\theta }_{t}^{\,{\mbox{private}}\,}$ are the agent’s orientation change based on social and private information respectively.

For private information, the agent first determines the magnitude of rotation, $\Delta {\theta }_{t}^{\,{\mbox{private (mag)}}\,}$, based on the change in distance to the resource, *δ*_*t*_ = *d*_*t*_ − *d*_*t*−1_, :

13 $$\Delta {\theta }_{t}^{\,{\mbox{private (mag)}}\,}={\beta }_{0}+{\beta }_{1}{\delta }_{t},$$

where *β*_1_ controls sensitivity to the change in distance and *β*_0_ is the baseline rotation magnitude. For all simulations ${\beta }_{0}=\max ({\beta }_{1},5{0}^{\circ })$. The agent then decides whether to continue turning in the same direction or reverse. The probability of reversing direction, *p*_change direction_,

14 $${p}_{{{{\rm{change}}}}\,{{{\rm{direction}}}}}=\left\{\begin{array}{ll}{p}_{{{{\rm{success}}}}},\quad &\,{\mbox{if }}\,{\delta }_{t} < {\delta }_{t-1},\\ {p}_{{{{\rm{failure}}}}},\quad &\,{\mbox{if }}\,{\delta }_{t}\ge {\delta }_{t-1},\end{array}\right.$$

so that the final orientation change, $\Delta {\theta }_{t}^{\,{\mbox{private}}\,}$, is determined:

15 $$\Delta {\theta }_{t}^{\,{\mbox{private}}\,}=\left\{\begin{array}{ll}-{{{\rm{sgn}}}}(\Delta {\theta }_{t-1})\cdot \Delta {\theta }_{t}^{\,{\mbox{private (mag)}}\,},\quad &\,{\mbox{if }}{R}_{2} < {p}_{{{{\rm{change}}}}\,{{{\rm{direction}}}}},\\ {{{\rm{sgn}}}}(\Delta {\theta }_{t-1})\cdot \Delta {\theta }_{t}^{\,{\mbox{private (mag)}}\,},\quad &\,{\mbox{if }}{R}_{2}\ge {p}_{{{{\rm{change}}}}\,{{{\rm{direction}}}}},\end{array}\right.$$

where *R*_2_ ~ *U*(0, 1), *p*_success_ = 0.15 and *p*_failure_ = 0.35 based on the human behavioral results (see Supplementary Note 3), and *Δθ*_*t*−1_ is the net orientation change from the previous time step.

Social information biases the rotation of an agent towards the average position of its visible neighbors. The probability of using social information *p*_social_ is calculated using a logistic function of the relative quality of social information *q*_*t*_ e.g., ref. ^13^:

16 $${p}_{{{{\rm{social}}}}}=\frac{1}{1+\exp (-k{q}_{t})},$$

where *k* controls selectivity to social information quality, and *q*_*t*_ is calculated by comparing the agent’s own distance to the resource with the average distance of its visible neighbors:

17 $${q}_{t}={d}_{t}-{\bar{d}}_{o,t}$$

Here, *d*_*t*_ is the focal agent’s distance to the resource at time *t*, and ${\bar{d}}_{o,t}$ is the average distance of visible neighbors to the resource. A positive *q*_*t*_ indicates the agent is farther from the resource than its neighbors, making social information more valuable.

Orientation change based on social information is defined by the difference between the agent’s current heading direction *θ*_*t*_ and the angle towards the average location of other agents in its field of view, *θ*_group,*t*_:

18 $$\Delta {\theta }_{t}^{\,{\mbox{social}}}={\theta }_{{\mbox{group}},t}-{\theta }_{t}$$

As in the experiment, the field of view is set to 90^∘^.

When searching (i.e., when no private information is available), agents follow a socially biased correlated random walk influenced by social information within their field of view. Social information is treated similarly to tracking, except that the relative quality of social information (*q*_*t*_) is binarized based on whether others are on the resource (*q*_*t*_ = 1) or not (*q*_*t*_ = − 1), as in the computational model. When not acting based on social information, the rotation magnitude is uniformly sampled from $U(0,{r}_{\max })$ and the direction is randomly chosen. Here, ${r}_{\max }$ represents the maximum rotation at each time step when the agent was searching and is set to ${r}_{\max }=9{0}^{\circ }$ for all simulations.

### Reporting summary

Further information on research design is available in the Nature Portfolio Reporting Summary linked to this article.

## Supplementary information

Supplementary Information

Description of Additional Supplementary Files

Supplementary Movie 1

Supplementary Movie 2

Supplementary Movie 3

Reporting Summary

Peer Review File

## Data Availability

The raw time series data, metadata, and processed tables of computed measures generated in this study have been deposited in the Open Science Framework (OSF) database (https://doi.org/10.17605/OSF.IO/ZRHWT) ^71^. An online tool to visualize the raw time series data is available at https://vagechirkov.github.io/CollectiveSearchOnMarsTraceViewer/.

## Code availability

All custom code used for the analysis and simulation in this study has been deposited in the OSF database under accession code https://doi.org/10.17605/OSF.IO/ZRHWT^71^. A readable, rendered version of the full analysis pipeline is available at https://vagechirkov.github.io/collective-tracking-analysis/.
