# Supplementary material for: Payoff selectivity drives collective intelligence during dynamic resource tracking in humans
Source: Nat Commun. 2026 Sep 23;17:10133. doi: 10.1038/s41467-026-77762-z (PMC13601515; doi:10.1038/s41467-026-77762-z)
Supplement: Supplementary file 1 [file 41467_2026_77762_MOESM1_ESM.pdf]

Supplementary Information for

Payoff selectivity drives collective intelligence during dynamic resource tracking in humans

Valerii Chirkov[1,2,3*], Ralf H.J.M. Kurvers[1,2], Pawel Romanczuk[2,3] & Dominik Deffner[1,2,4*]

[1]Center for Adaptive Rationality, Max Planck Institute for Human Development, Berlin, Germany

[2]Science of Intelligence, Research Cluster of Excellence, Berlin, Germany

[3]Institute for Theoretical Biology, Department of Biology, Humboldt Universität zu Berlin, Berlin, Germany

[4]Department of Psychology, Philipps-University Marburg, Marburg, Hesse, Germany

[*]Corresponding authors: valerii.chirkov@hu-berlin.de; dominik.deffner@uni-marburg.de

Contents of Supplementary Information

Supplementary Figures **3**

Fig. 1: Optimal Rotation Model . . . 3
Fig. 2: Optimal Rotation for Different Resource Speeds . . . 4
Fig. 3: Collected Points . . . 4
Fig. 4: Collected Points: Temporal Contrast . . . 5
Fig. 5: Tracking Time . . . 6
Fig. 6: Tracking Efficiency . . . 7
Fig. 7: Average Rotation Magnitude . . . 7
Fig. 8: Voluntary Payoff-Sharing Condition . . . 8
Fig. 9: In-Degree and Out-Degree Relationship . . . 9
Fig. 10: Participant's Distance Rank Relationship to Degree . . . 10
Fig. 11: Resource Discovery Events Illustration . . . 11
Fig. 12: Resource Discovery Events (slow resource) . . . 12
Fig. 13: Resource Discovery Events (fast resource; in-/out-degree full) . . . 13
Fig. 14: Resource Discovery Events (fast resource; in-/out-degree full) . . . 14
Fig. 15: Resource Discovery Events (VP; fast resource) . . . 15
Fig. 16: Resource Discovery Events (VP; slow resource) . . . 16
Fig. 17: Rotation Direction vs. Detector Change . . . 17
Fig. 18: Rotation Direction vs. Social Information . . . 18
Fig. 19: Rotation Magnitude Random Effects - Tracking . . . 19
Fig. 20: Rotation Magnitude Random Effects - Search . . . 20
Fig. 21: Rotation Magnitude Random Effects vs. Tracking Efficiency . . . 21
Fig. 22: Rotation Magnitude Random Effects vs. Tracking Time . . . 22
Fig. 23: Rotation Magnitude Temporal Dynamics - Tracking . . . 23
Fig. 24: Rotation Magnitude Temporal Dynamics - Search . . . 24
Fig. 25: Rotation Magnitude Cohesion & Out-degree Features - Tracking . . . 25
Fig. 26: Rotation Magnitude Cohesion & Out-degree Features - Search . . . 26
Fig. 27: Posterior Estimates for Social Features by Peer Distance - Search. . . . 27

Fig. 28: Effect of Social Information Selectivity (ABM) 28
Fig. 29: Average Median Distance to Resource In Groups 28
Fig. 30: Average Median Distance to Resource In Groups: Temporal Contrast 29
Fig. 31: Effect of the Group Size (ABM) 29

Supplementary Tables 30
Table 1: Number of Participants per Condition 30
Table 2: Posterior Estimates Collected Points 31
Table 3: Posterior Estimates for Gross Collected Points (Signaling Costs Reimbursed) 32
Table 4: Posterior Estimates for Collected Points: Temporal Contrasts 33
Table 5: Posterior Estimates Collected Points Precision 34
Table 6: Posterior Estimates Tracking Time 35
Table 7: Posterior Estimates Tracking Efficiency 36
Table 8: Posterior Estimates In Degree 36
Table 9: Posterior Estimates Out Degree 37
Table 10: Posterior Estimates Adaptive Connectivity 38
Table 11: Number of Resource Discovery Events per Condition 39
Table 12: Percentage of Time Participants Have Others in Their FOV 39
Table 13: Posterior Estimates for Temporal Dynamics - Tracking 40
Table 14: Posterior Estimates for Temporal Dynamics - Search 41
Table 15: Posterior Estimates Average Median Distance 42
Table 16: Posterior Estimates Average Median Distance: Temporal Contrast 42

Supplementary Note 1: Optimal rotation based on private information 43
1.1 Forward model: calculating distance change 43
1.2 Optimal rotation 44
1.3 Inference model: inferring rotation from distance change 44

Supplementary Note 2: Voluntary Payoff-Sharing Condition 45

Supplementary Note 3: Rotation Direction Analysis 46

Supplementary Note 4: Rotation Magnitude Model Random Effects Analysis 47

Supplementary Note 5: Rotation Magnitude Model Temporal Analysis 47

Supplementary Note 6: Rotation Magnitude Model Additional Social Information Features 48

Supplementary Note 7: Interaction Between Private Sensitivity and Social Selectivity (ABM) 49

Supplementary Figures

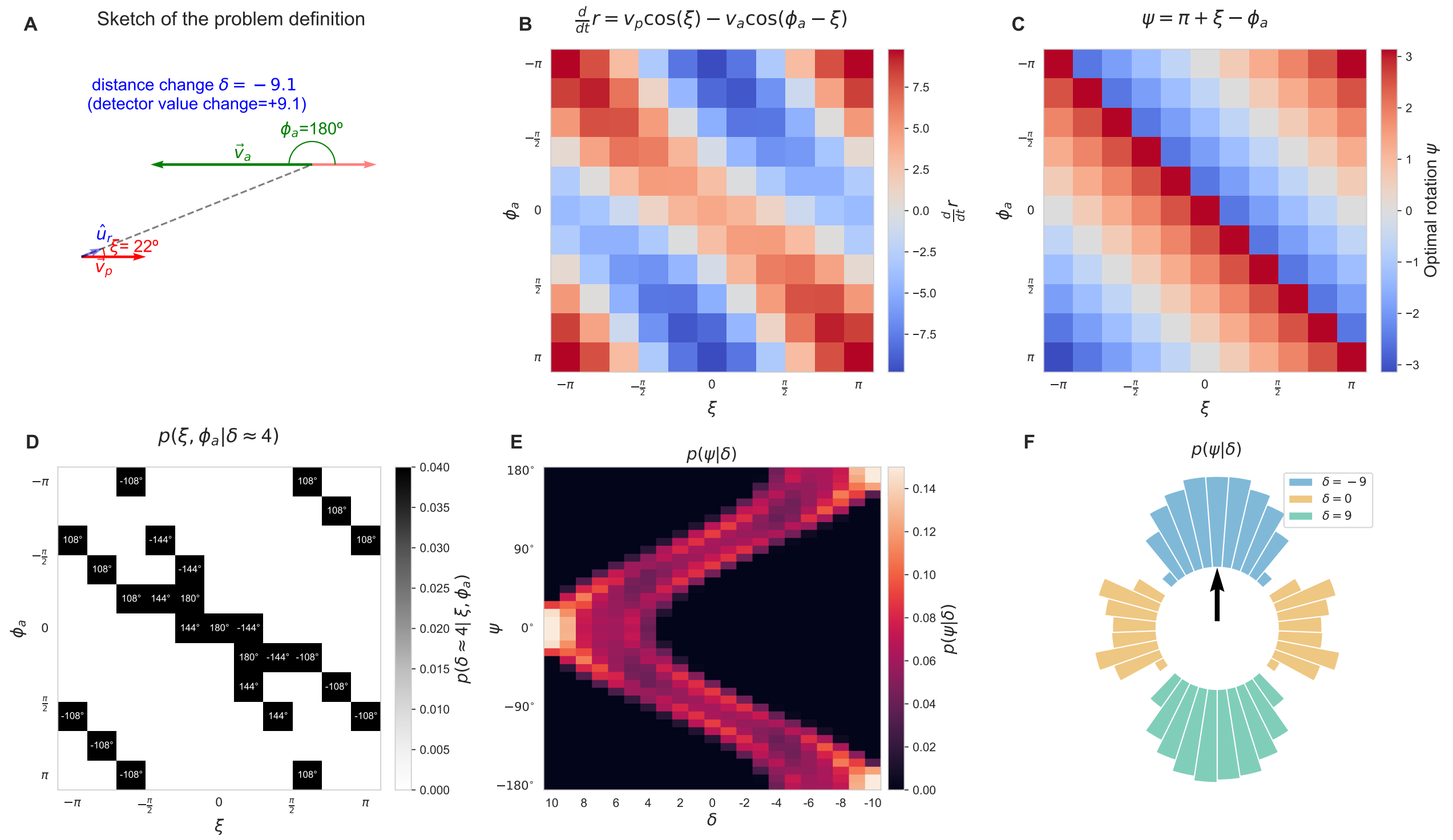

Figure 1: Forward and Inverse Model. **A** Schematic of the forward model's geometry. An agent (velocity $\mathbf{v}_a$) tracks a moving resource (velocity $\mathbf{v}_p$). The model is defined in the resource's reference frame, where ϕ_a is the agent's heading relative to the resource's direction of motion, and ξ is the angle of the agent's position relative to that same direction. **B** The forward model output: the rate of change in distance (dr/dt) shown as a function of the agent's relative heading (ϕ_a) and position (ξ). **C** The optimal rotation (ψ) required to intercept the resource, calculated for each combination of ϕ_a and ξ (see Eq. 9). **D** The core ambiguity the inverse model must solve. For a single observed distance change (e.g., $\delta = 4$), multiple combinations of the unknown angles ϕ_a and ξ are possible. Each of these combinations corresponds to a different optimal rotation (values shown in cells). **E** The inverse model's solution: the posterior probability distribution $P(\psi|\delta)$ over all possible rotation angles (y-axis), given distance change δ (x-axis). **F** The same posterior distribution $P(\psi|\delta)$ visualized in polar coordinates for $\delta \in \{-9, 0, 9\}$. All panels are generated using a resource speed of $v_p = 0.4\ v_a$, which corresponds to the slow resource condition in the experiment.

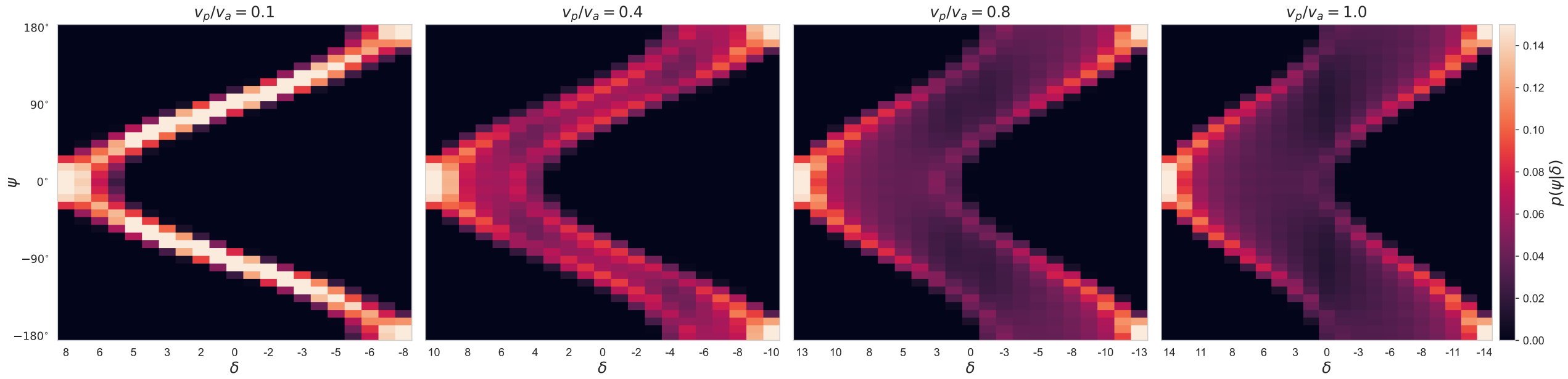

Figure 2: Inference Model $P(\psi|\delta)$ Across Varying Resource Speeds. This figure illustrates the output of the inverse model (posterior probability $P(\psi|\delta)$) for different resource speeds, relative to the agent's speed (v_p/v_a). Panels show the inferred optimal rotation ψ given an observed change in distance δ, when the resource moves at speed: 0.1 v_a, 0.4 v_a, 0.8 v_a, and 1.0 v_a. Each subplot displays the probability distribution over optimal rotations for a range of observed distance changes. As the resource speed increases, the landscape of optimal rotations can become more complex, reflecting the increasing challenge of inferring the correct turn from ambiguous sensory information.

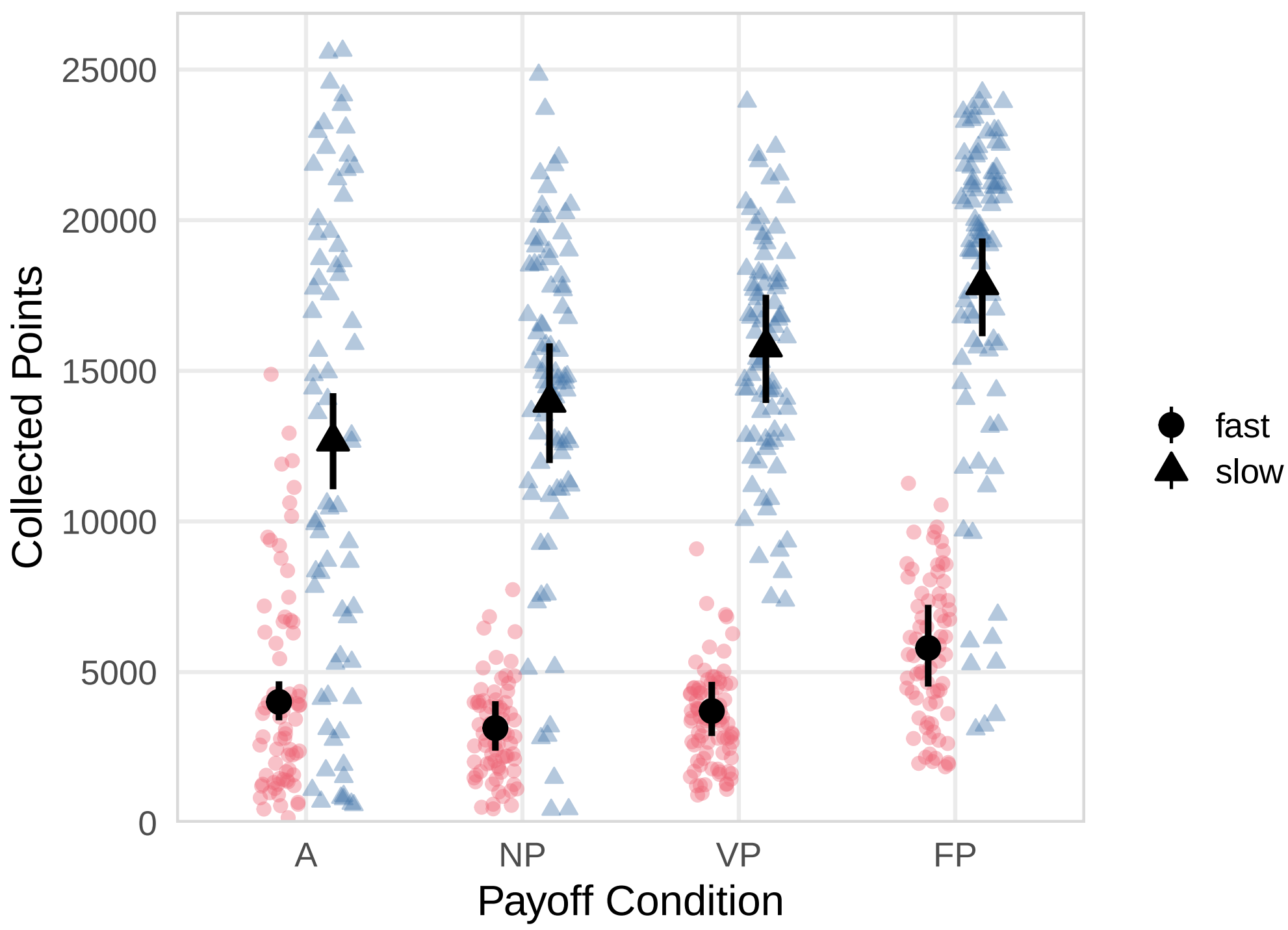

Figure 3: Collected Points. Total number of collected points per treatment, shown for each payoff condition (A: Asocial Condition; NP: No Payoff-Sharing; VP: Voluntary Payoff-Sharing; FP: Full Payoff-Sharing) and resource speed (slow vs. fast). Each dot represents an individual participant. Larger black dots and error bars show posterior means and 90% HPDIs. The numerical values for the contrasts between the conditions are shown in the Table 2 and 5. Statistics are derived from $n = 621$ participants (Fast resource: A $n = 73$, NP $n = 72$, VP $n = 86$, FP $n = 74$; Slow resource: A $n = 71$, NP $n = 80$, VP $n = 79$, FP $n = 86$).

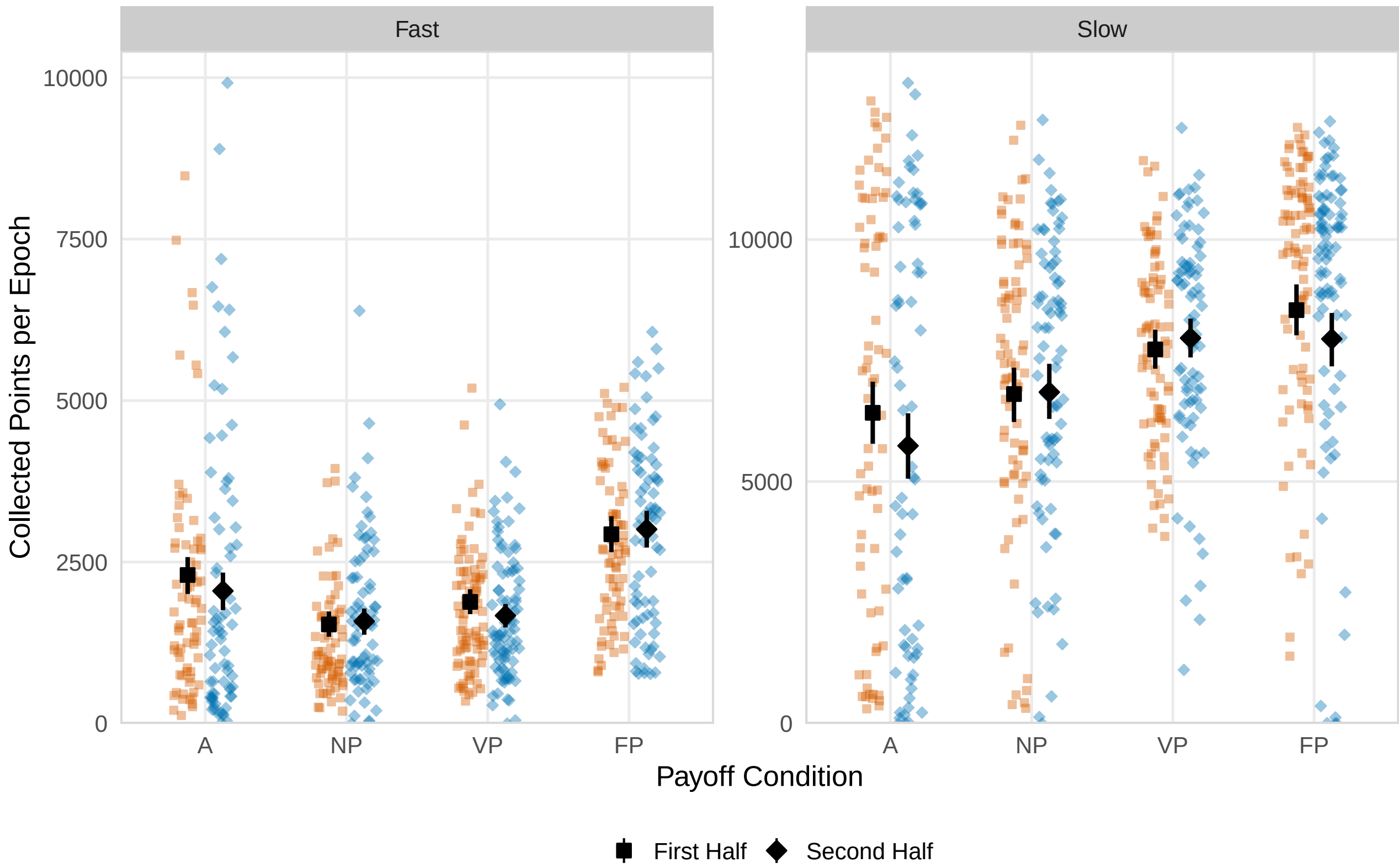

Figure 4: Collected Points: Temporal Contrast. Total number of collected points per treatment, shown for each payoff condition (A: Asocial Condition; NP: No Payoff-Sharing; VP: Voluntary Payoff-Sharing; FP: Full Payoff-Sharing) and resource speed (slow vs. fast), split by the first half (0–7.5 min) and second half (7.5–15 min) of the experimental session (shape and color). Each dot represents an individual participant. Larger black dots and error bars show posterior means and 90% HPDIs. The numerical values for the contrasts between the conditions are shown in the Table 4. Statistics are derived from $n = 621$ participants (Fast resource: A $n = 73$, NP $n = 72$, VP $n = 86$, FP $n = 74$; Slow resource: A $n = 71$, NP $n = 80$, VP $n = 79$, FP $n = 86$).

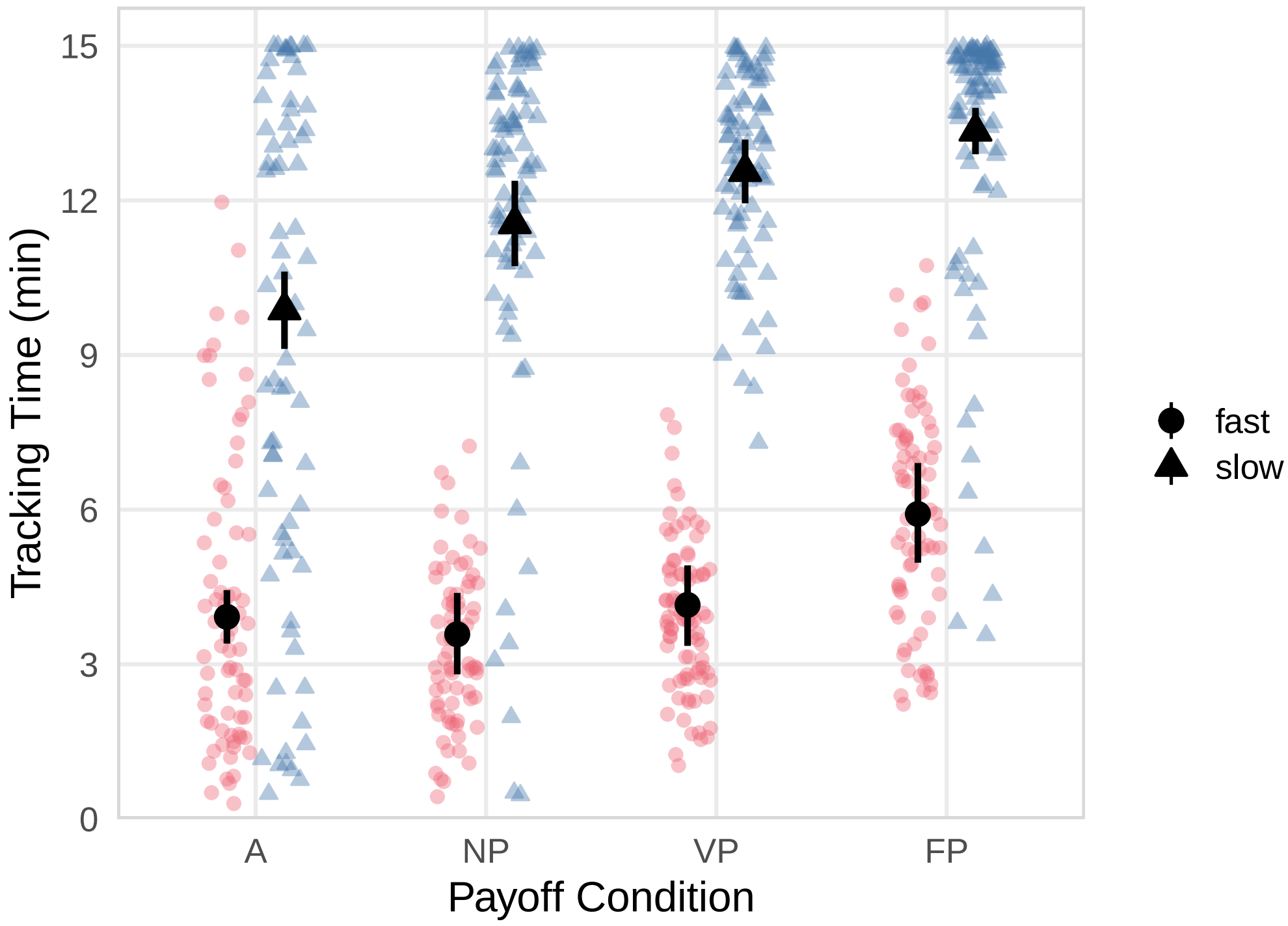

Figure 5: Tracking Time. Mean tracking time in minutes per treatment, shown for each payoff condition (A: Asocial Condition; NP: No Payoff-Sharing; VP: Voluntary Payoff-Sharing; FP: Full Payoff-Sharing) and resource speed (slow vs. fast). Each dot represents an individual participant. Larger black dots and error bars show posterior means and 90% HPDIs. The numerical values for the contrasts between the conditions are shown in the Table 6. Statistics are derived from $n = 621$ participants (Fast resource: A $n = 73$, NP $n = 72$, VP $n = 86$, FP $n = 74$; Slow resource: A $n = 71$, NP $n = 80$, VP $n = 79$, FP $n = 86$).

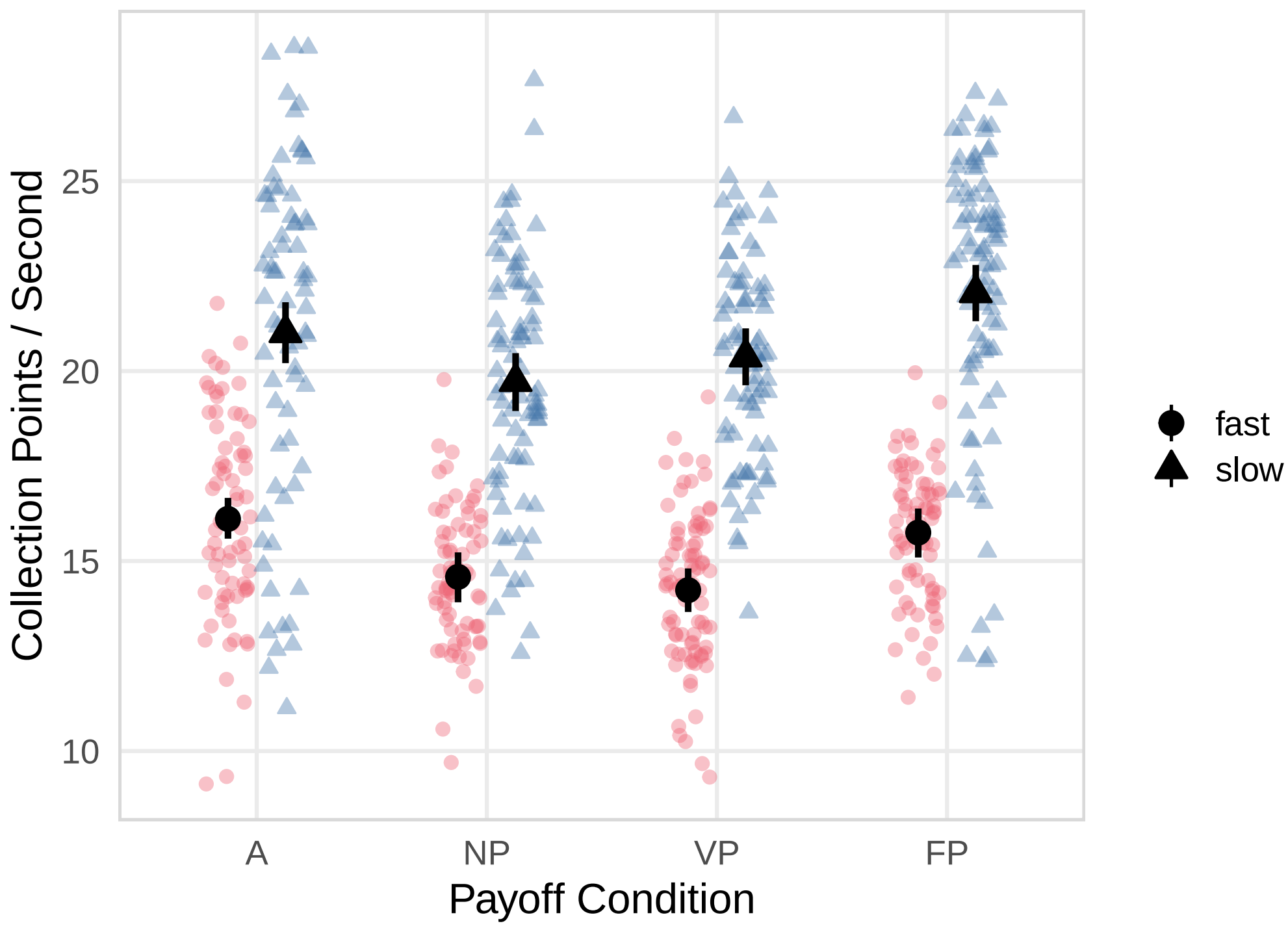

Figure 6: Tracking Efficiency. Mean tracking efficiency (i.e., average number of points earned per second while on the resource) per treatment, shown for each payoff condition (A: Asocial Condition; NP: No Payoff-Sharing; VP: Voluntary Payoff-Sharing; FP: Full Payoff-Sharing) and resource speed (slow vs. fast). Each dot represents an individual participant. Larger black dots and error bars show posterior means and 90% HPDIs. The numerical values for the contrasts between the conditions are shown in the Table 7. Statistics are derived from n = 621 participants (Fast resource: A n = 73, NP n = 72, VP n = 86, FP n = 74; Slow resource: A n = 71, NP n = 80, VP n = 79, FP n = 86).

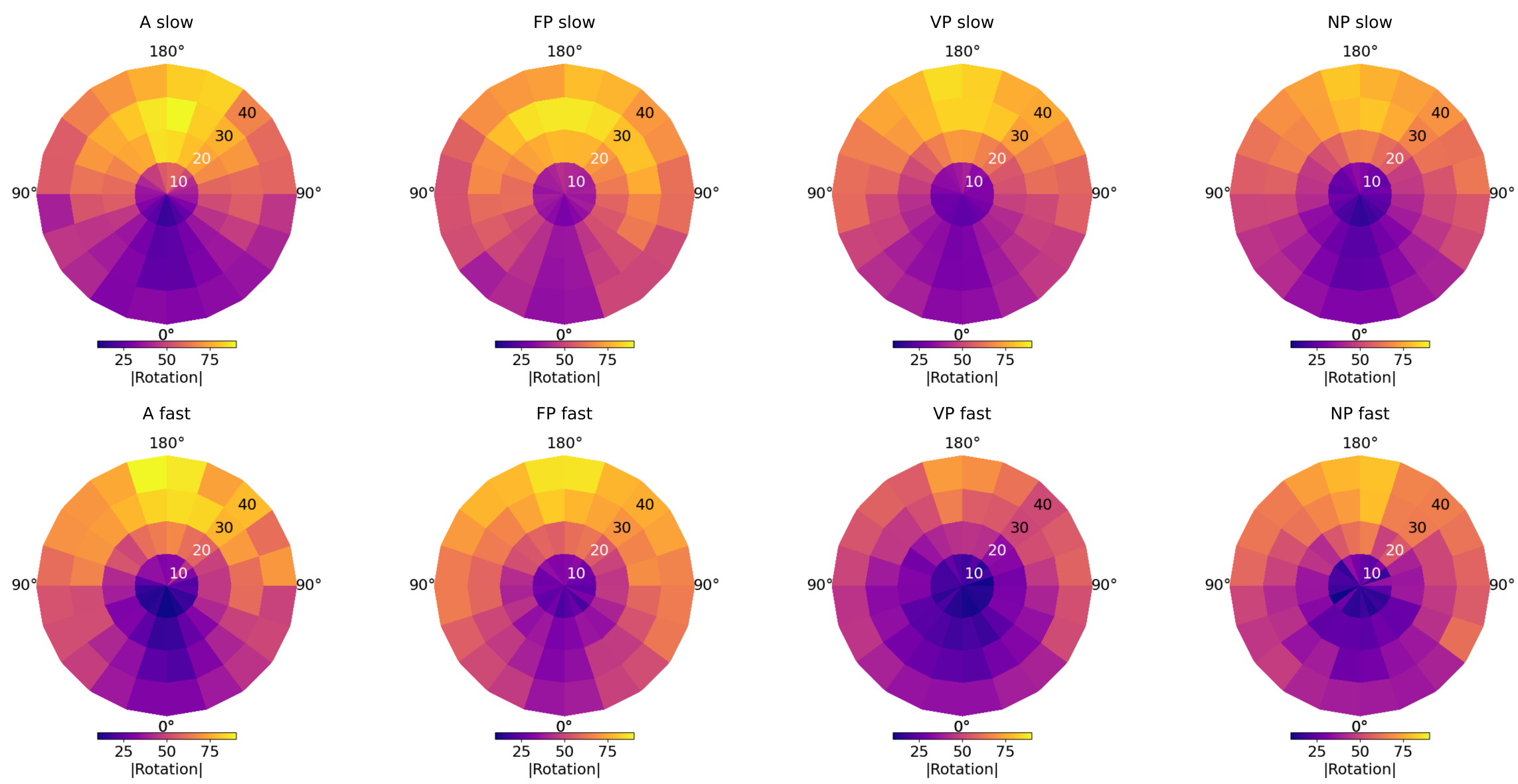

Figure 7: Participants' average rotation magnitude for different positions on the resource, shown for each payoff condition (A: Asocial Condition; NP: No Payoff-Sharing; VP: Voluntary Payoff-Sharing; FP: Full Payoff-Sharing) and resource speed (slow vs. fast).

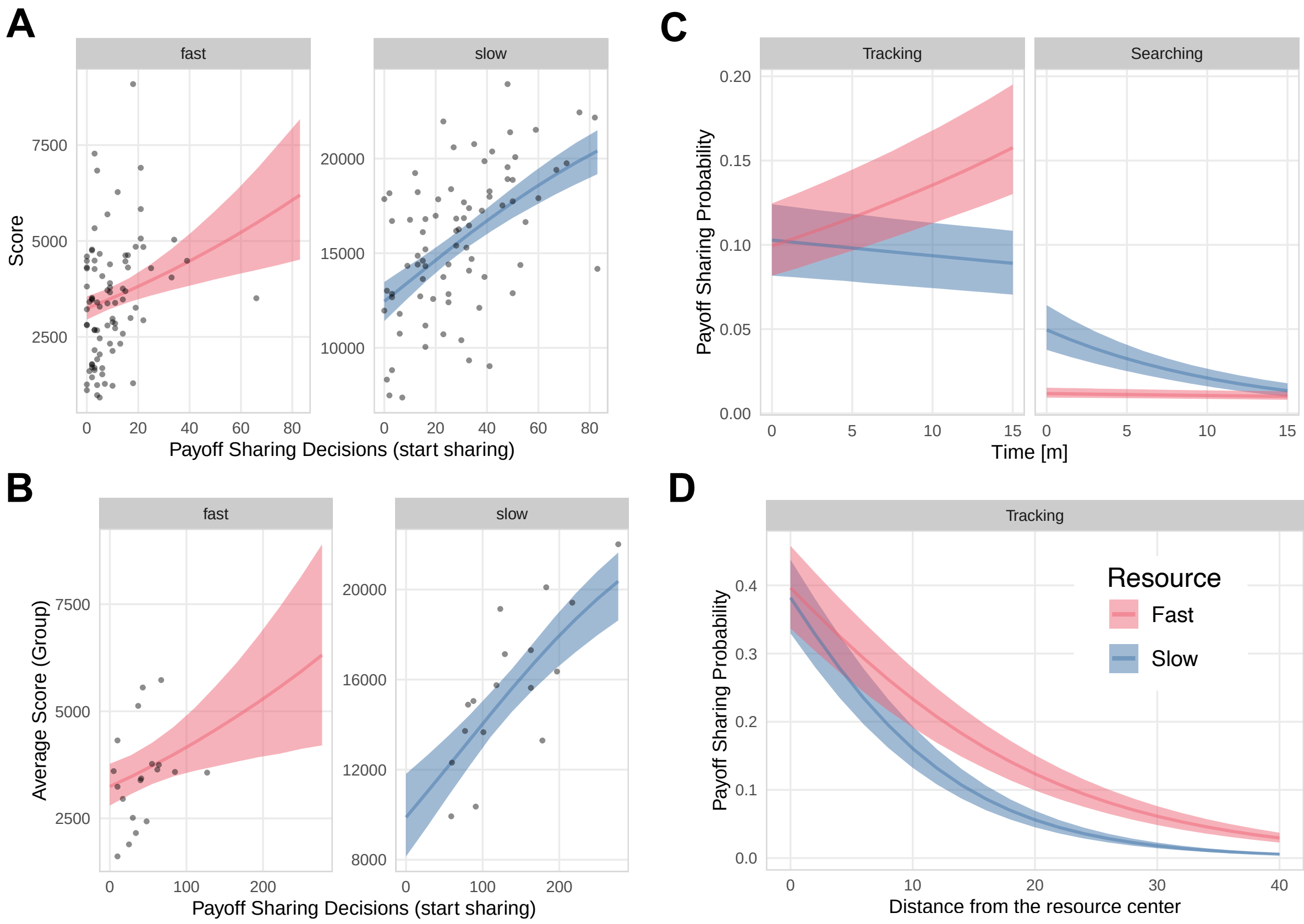

Figure 8: Voluntary Payoff-Sharing Condition. **A** Relationship between the number of individual payoff sharing decisions and final individual score; Each black dot represents an individual participant. **B** Relationship between the total number of sharing decisions within a group and the average group score; Each black dot represents a group. **C** Probability of sharing payoff information at each one-second time step, shown separately for searching and tracking states over the course of the experiment (x-axis). **D** Probability of sharing payoff information at each one-second time step, relative to the distance to the resource center (x-axis). The lines show the posterior predictive mean, and the shade areas the 90% HPDIs. Statistics are derived from $n = 165$ participants in the Voluntary Payoff-Sharing condition (Fast resource: $n = 86$; Slow resource: $n = 79$).

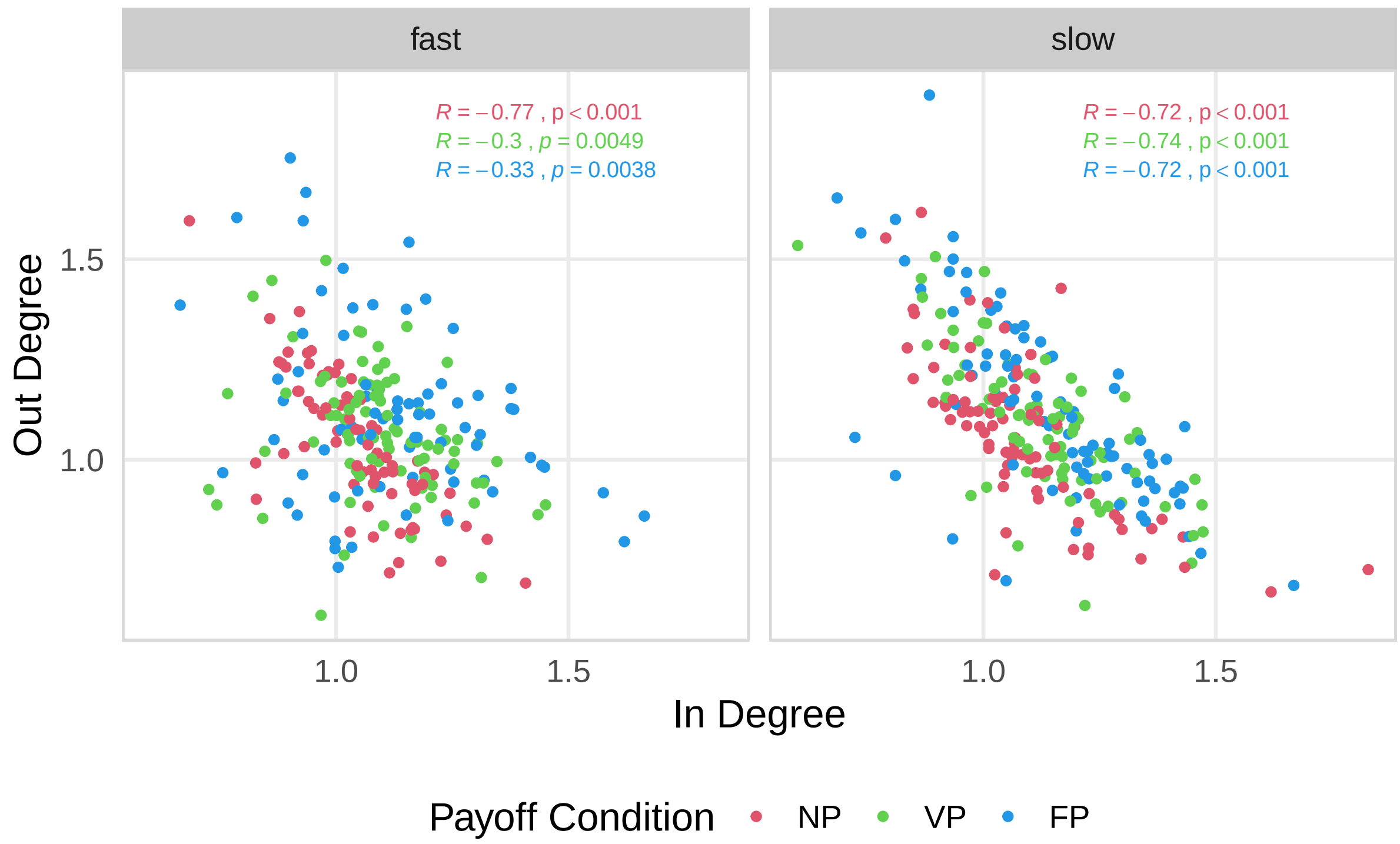

Figure 9: Relationship between In-Degree and Out-Degree for each Payoff Condition. Scatter plots show the mean in-degree and out-degree per participant, colored by payoff condition. NP: No Payoff-Sharing; VP: Voluntary Payoff-Sharing; FP: Full Payoff-Sharing. Separate panels correspond to different resource speed conditions. Pearson's correlation coefficient (r) and p-value are shown in each panel. Across all conditions, in-degree and out-degree were negatively correlated (Pearson's $r = -0.55$, 95% CI $[-0.61, -0.48]$, $p < 0.001$). Each dot represents one participant. Statistics are derived from $n = 477$ participants (Fast resource: NP $n = 72$, VP $n = 86$, FP $n = 74$; Slow resource: NP $n = 80$, VP $n = 79$, FP $n = 86$).

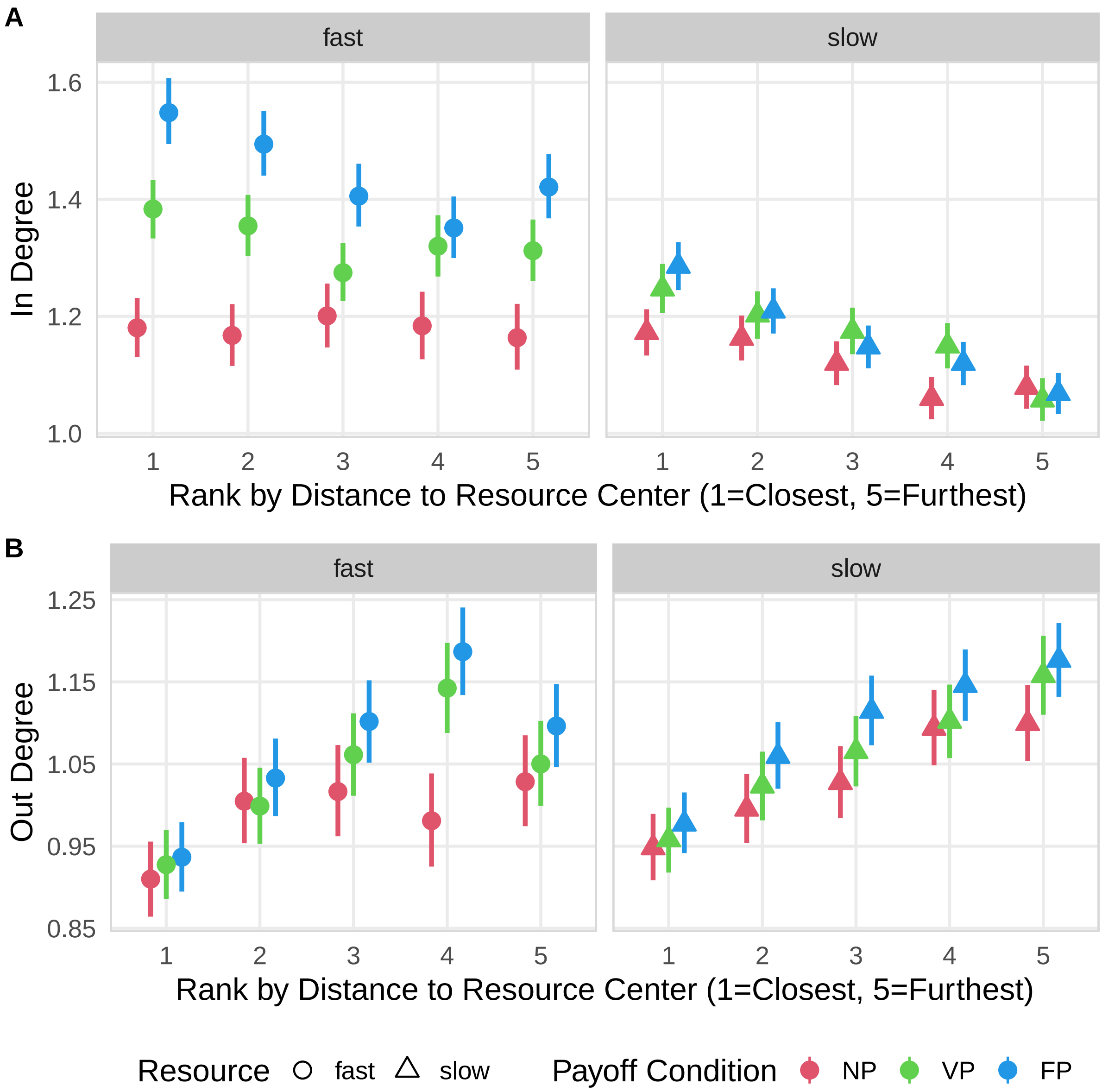

Figure 10: Participant's Distance Rank Relationship to Degree. Average in-degree (**A**) and out-degree (**B**) as a function of each participant's distance rank from the resource center within the group while tracking the resource (i.e., within detection range). Distance rank is assigned per group (1 = closest to resource center, 5 = furthest). Results are shown by payoff sharing condition (color NP: No Payoff-Sharing; VP: Voluntary Payoff-Sharing; FP: Full Payoff-Sharing) and by resource speed (columns). Dots and error bars show posterior means and 90% HPDIs. Statistics are derived from $n = 477$ participants (Fast resource: NP $n = 72$, VP $n = 86$, FP $n = 74$; Slow resource: NP $n = 80$, VP $n = 79$, FP $n = 86$).

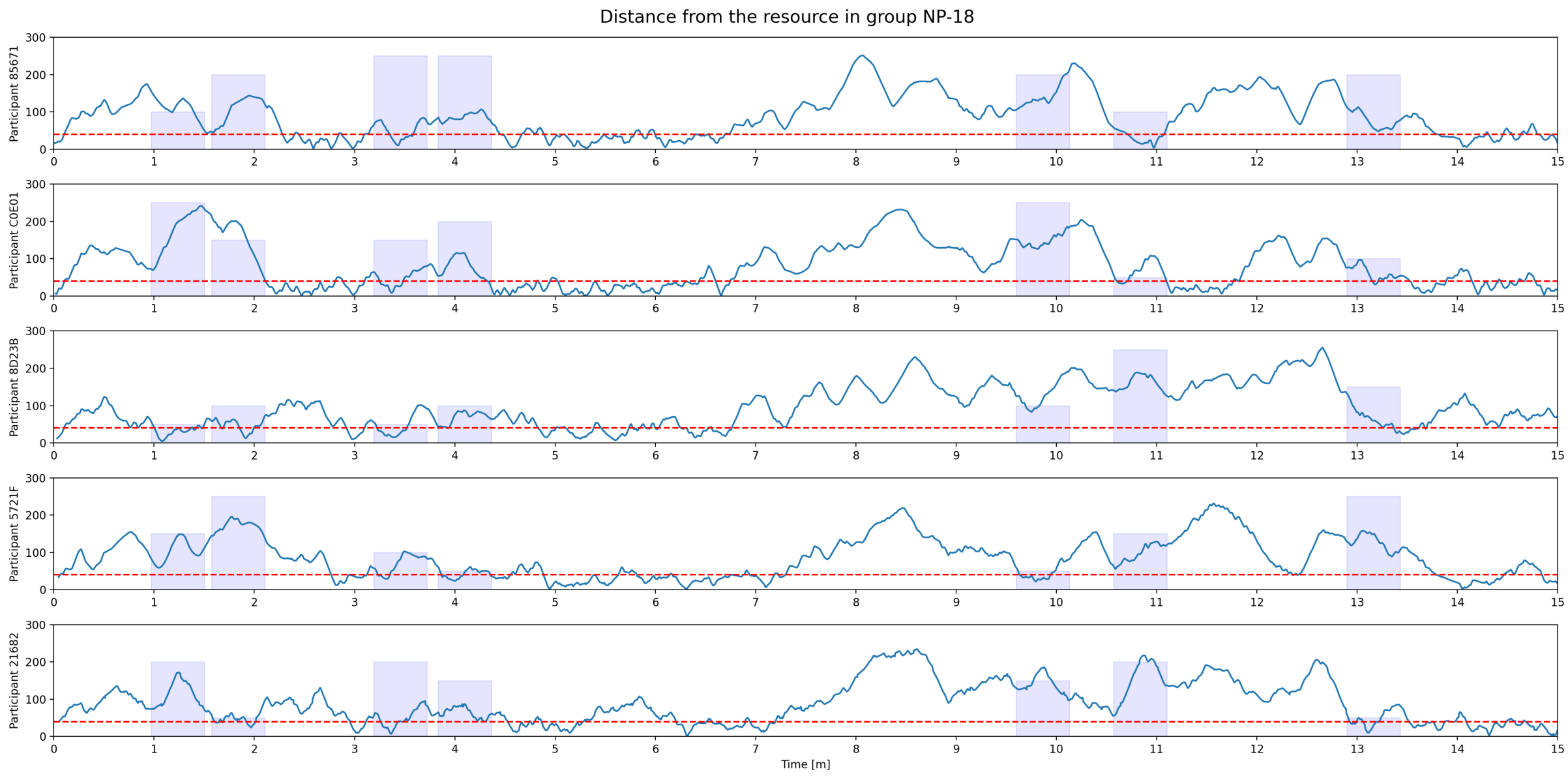

Figure 11: Illustration of resource discovery events. The solid line shows the time series of the distance from the resource for each participant, the dashed line shows the resource detection threshold. Transparent rectangles highlight the resource discovery events. The height of the rectangle indicates the order in which participants crossed the resource discovery area.

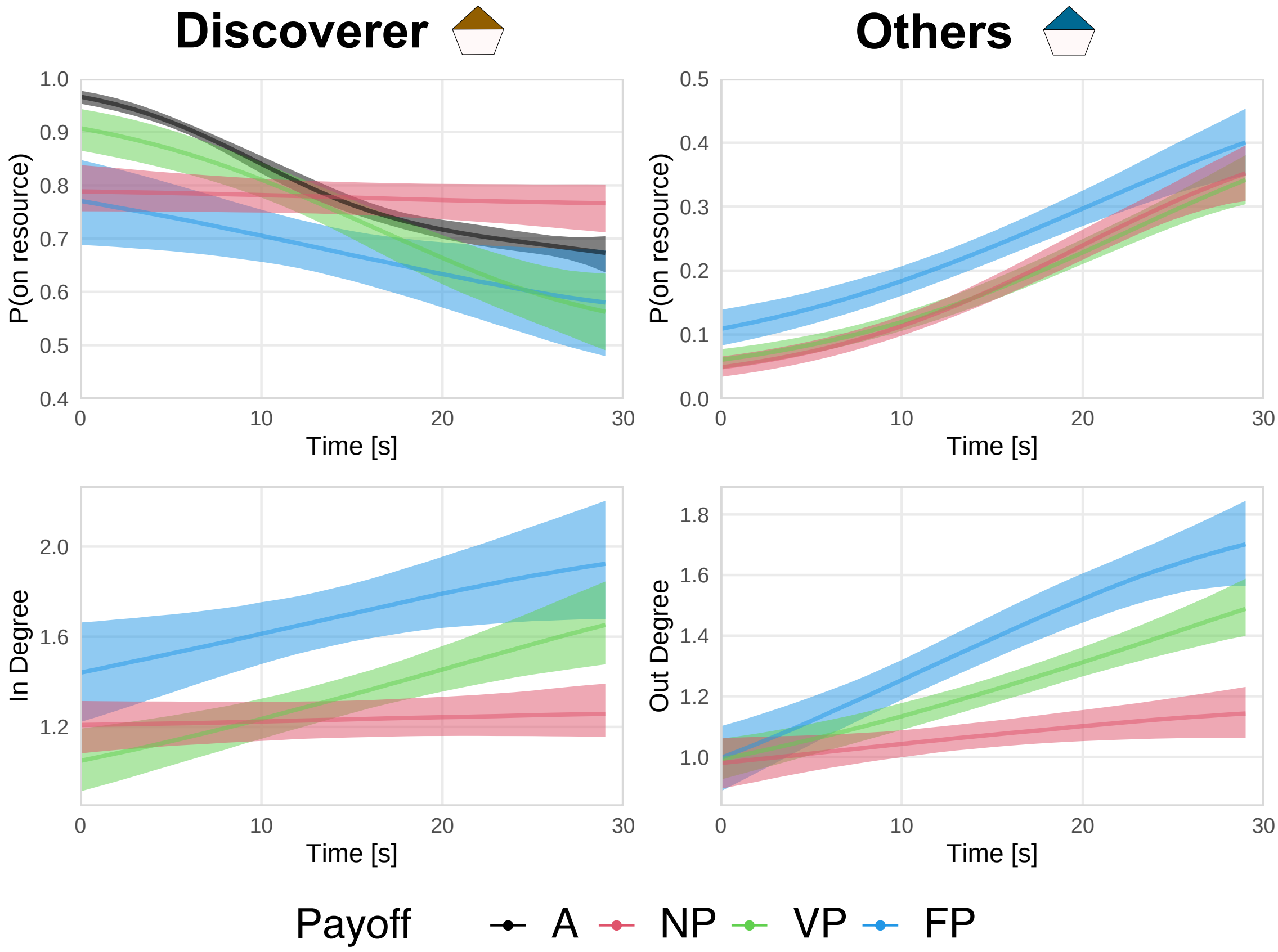

Figure 12: Resource discovery events (slow resource condition). top-left: Probability of remaining on the resource of the first discoverer. **top-right**: Average probability of other players in the group arriving at and staying on the resource. **bottom-left**: Average in-degree of the first player. **bottom-right**: Average out-degree of the other players. A: Asocial Condition; NP: No Payoff-Sharing; VP: Voluntary Payoff-Sharing; FP: Full Payoff-Sharing. Dark lines show posterior means of a Bayesian Gaussian process regression; shades represent 90% HPDIs. Statistics are derived from $n = 621$ participants (Fast resource: A $n = 73$, NP $n = 72$, VP $n = 86$, FP $n = 74$; Slow resource: A $n = 71$, NP $n = 80$, VP $n = 79$, FP $n = 86$).

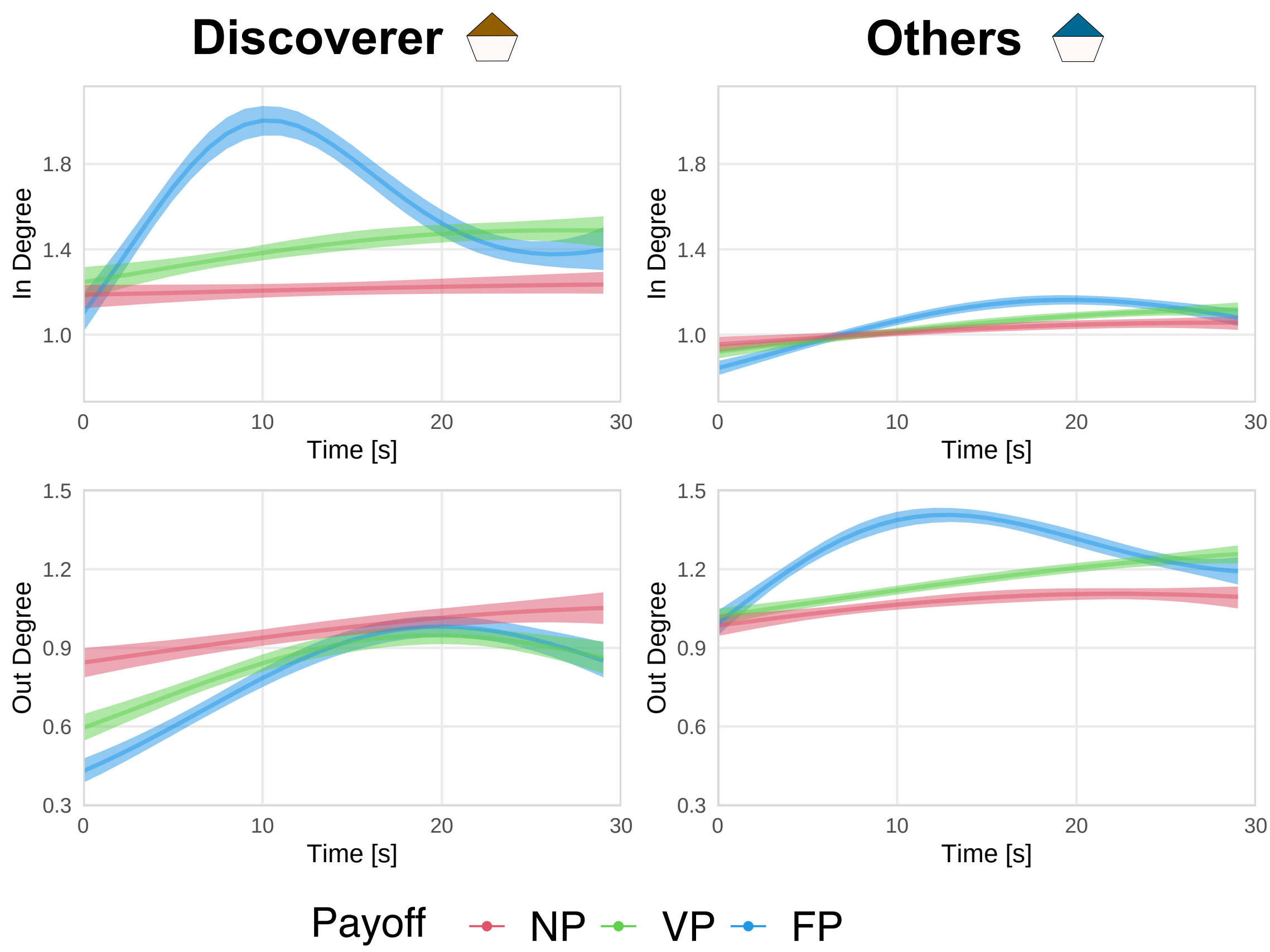

Figure 13: Resource discovery events (fast resource condition; in-/out-degree full). top-left: Average in-degree of the first player. **top-right**: Average in-degree of the other players. **bottom-left**: Average out-degree of the first player. **bottom-right**: Average out-degree of the other players. NP: No Payoff-Sharing; VP: Voluntary Payoff-Sharing; FP: Full Payoff-Sharing. Dark lines show posterior means of a Bayesian Gaussian process regression; shades represent 90% HPDIs. Statistics are derived from $n = 621$ participants (Fast resource: A $n = 73$, NP $n = 72$, VP $n = 86$, FP $n = 74$; Slow resource: A $n = 71$, NP $n = 80$, VP $n = 79$, FP $n = 86$).

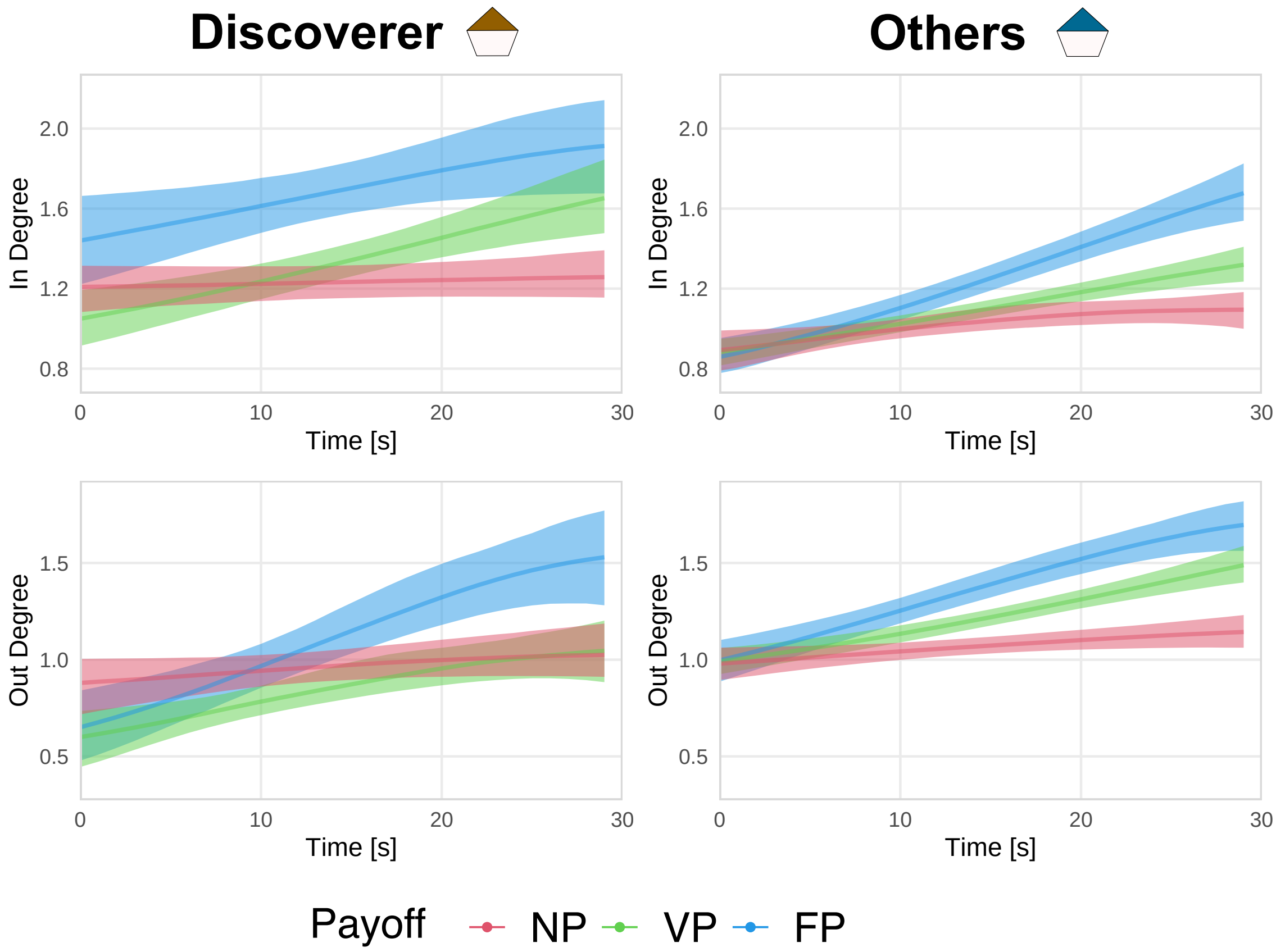

Figure 14: Resource discovery events (slow resource condition; in-/out-degree full). top-left: Average in-degree of the first player. **top-right**: Average in-degree of the other players. **bottom-left**: Average out-degree of the first player. **bottom-right**: Average out-degree of the other players. NP: No Payoff-Sharing; VP: Voluntary Payoff-Sharing; FP: Full Payoff-Sharing. Dark lines show posterior means of a Bayesian Gaussian process regression; shades represent 90% HPDIs. Statistics are derived from $n = 621$ participants (Fast resource: A $n = 73$, NP $n = 72$, VP $n = 86$, FP $n = 74$; Slow resource: A $n = 71$, NP $n = 80$, VP $n = 79$, FP $n = 86$).

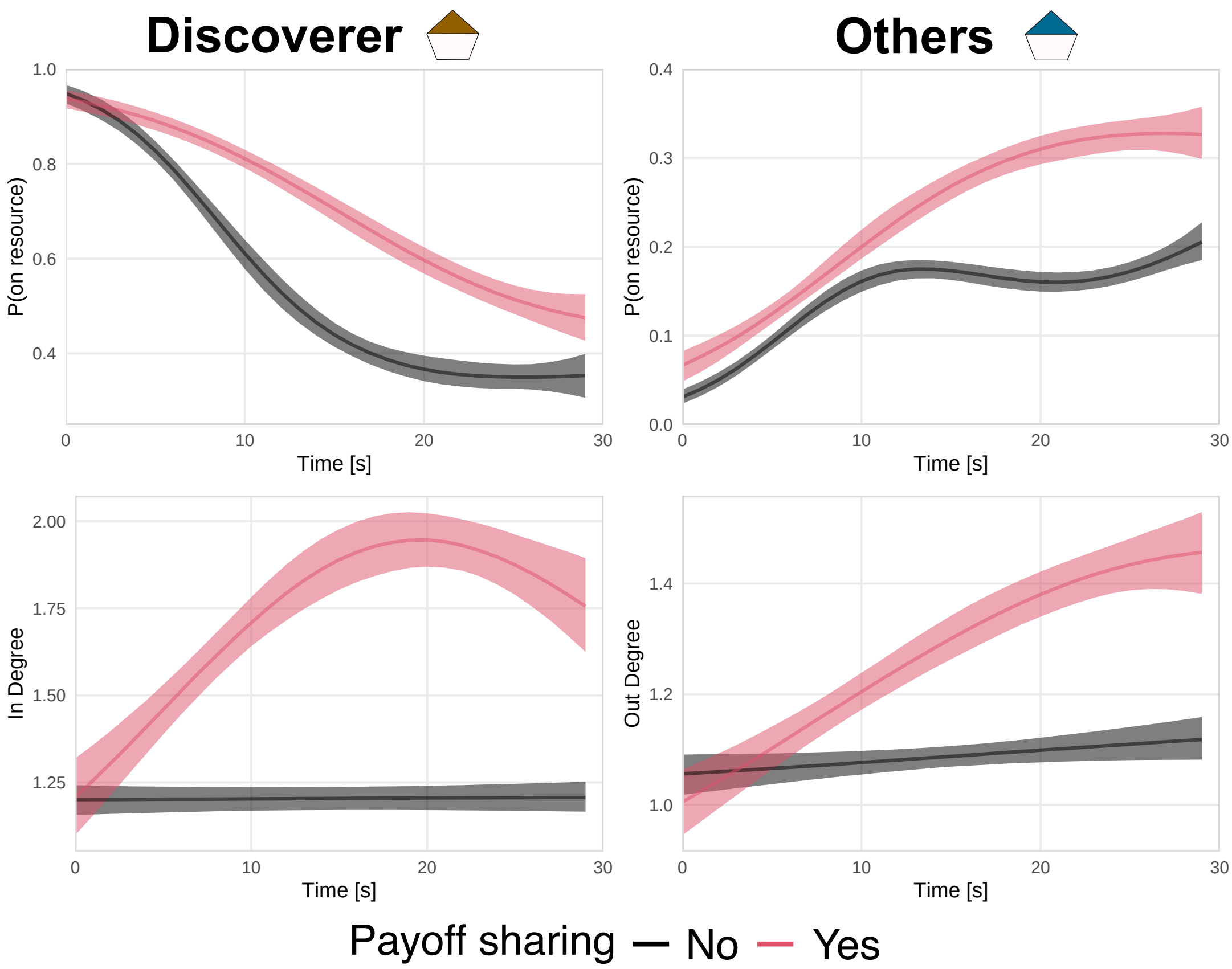

Figure 15: Resource discovery events in VP condition (fast resource condition). top: Schematic of a resource discovery event. **middle-left**: Probability of remaining on the resource of the first discoverer. **middle-right**: Average probability of other players in the group arriving at and staying on the resource. **bottom-left**: Average in-degree of the first player. **bottom-right**: Average out-degree of the other players. The color indicates whether the "discoverer" shared the payoff information for at least two seconds at any point after the event began. Dark lines show posterior means of a Bayesian Gaussian process regression; shades represent 90% HPDIs. Statistics are derived from $n = 621$ participants (Fast resource: A $n = 73$, NP $n = 72$, VP $n = 86$, FP $n = 74$; Slow resource: A $n = 71$, NP $n = 80$, VP $n = 79$, FP $n = 86$).

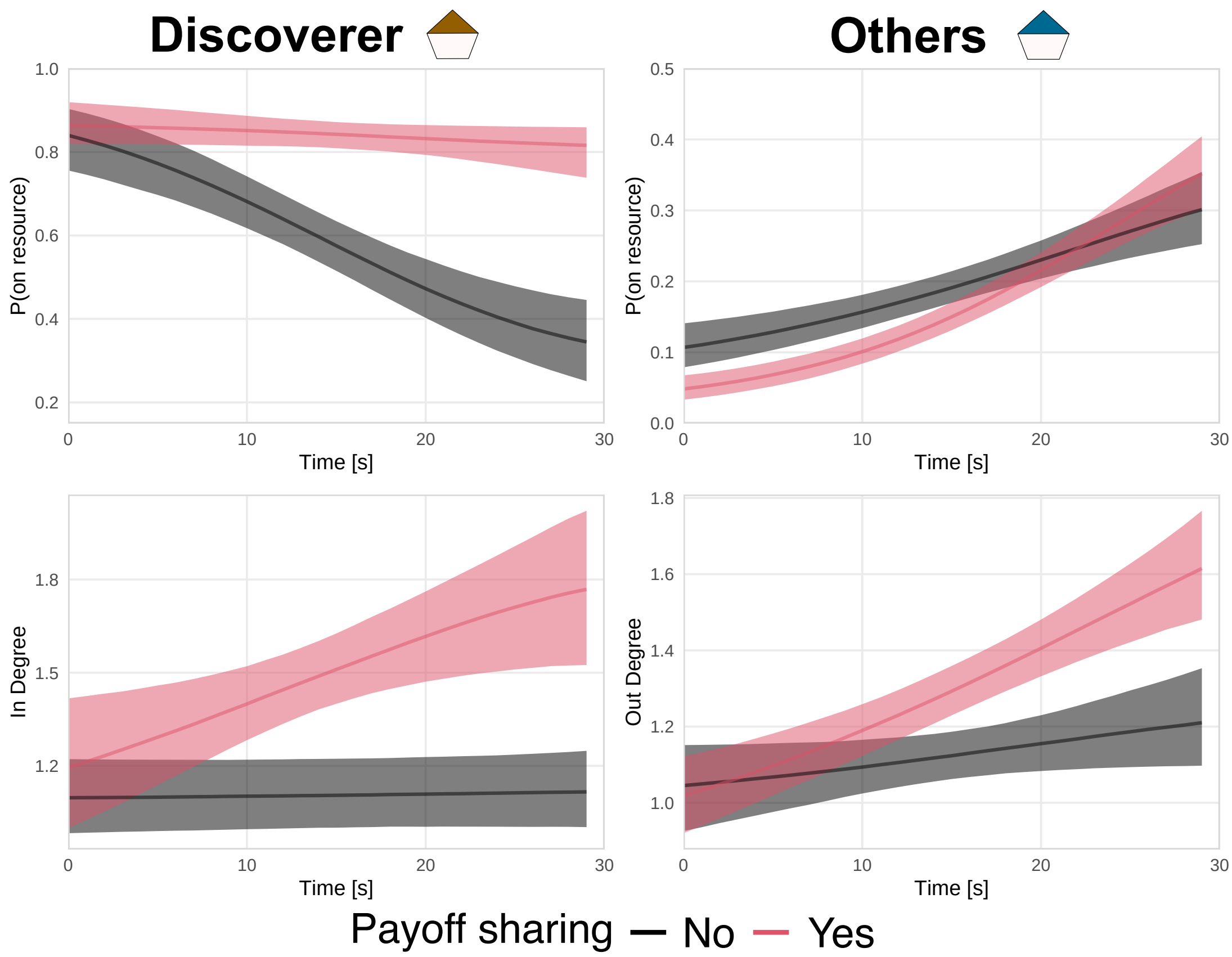

Figure 16: Resource discovery events in VP payoff sharing condition (slow resource condition). top: Schematic of a resource discovery event. **middle-left**: Probability of remaining on the resource of the first discoverer. **middle-right**: Average probability of other players in the group arriving at and staying on the resource. **bottom-left**: Average in-degree of the first player. **bottom-right**: Average out-degree of the other players. The color indicates whether the "discoverer" shared the payoff information for at least two seconds at any point after the event began. Dark lines show posterior means of a Bayesian Gaussian process regression; shades represent 90% HPDIs. Statistics are derived from $n = 621$ participants (Fast resource: A $n = 73$, NP $n = 72$, VP $n = 86$, FP $n = 74$; Slow resource: A $n = 71$, NP $n = 80$, VP $n = 79$, FP $n = 86$).

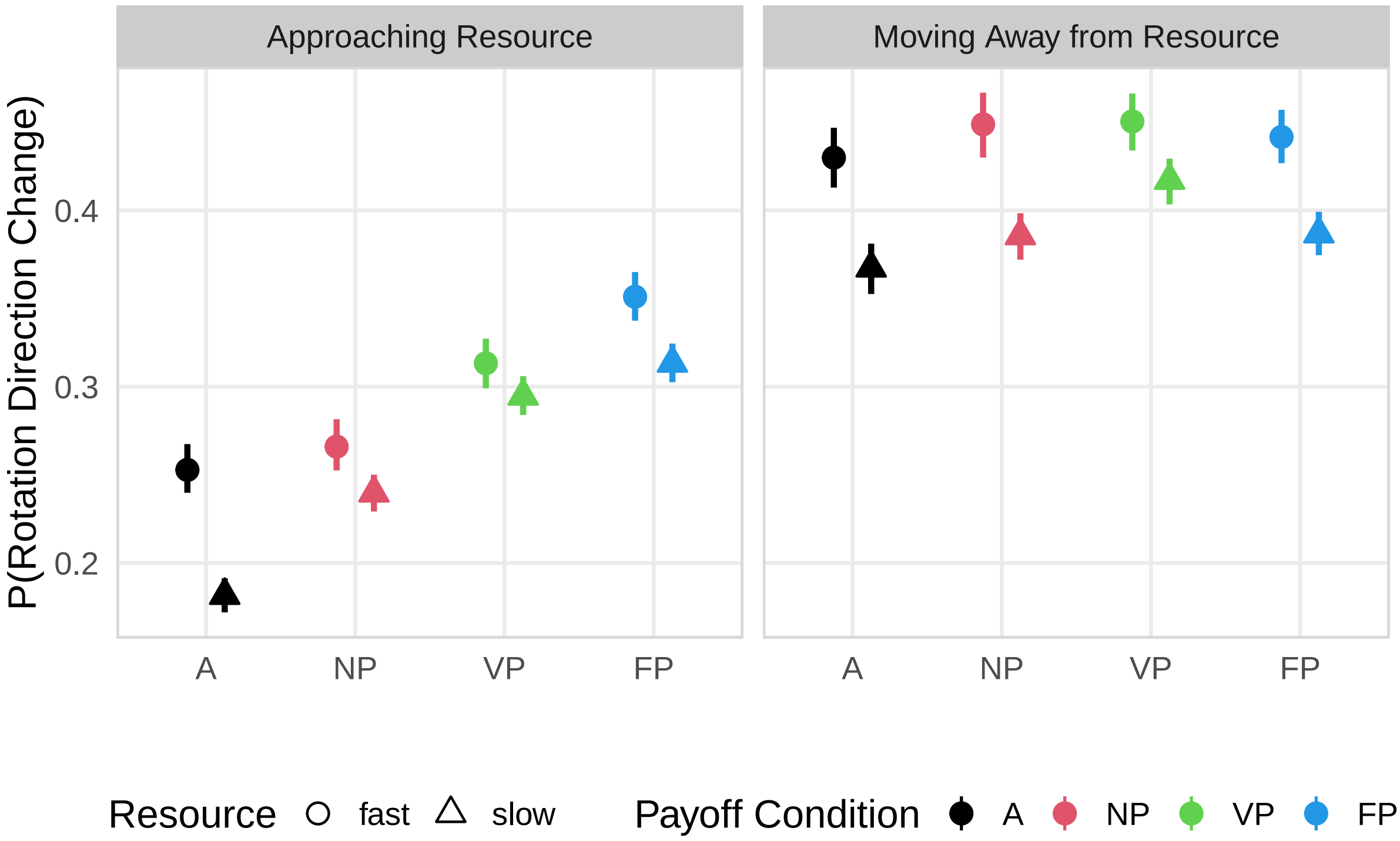

Figure 17: The probability of a rotation direction change as a response to a detector change. The columns show the state of the private information (detector change): the focal agent is approaching (**left panel**) or moving away from the resource center (**right panel**). Color (and the x-axis) denotes the payoff condition (NP: No Payoff-Sharing; VP: Voluntary Payoff-Sharing; FP: Full Payoff-Sharing), and point shape distinguishes the resource speed (slow vs. fast). Dots and error bars show posterior means and 90% HPDIs. Statistics are derived from $n = 621$ participants (Fast resource: A $n = 73$, NP $n = 72$, VP $n = 86$, FP $n = 74$; Slow resource: A $n = 71$, NP $n = 80$, VP $n = 79$, FP $n = 86$).

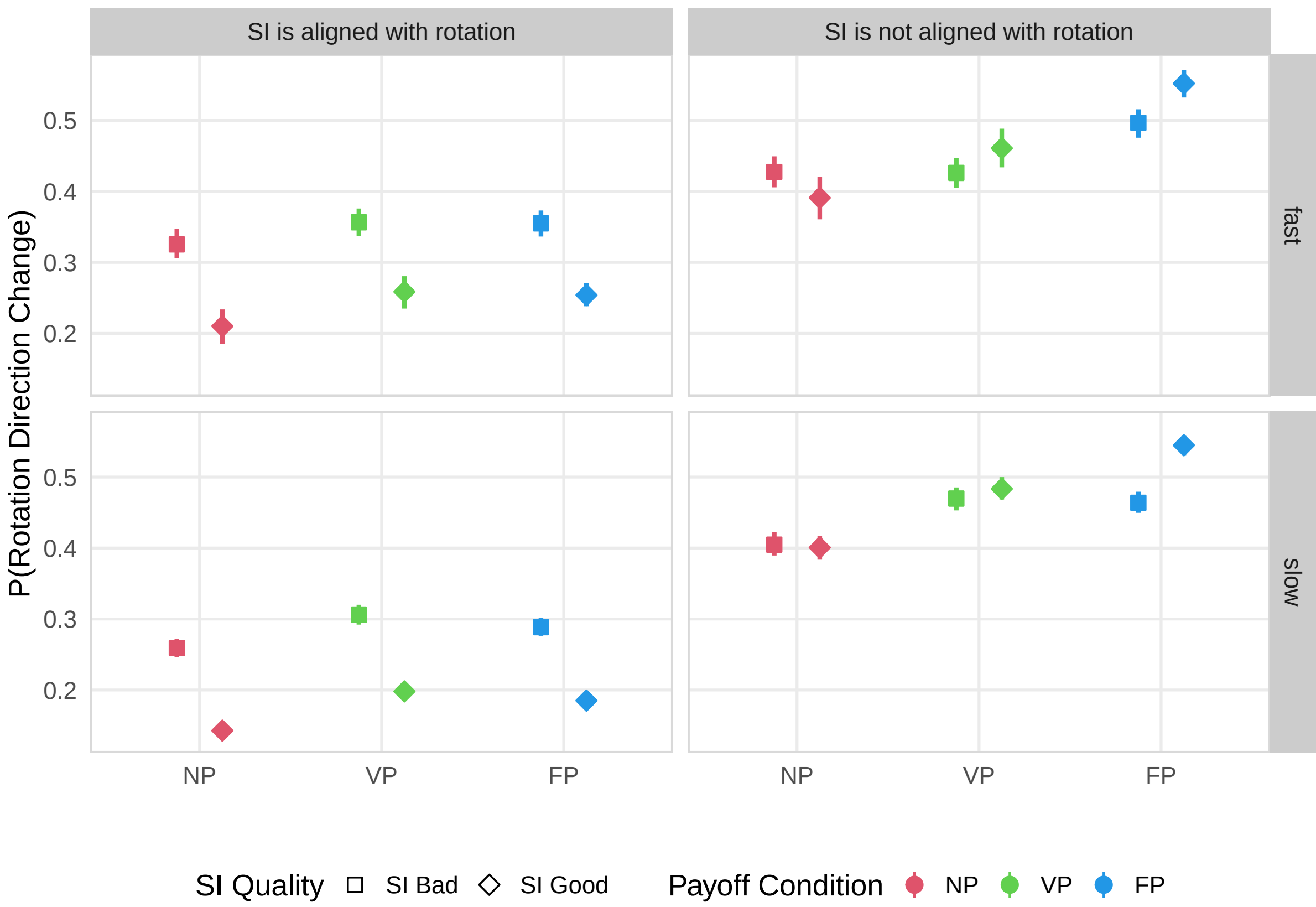

Figure 18: The probability of a change in rotation direction in response to social information (SI) alignment and quality. The columns show the social information state: social information alignment at $t-1$ is aligned (**left panel**) or misaligned (**right panel**) with the preceding rotation direction. The rows indicate resource speed (slow vs. fast). Color (and the x-axis) denotes the payoff condition (NP: No Payoff-Sharing; VP: Voluntary Payoff-Sharing; FP: Full Payoff-Sharing), and point shape indicates social information quality (high=Good vs. low=Bad). Dots and error bars show posterior means and 90% HPDIs. Statistics are derived from $n = 477$ participants (Fast resource: NP $n = 72$, VP $n = 86$, FP $n = 74$; Slow resource: NP $n = 80$, VP $n = 79$, FP $n = 86$).

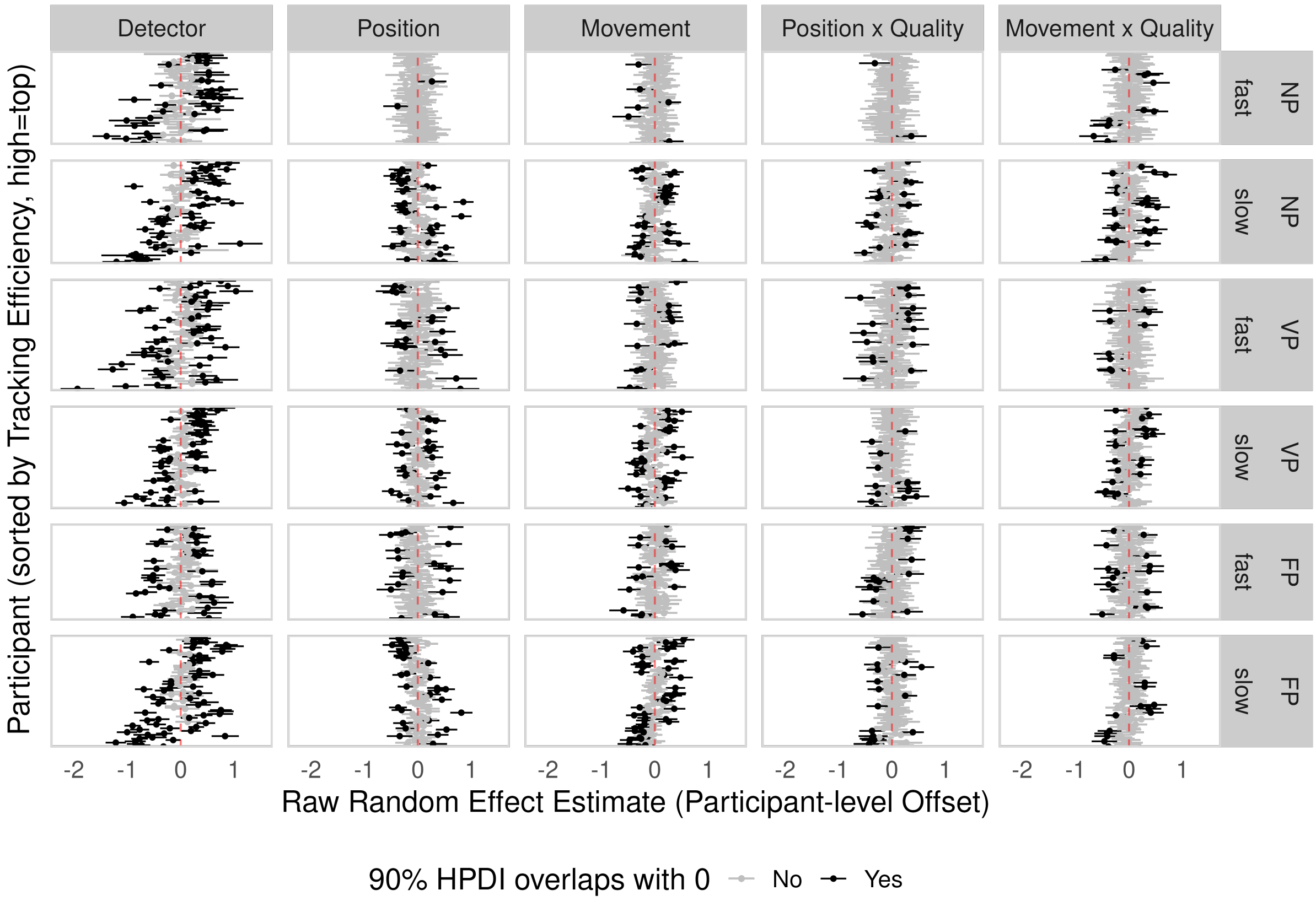

Figure 19: Individual variability in tracking strategies. Posterior estimates of participant-specific random effects extracted from the computational model of rotation magnitude during resource tracking. Each row within a panel represents a single participant's specific weighting of a private feature (Detector) or social feature (Position, Movement, Position x Quality, Movement x Quality). Points represent posterior means and horizontal lines denote 90% HPDIs. Within each experimental condition, participants are sorted on the y-axis by their empirical tracking efficiency, with the highest-performing individuals plotted at the top and the lowest-performing at the bottom of each panel. Colors indicates whether the 90% HPDI excludes (black) or overlaps (gray) with zero. Results are faceted by group payoff-sharing conditions (NP: No Payoff-Sharing; VP: Voluntary Payoff-Sharing; FP: Full Payoff-Sharing) and resource speed (fast, slow). Statistics are derived from n = 477 participants (Fast resource: NP n = 72, VP n = 86, FP n = 74; Slow resource: NP n = 80, VP n = 79, FP n = 86).

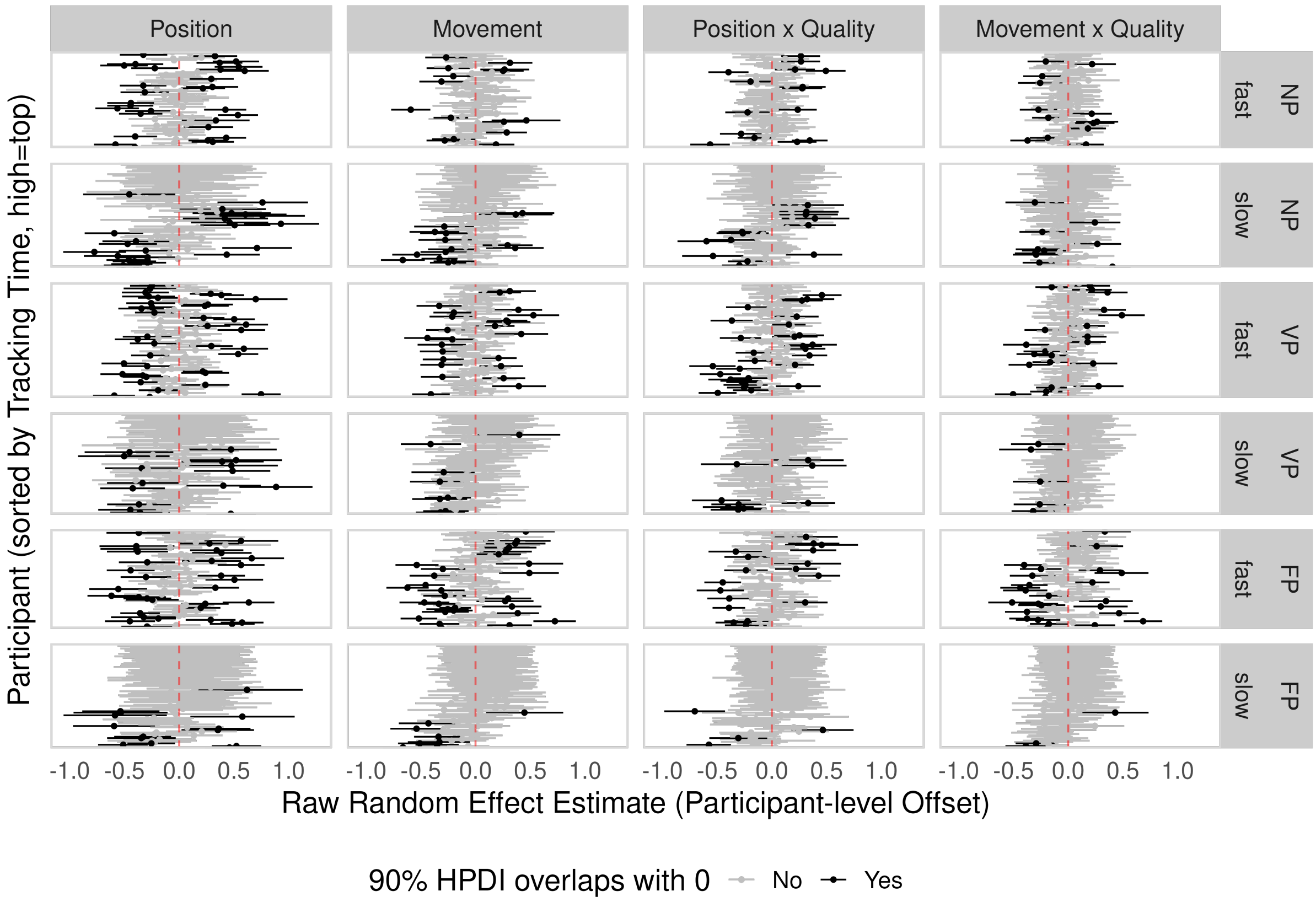

Figure 20: Individual variability in search strategies. Posterior estimates of participant-specific random effects extracted from the computational model of rotation magnitude during resource search. Each row within a panel represents a single participant's specific weighting of social feature (Position, Movement, Position x Quality, Movement x Quality). Points represent posterior means and horizontal lines denote 90% HPDIs. Within each experimental condition, participants are sorted on the y-axis by their empirical tracking time, with the highest-performing individuals plotted at the top and the lowest-performing at the bottom of each panel. Colors indicates whether the 90% HPDI excludes (black) or overlaps (gray) with zero. Results are faceted by group payoff-sharing conditions (NP: No Payoff-Sharing; VP: Voluntary Payoff-Sharing; FP: Full Payoff-Sharing) and resource speed (fast, slow). Statistics are derived from $n = 477$ participants (Fast resource: NP $n = 72$, VP $n = 86$, FP $n = 74$; Slow resource: NP $n = 80$, VP $n = 79$, FP $n = 86$).

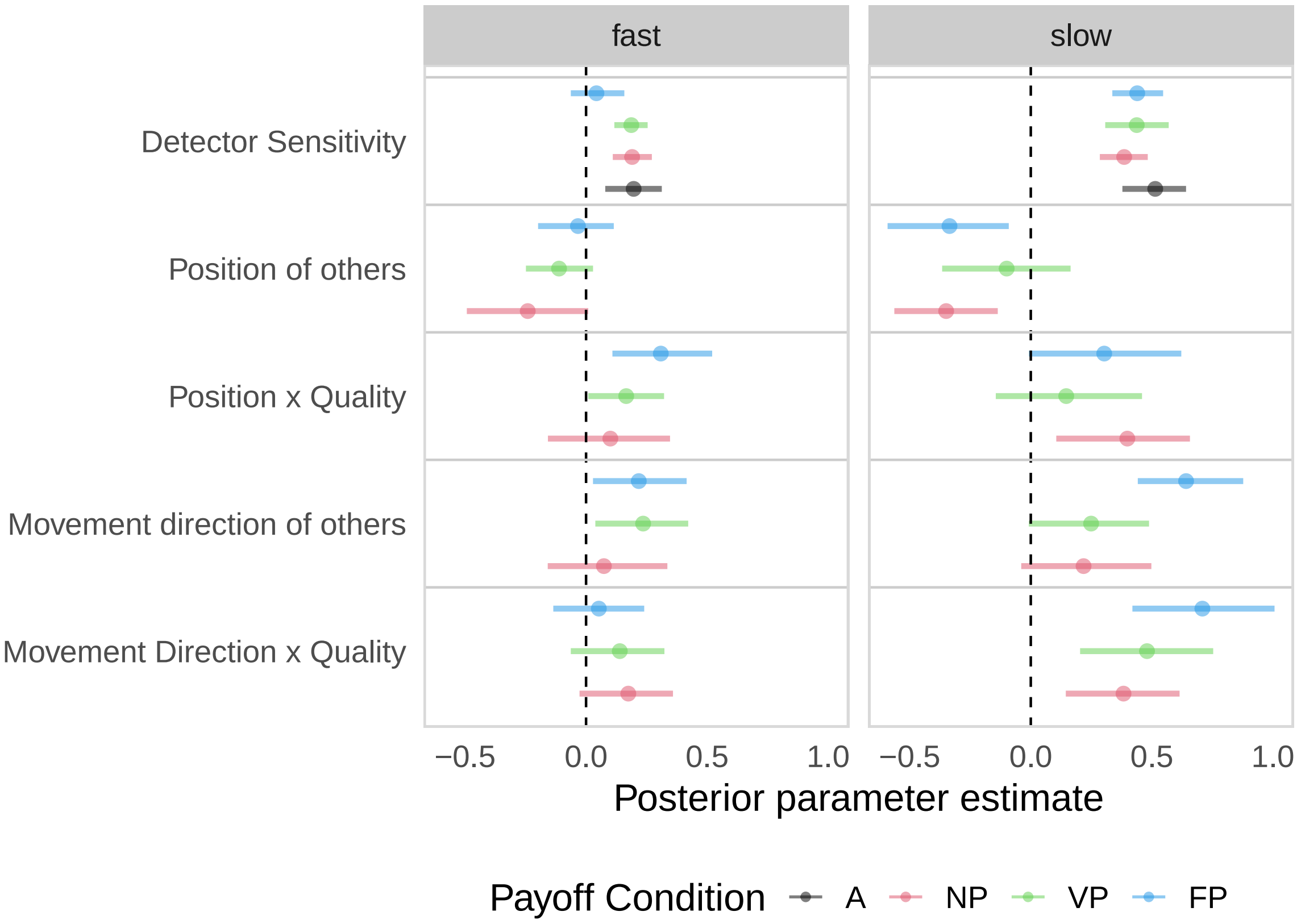

Figure 21: Posterior estimates of the relationship between individual decision weights and tracking efficiency. Individual decision weights are computed in the rotation magnitude model of tracking. Separate models were fit for each feature type (y-axis) and included interactions between payoff sharing condition (color) and resource speed (column). A: Alone Condition; NP: No Payoff-Sharing; VP: Voluntary Payoff-Sharing; FP: Full Payoff-Sharing. Dots and error bars show posterior means and 90% HPDIs. Statistics are derived from $n = 621$ participants (Fast resource: A $n = 73$, NP $n = 72$, VP $n = 86$, FP $n = 74$; Slow resource: A $n = 71$, NP $n = 80$, VP $n = 79$, FP $n = 86$).

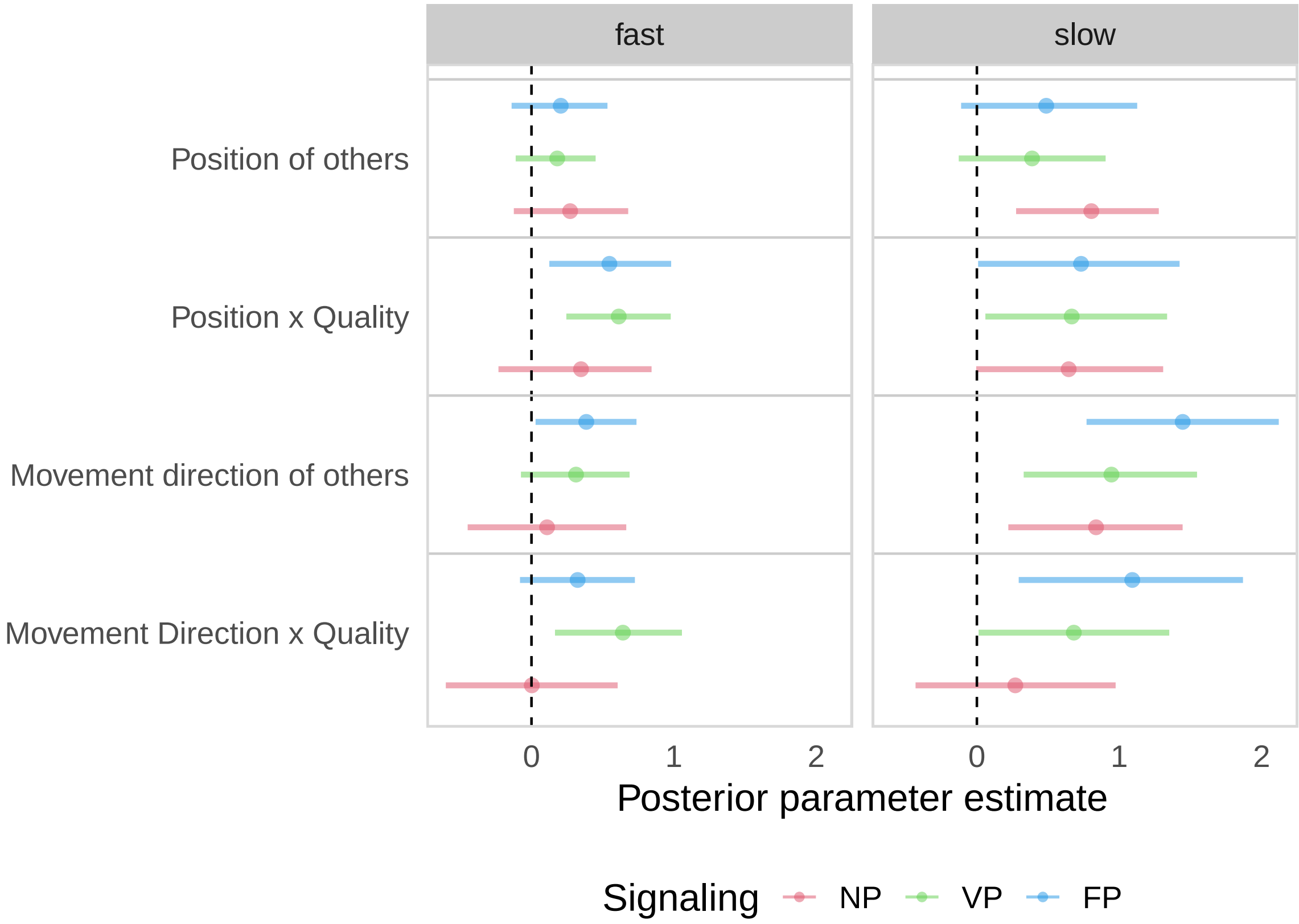

Figure 22: Posterior estimates of the relationship between individual decision weights and tracking time. Individual decision weights are computed in the rotation magnitude model of searching. Separate models were fit for each feature type (y-axis) and included interactions between payoff sharing condition (color) and resource speed (column). A: Alone Condition; NP: No Payoff-Sharing; VP: Voluntary Payoff-Sharing; FP: Full Payoff-Sharing. Dots and error bars show posterior means and 90% HPDIs. Statistics are derived from $n = 477$ participants (Fast resource: NP $n = 72$, VP $n = 86$, FP $n = 74$; Slow resource: NP $n = 80$, VP $n = 79$, FP $n = 86$).

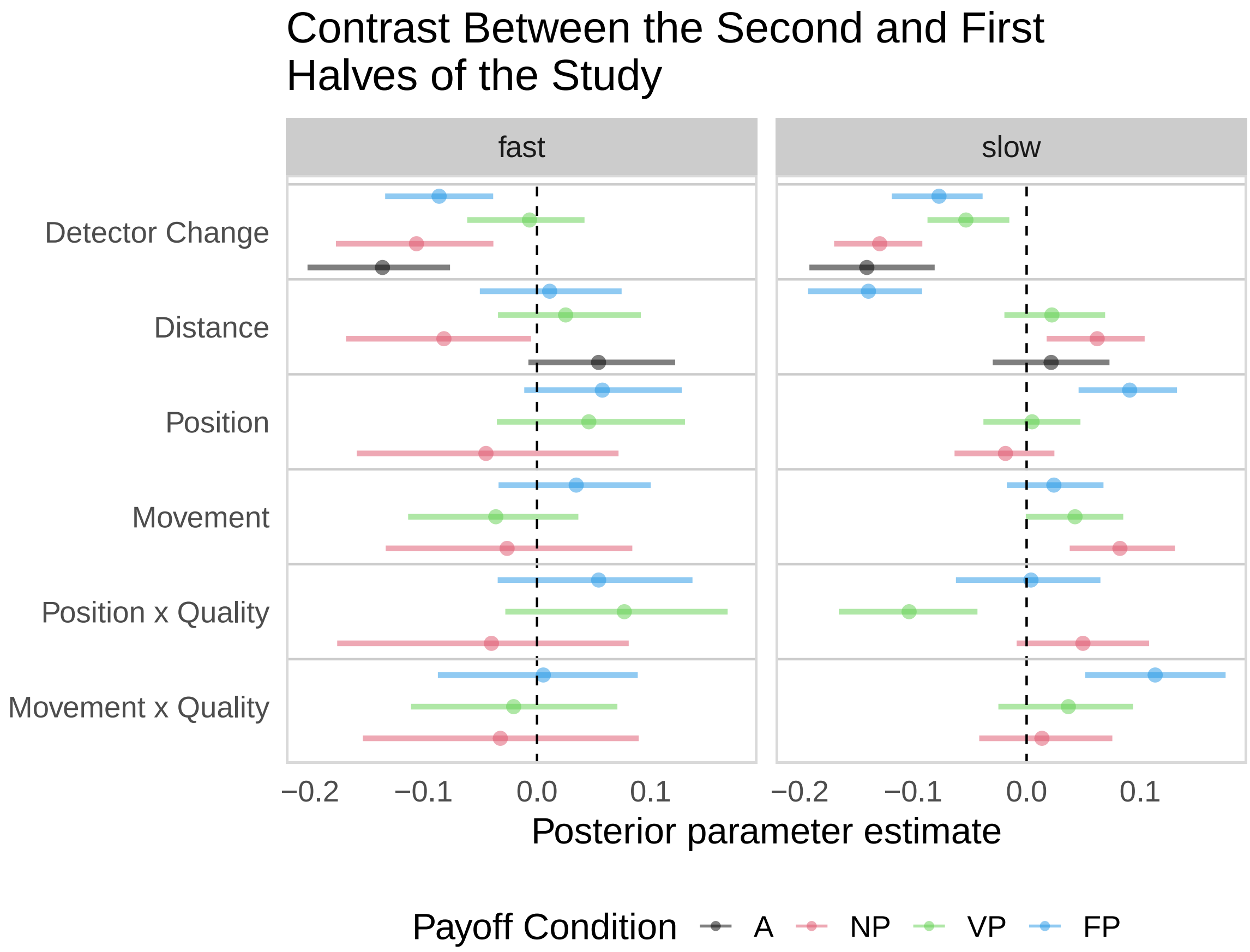

Figure 23: Temporal dynamics of computational decision weights during resource tracking. Posterior estimates of private/social feature weights in the computational model of rotation magnitude, split by the first half (0–7.5 min) and second half (7.5–15 min) of the experimental session. Results are shown across payoff-sharing conditions and resource speeds. A: Alone Condition; NP: No Payoff-Sharing; VP: Voluntary Payoff-Sharing; FP: Full Payoff-Sharing. Dots and error bars show posterior means and 90% HPDIs. The numerical values are shown in Table 13. Note that these visualizations show only a relevant subset of parameters from the full computational model. Statistics are derived from $n = 621$ participants (Fast resource: A $n = 73$, NP $n = 72$, VP $n = 86$, FP $n = 74$; Slow resource: A $n = 71$, NP $n = 80$, VP $n = 79$, FP $n = 86$).

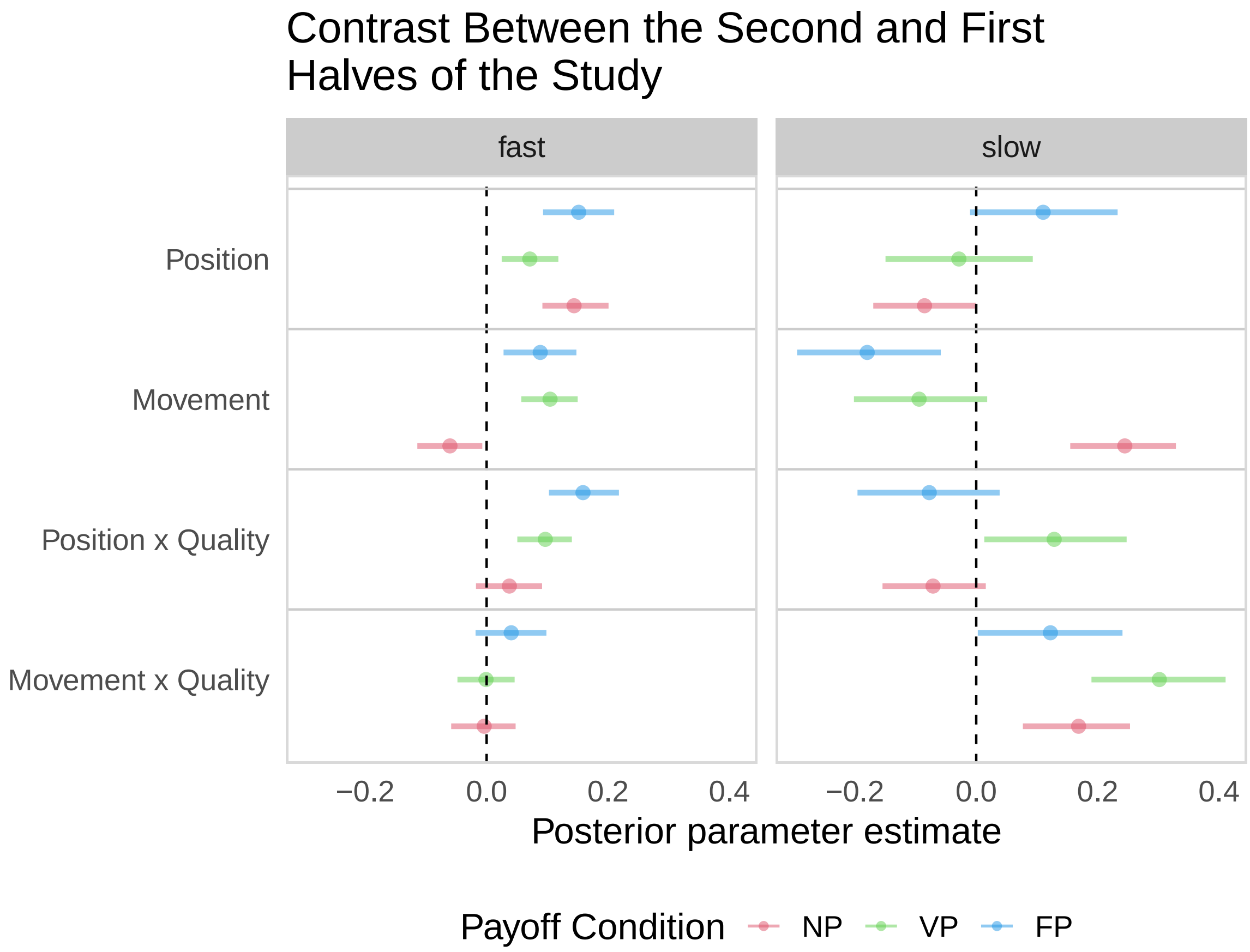

Figure 24: Temporal dynamics of computational decision weights during resource search. Posterior estimates of social feature weights in the computational model of rotation magnitude, split by the first half (0–7.5 min) and second half (7.5–15 min) of the experimental session. Results are shown across payoff-sharing conditions and resource speeds. NP: No Payoff-Sharing; VP: Voluntary Payoff-Sharing; FP: Full Payoff-Sharing. Dots and error bars show posterior means and 90% HPDIs. The numerical values are shown in Table 14. Note that these visualizations show only a relevant subset of parameters from the full computational model. Statistics are derived from n = 477 participants (Fast resource: NP n = 72, VP n = 86, FP n = 74; Slow resource: NP n = 80, VP n = 79, FP n = 86).

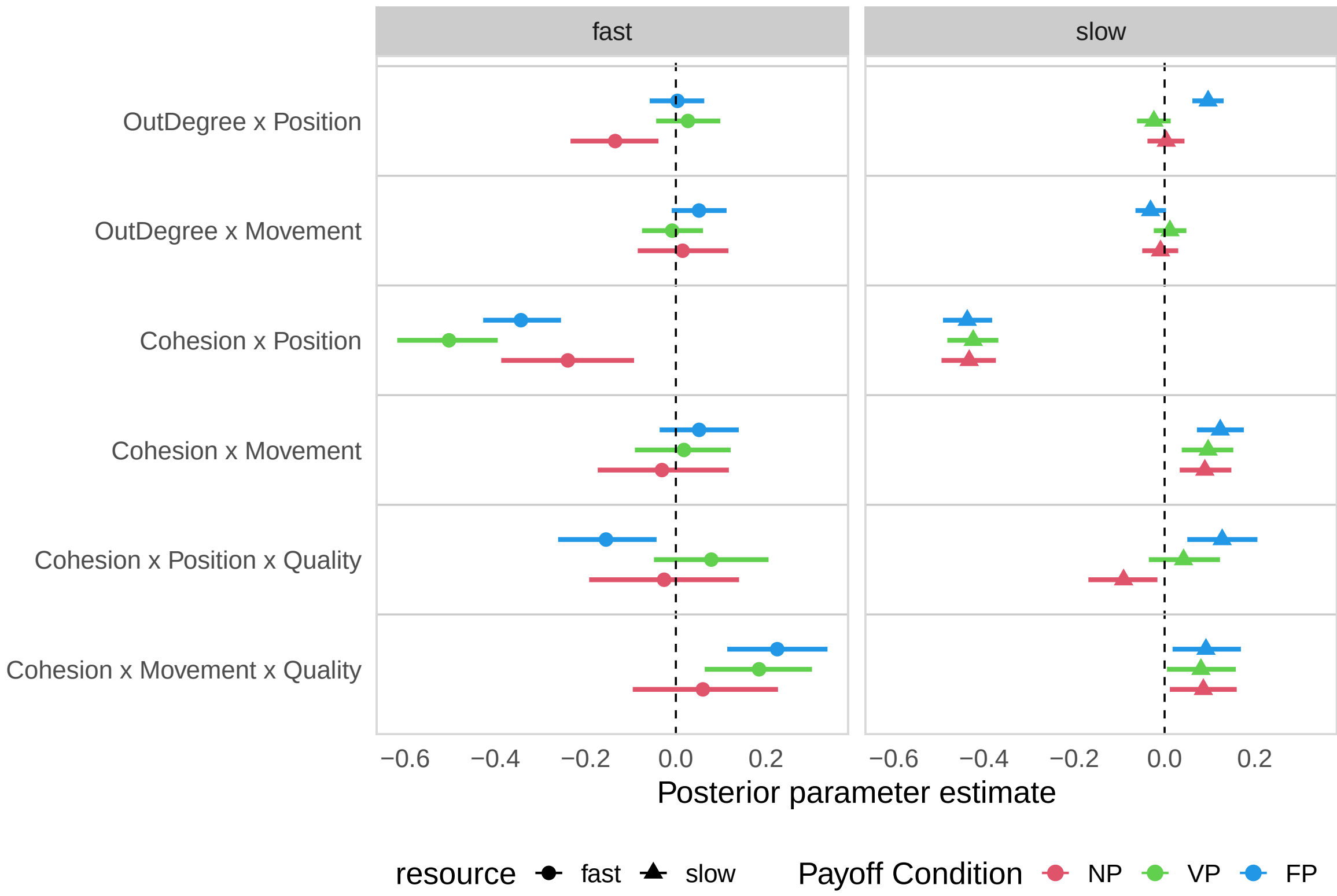

Figure 25: Impact of collective structural features on rotation decisions during tracking. The model was fitted to tracking states where participants observed two or more peers. High movement cohesion consistently devalues reliance on the group's spatial position (negative Cohesion x Position). Results are shown across payoff-sharing conditions and resource speeds. NP: No Payoff-Sharing; VP: Voluntary Payoff-Sharing; FP: Full Payoff-Sharing. Dots and error bars show posterior means and 90% HPDIs. Note that these visualizations show only a relevant subset of parameters from the full computational model. Statistics are derived from $n = 477$ participants (Fast resource: NP $n = 72$, VP $n = 86$, FP $n = 74$; Slow resource: NP $n = 80$, VP $n = 79$, FP $n = 86$).

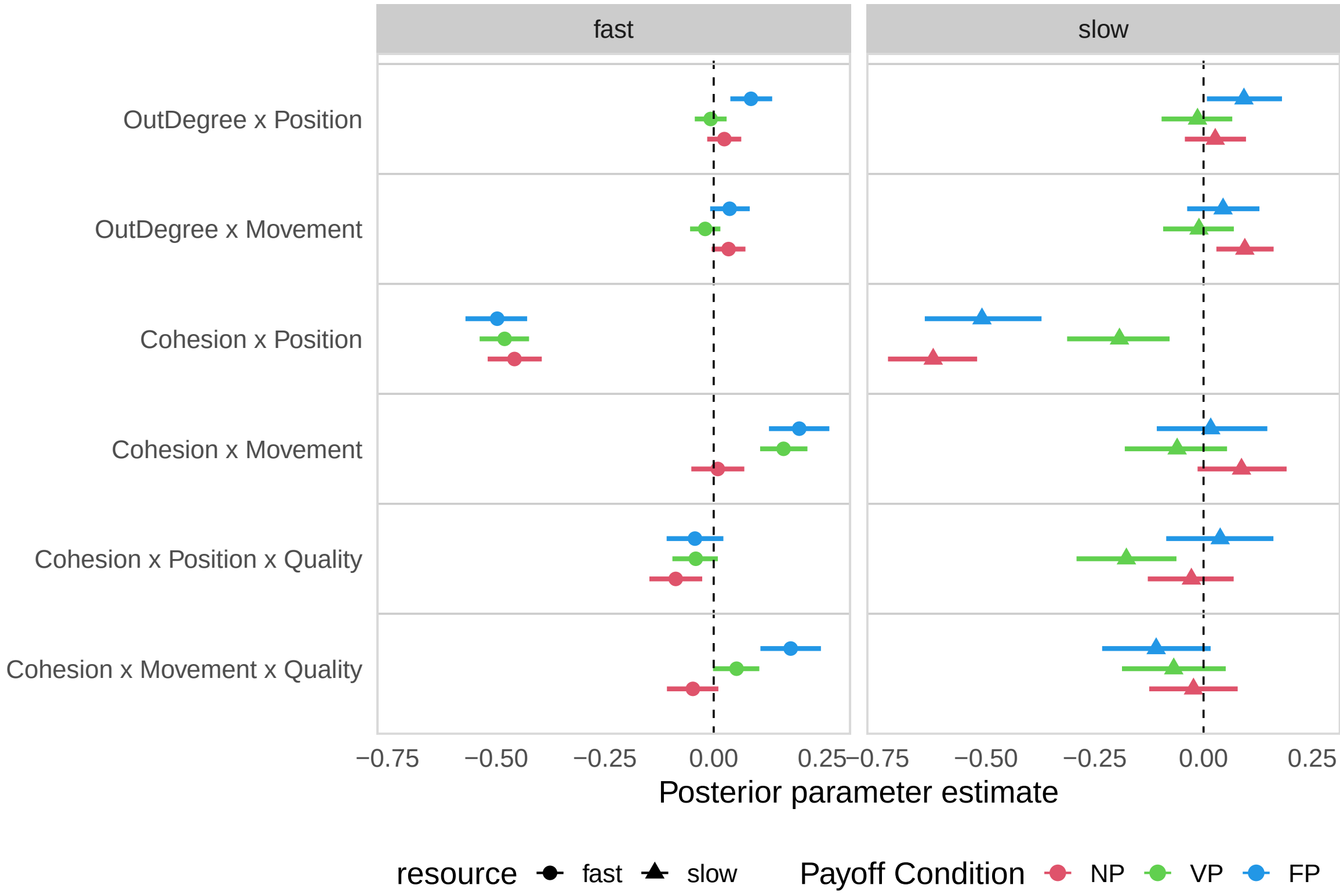

Figure 26: Impact of collective structural features on rotation decisions during search. The model was fitted to tracking states where participants observed two or more peers. High movement cohesion consistently devalues reliance on the group's spatial position (negative Cohesion x Position). Results are shown across payoff-sharing conditions and resource speeds. NP: No Payoff-Sharing; VP: Voluntary Payoff-Sharing; FP: Full Payoff-Sharing. Dots and error bars show posterior means and 90% HPDIs. Note that these visualizations show only a relevant subset of parameters from the full computational model. Statistics are derived from $n = 477$ participants (Fast resource: NP $n = 72$, VP $n = 86$, FP $n = 74$; Slow resource: NP $n = 80$, VP $n = 79$, FP $n = 86$).

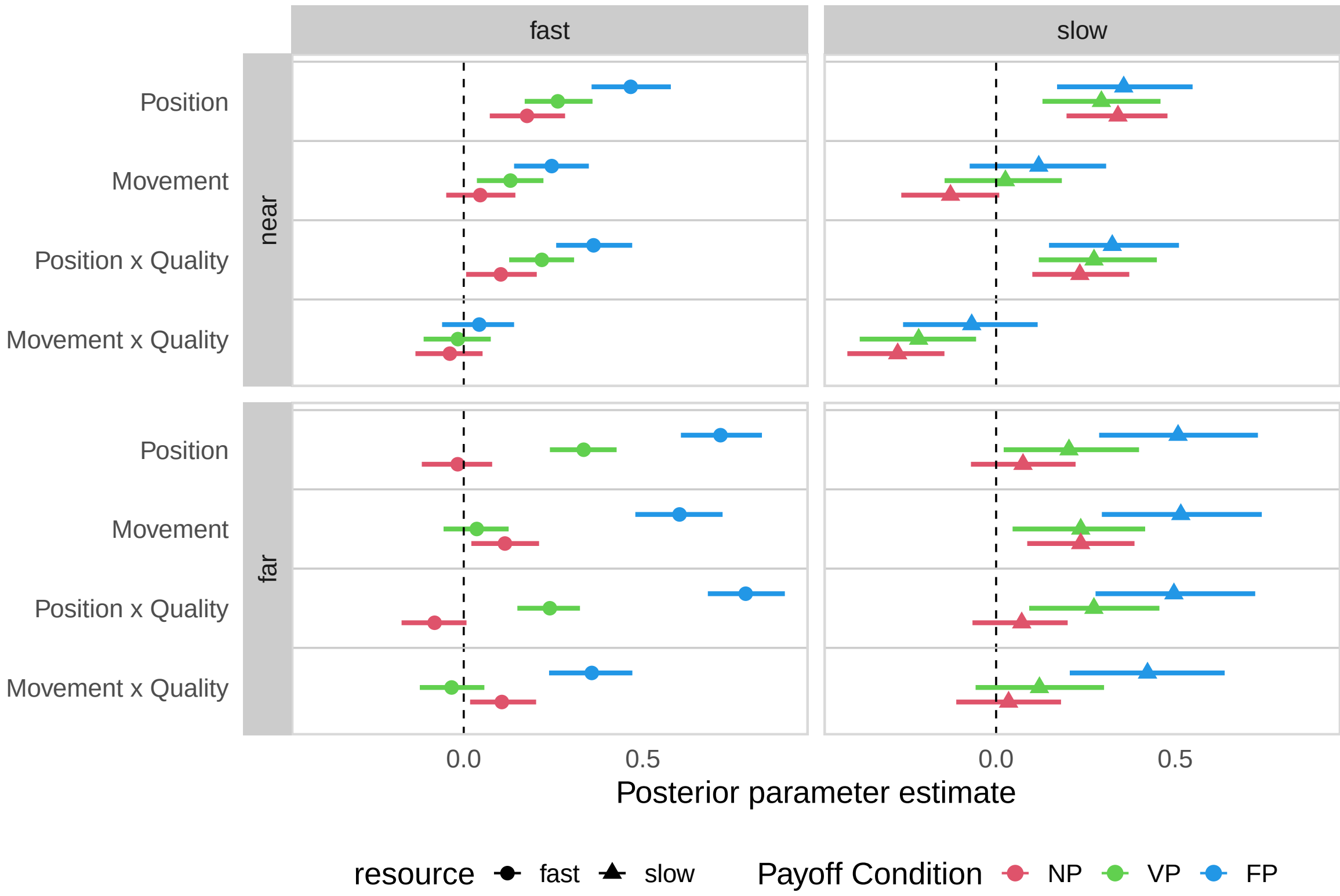

Figure 27: Impact of peer distance on rotation decisions during search. Posterior estimates of decision weights during search, restricted to observations where exactly one peer is visible. The analysis introduces a binary peer distance factor ("near" vs. "far", based on a median split) to test the effect of spatial distance on social information use. Results are shown across payoff-sharing conditions and resource speeds. NP: No Payoff-Sharing; VP: Voluntary Payoff-Sharing; FP: Full Payoff-Sharing. Dots and error bars show posterior means and 90% HPDIs. Note that these visualizations show only a relevant subset of parameters from the full computational model. Statistics are derived from n = 477 participants (Fast resource: NP n = 72, VP n = 86, FP n = 74; Slow resource: NP n = 80, VP n = 79, FP n = 86).

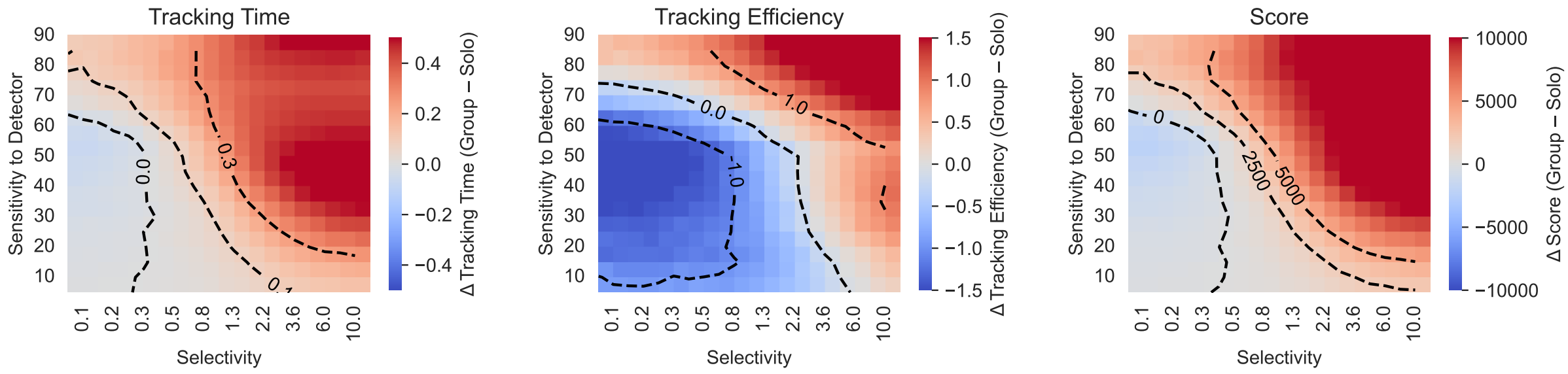

Figure 28: Effect of selectivity in the use of social information: agent-based simulation. Agent-based model parameter sweep for detector sensitivity (x-axis) and social information selectivity (y-axis). The value in each cell is calculated as the difference between average group and solitary performance. Higher detector sensitivity corresponds to a rotation that is closer to the optimal rotation for a given change in detector value. Greater selectivity corresponds to a steeper social information curve (see Main Text Fig. 5). Each panel shows the difference in key performance measures between average group performance and solitary performance: tracking time (**left**), tracking efficiency (**middle**), and total score (**right**).

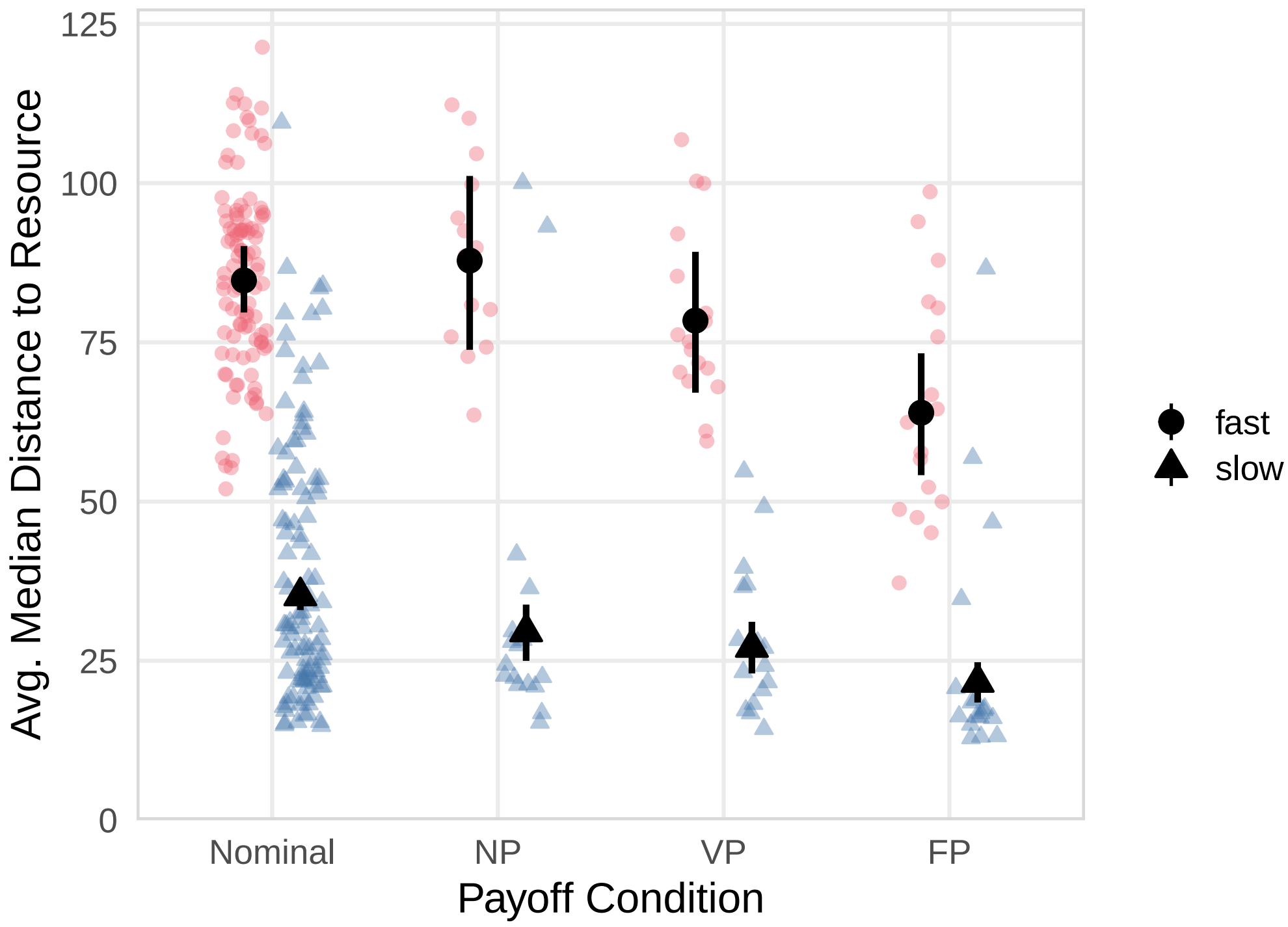

Figure 29: Average Median Distance to Resource In Groups. The average median distance to the resource center for "nominal groups" (synthetic groups constructed by randomly combining the time-series data of five solitary participants) and interacting groups (NP: No Payoff-Sharing; VP: Voluntary Payoff-Sharing; FP: Full Payoff-Sharing) across fast and slow resource speeds. For each group, the median distance of all five members to the resource center was calculated at each time step and then averaged across the entire experiment. Each dot represents an individual group. Larger black dots and error bars show posterior means and 90% HPDIs. The numerical values for the contrasts between the conditions are shown in the Table 15. Statistics are derived from $n = 621$ independent participants (Fast resource: A $n = 73$, NP $n = 72$, VP $n = 86$, FP $n = 74$; Slow resource: A $n = 71$, NP $n = 80$, VP $n = 79$, FP $n = 86$).

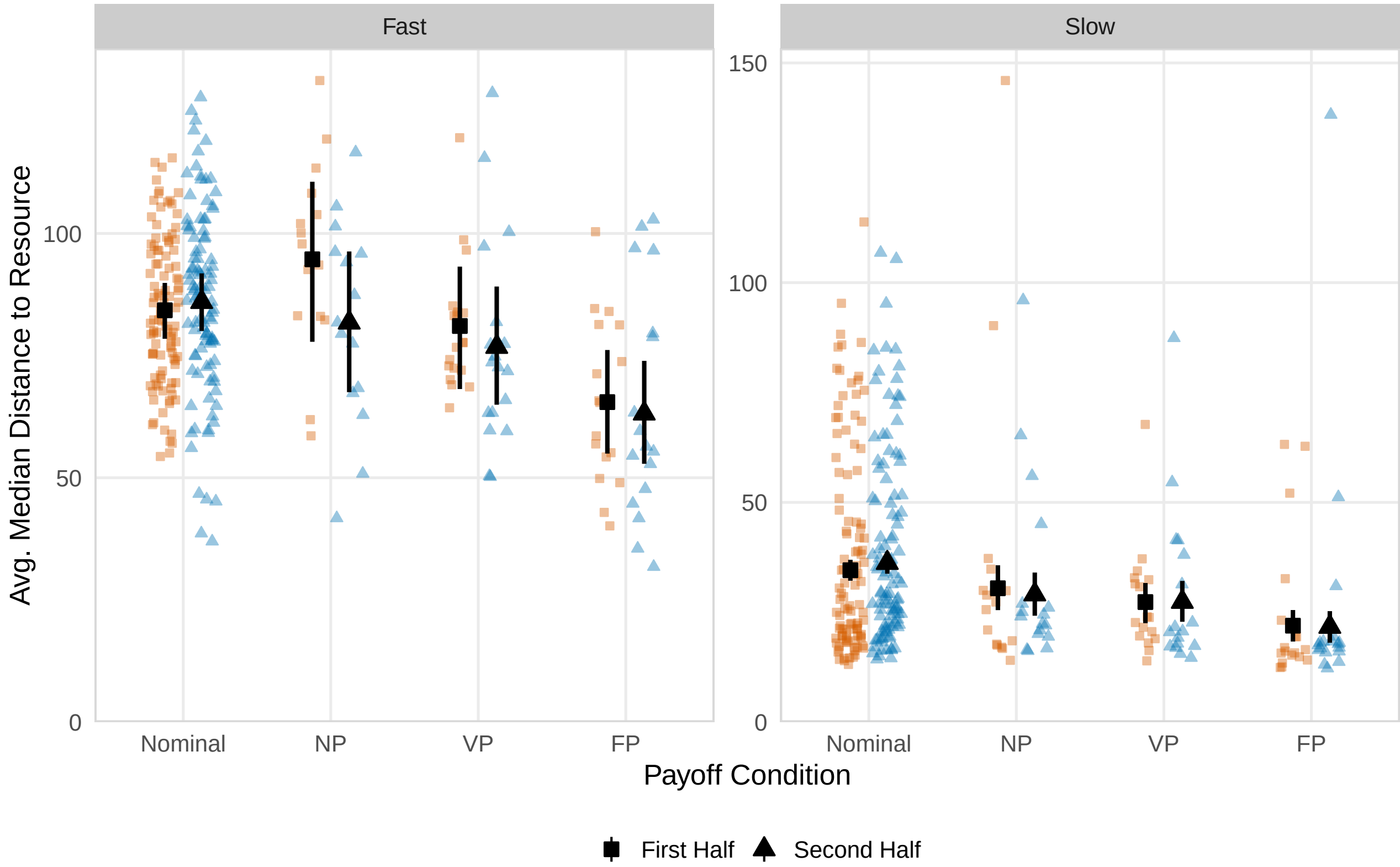

Figure 30: Average Median Distance to Resource In Groups: Temporal Contrast. The average median distance to the resource center for "nominal groups" (synthetic groups constructed by randomly combining the time-series data of five solitary participants) and interacting groups (NP: No Payoff-Sharing; VP: Voluntary Payoff-Sharing; FP: Full Payoff-Sharing) across fast and slow resource speeds, split by the first half (0–7.5 min) and second half (7.5–15 min) of the experimental session (shape and color). For each group, the median distance of all five members to the resource center was calculated at each time step and then averaged across the entire experiment. Each dot represents an individual group. Larger black dots and error bars show posterior means and 90% HPDIs. The numerical values for the contrasts between the conditions are shown in the Table 16. Statistics are derived from $n = 621$ independent participants (Fast resource: A $n = 73$, NP $n = 72$, VP $n = 86$, FP $n = 74$; Slow resource: A $n = 71$, NP $n = 80$, VP $n = 79$, FP $n = 86$).

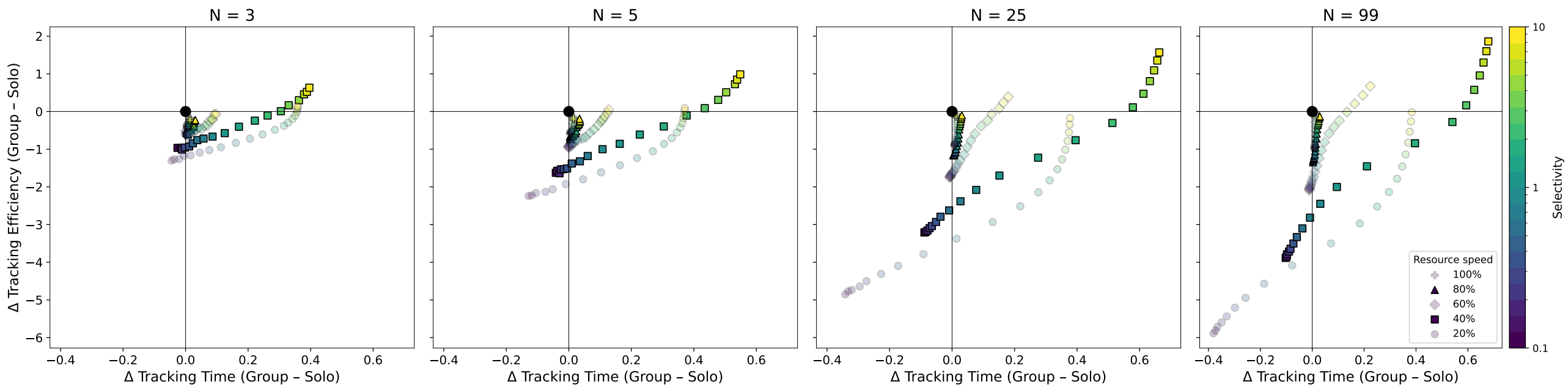

Figure 31: Effect of group size in the agent-based simulation. Average tracking time vs. tracking efficiency of groups relative to solitary agents (black dot). Colors show selectivity levels; opaque points correspond to the two experimental speeds (40% and 80%). The columns correspond to group sizes of $N \in \{3, 5, 25, 99\}$, with 5 being the group size used in the experiment.

Supplementary Tables

Table 1: Participant Distribution Across Experimental Conditions

Payoff	Resource	Participants	Groups	Groups of 5	Groups of 4
A	Fast	73	73	–	–
A	Slow	71	71	–	–
NP	Fast	72	15	12	3
NP	Slow	80	17	12	5
VP	Fast	86	19	10	9
VP	Slow	79	17	11	6
FP	Fast	74	17	6	11
FP	Slow	86	18	14	4

Note: The table shows participant and group counts by payoff sharing and resource speed conditions. "Groups of 5" and "Groups of 4" indicate the number of groups that had exactly 5 or 4 valid participants, respectively. A: Asocial Condition, NP: No Payoff-Sharing; VP: Voluntary Payoff-Sharing; FP: Full Payoff-Sharing..

Table 2: Posterior Estimates Collected Points

Resource Speed	Payoff Condition	Contrast	Mean	90% HPDI
.	.	slow - fast	**10720.57**	**[9832.10, 11606.53]**
.	A	slow - fast	**8449.69**	**[7053.20, 9863.92]**
.	FP	slow - fast	**11975.64**	**[10337.88, 13668.39]**
.	NP	slow - fast	**10571.08**	**[8863.52, 12276.49]**
.	VP	slow - fast	**11885.87**	**[10325.95, 13444.91]**
**fast**				
fast	.	FP - A	**1710.26**	**[572.81, 2803.50]**
fast	.	FP - NP	**2544.92**	**[1364.50, 3718.67]**
fast	.	FP - VP	**2008.92**	**[843.34, 3194.27]**
fast	.	NP - A	**-834.66**	**[-1654.19, -35.59]**
fast	.	VP - A	-298.66	[-1169.61, 537.86]
fast	.	VP - NP	536.00	[-385.41, 1471.24]
**slow**				
slow	.	FP - A	**5236.22**	**[3373.83, 7129.94]**
slow	.	FP - NP	**3949.48**	**[1876.26, 6081.79]**
slow	.	FP - VP	**2098.69**	**[84.14, 4069.94]**
slow	.	NP - A	1286.73	[-790.97, 3366.14]
slow	.	VP - A	**3137.53**	**[1126.32, 5079.08]**
slow	.	VP - NP	1850.79	[-316.14, 4021.80]

Note: Contrasts where the 90% HPDI does not include zero are highlighted in bold. '90% HPDI' refers to the 90% highest posterior density interval. 'Mean' is the mean of the posterior distribution for each contrast.

Table 3: Posterior Estimates for Gross Collected Points (Signaling Costs Reimbursed)

Resource Speed	Payoff Condition	Contrast	Mean	90% HPDI
.	.	slow - fast	**10729.14**	**[9853.33, 11616.74]**
.	A	slow - fast	**8447.48**	**[7061.82, 9863.63]**
.	FP	slow - fast	**11973.37**	**[10314.00, 13620.26]**
.	NP	slow - fast	**10572.07**	**[8906.07, 12281.47]**
.	VP	slow - fast	**11923.66**	**[10380.76, 13467.63]**
**fast**				
fast	.	FP - A	**1696.47**	**[599.48, 2832.15]**
fast	.	FP - NP	**2540.12**	**[1355.01, 3709.39]**
fast	.	FP - VP	**1930.74**	**[750.34, 3114.67]**
fast	.	NP - A	**-843.64**	**[-1648.41, -49.21]**
fast	.	VP - A	-234.27	[-1086.06, 626.63]
fast	.	VP - NP	609.38	[-304.97, 1541.83]
**slow**				
slow	.	FP - A	**5222.36**	**[3394.47, 7141.53]**
slow	.	FP - NP	**3941.42**	**[1798.91, 5996.15]**
slow	.	FP - VP	1980.45	[-32.45, 3895.83]
slow	.	NP - A	1280.94	[-816.30, 3308.85]
slow	.	VP - A	**3241.91**	**[1292.60, 5185.01]**
slow	.	VP - NP	1960.97	[-178.00, 4105.57]

Note: This table presents the posterior estimates for participants' gross scores, which are calculated by taking the final collected points and adding back any points spent on voluntary payoff sharing (at a rate of 1 point per second of sharing). Contrasts where the 90% HPDI does not include zero are highlighted in bold. '90% HPDI' refers to the 90% highest posterior density interval. 'Mean' is the mean of the posterior distribution for each contrast.

Table 4: Posterior Estimates for Collected Points: Temporal Contrasts

Resource Speed	Payoff Condition	Contrast	Mean	90% HPDI
**fast**				
fast	A	Second - First Half	-240.77	[-584.88, 95.41]
fast	NP	Second - First Half	47.17	[-207.16, 296.25]
fast	VP	Second - First Half	-207.14	[-442.52, 28.37]
fast	FP	Second - First Half	75.38	[-268.80, 429.49]
**slow**				
slow	A	Second - First Half	-678.51	[-1563.01, 149.61]
slow	NP	Second - First Half	39.16	[-762.16, 812.25]
slow	VP	Second - First Half	235.21	[-316.94, 778.27]
slow	FP	Second - First Half	-600.23	[-1341.12, 140.80]

Note: This table shows the posterior estimates of changes in participants' scores between the first (0–7.5 min) and second (7.5-15min) halves of the experiment, broken down by experimental condition. Contrasts where the 90% HPDI does not include zero are highlighted in bold. '90% HPDI' refers to the 90% highest posterior density interval. 'Mean' is the mean of the posterior distribution for each contrast.

Table 5: Posterior Estimates Collected Points Precision

Resource Speed	Payoff Condition	Contrast	Mean	90% HPDI
.	.	slow - fast	**0.42**	**[0.38, 0.45]**
.	A	slow - fast	**0.33**	**[0.28, 0.38]**
.	FP	slow - fast	**0.47**	**[0.40, 0.53]**
.	NP	slow - fast	**0.41**	**[0.35, 0.48]**
.	VP	slow - fast	**0.46**	**[0.40, 0.52]**
**fast**				
fast	.	FP - A	**0.07**	**[0.02, 0.11]**
fast	.	VP - A	-0.01	[-0.05, 0.02]
fast	.	NP - A	-0.03	[-0.06, -0.00]
fast	.	FP - NP	**0.10**	**[0.05, 0.15]**
fast	.	FP - VP	**0.08**	**[0.03, 0.12]**
fast	.	VP - NP	0.02	[-0.02, 0.06]
**slow**				
slow	.	FP - A	**0.20**	**[0.13, 0.28]**
slow	.	VP - A	**0.12**	**[0.04, 0.20]**
slow	.	NP - A	0.05	[-0.03, 0.13]
slow	.	FP - NP	**0.15**	**[0.07, 0.24]**
slow	.	FP - VP	0.08	[0.00, 0.16]
slow	.	VP - NP	0.07	[-0.01, 0.16]

Note: Contrasts where the 90% HPDI does not include zero are highlighted in bold. The column labeled 'Mean' gives the posterior mean of the difference in the Beta precision parameter (ϕ) between conditions, on the response scale (not log-transformed). The '90% HPDI' shows the 90% highest posterior density interval for each contrast. Higher values of ϕ indicate greater precision (i.e., less variability) in the outcome distribution.

Table 6: Posterior Estimates Tracking Time (minutes)

Resource Speed	Payoff Condition	Contrast	Mean	90% HPDI
.	.	slow - fast	**7.44**	**[6.89, 8.00]**
.	A	slow - fast	**5.96**	**[5.13, 6.80]**
.	FP	slow - fast	**7.43**	**[6.40, 8.44]**
.	NP	slow - fast	**7.97**	**[6.86, 9.03]**
.	VP	slow - fast	**8.41**	**[7.47, 9.39]**
**fast**				
fast	.	FP - A	**2.00**	**[0.96, 3.04]**
fast	.	FP - NP	**2.33**	**[1.07, 3.55]**
fast	.	FP - VP	**1.76**	**[0.56, 3.01]**
fast	.	NP - A	-0.34	[-1.22, 0.56]
fast	.	VP - A	0.23	[-0.66, 1.13]
fast	.	VP - NP	0.57	[-0.52, 1.68]
**slow**				
slow	.	FP - A	**3.47**	**[2.63, 4.32]**
slow	.	FP - NP	**1.80**	**[0.84, 2.74]**
slow	.	FP - VP	**0.78**	**[0.01, 1.56]**
slow	.	NP - A	**1.67**	**[0.56, 2.71]**
slow	.	VP - A	**2.68**	**[1.75, 3.63]**
slow	.	VP - NP	1.01	[-0.03, 2.05]

Note: Contrasts where the 90% HPDI does not include zero are highlighted in bold. ‘90% HPDI’ refers to the 90% highest posterior density interval. ‘Mean’ is the mean of the posterior distribution for each contrast.

Table 7: Posterior Estimates Tracking Efficiency (points/second)

Resource Speed	Payoff Condition	Contrast	Mean	90% HPDI
.	.	slow - fast	**5.62**	**[5.13, 6.11]**
.	A	slow - fast	**4.90**	**[3.95, 5.85]**
.	FP	slow - fast	**6.31**	**[5.34, 7.29]**
.	NP	slow - fast	**5.14**	**[4.12, 6.13]**
.	VP	slow - fast	**6.14**	**[5.19, 7.08]**
**fast**				
fast	.	FP - A	-0.36	[-1.21, 0.46]
fast	.	FP - NP	**1.17**	**[0.25, 2.09]**
fast	.	FP - VP	**1.52**	**[0.64, 2.37]**
fast	.	NP - A	**-1.53**	**[-2.37, -0.68]**
fast	.	VP - A	**-1.88**	**[-2.64, -1.09]**
fast	.	VP - NP	-0.35	[-1.23, 0.52]
**slow**				
slow	.	FP - A	1.05	[-0.02, 2.16]
slow	.	FP - NP	**2.34**	**[1.25, 3.38]**
slow	.	FP - VP	**1.69**	**[0.63, 2.73]**
slow	.	NP - A	**-1.29**	**[-2.38, -0.16]**
slow	.	VP - A	-0.64	[-1.73, 0.46]
slow	.	VP - NP	0.65	[-0.42, 1.72]

Note: Contrasts where the 90% HPDI does not include zero are highlighted in bold. '90% HPDI' refers to the 90% highest posterior density interval. 'Mean' is the mean of the posterior distribution for each contrast.

Table 8: Posterior Estimates In Degree

Resource Speed	Payoff Condition	Contrast	Mean	90% HPDI
.	FP	slow - fast	0.02	[-0.03, 0.06]
.	NP	slow - fast	0.04	[-0.01, 0.08]
.	VP	slow - fast	0.03	[-0.01, 0.07]
**fast**				
fast	.	FP - NP	**0.08**	**[0.03, 0.12]**
fast	.	FP - VP	0.03	[-0.02, 0.07]
fast	.	VP - NP	**0.05**	**[0.01, 0.09]**
**slow**				
slow	.	FP - NP	**0.06**	**[0.01, 0.10]**
slow	.	FP - VP	0.01	[-0.03, 0.06]
slow	.	VP - NP	0.04	[0.00, 0.09]

Note: Contrasts where the 90% HPDI does not include zero are highlighted in bold. '90% HPDI' refers to the 90% highest posterior density interval. 'Mean' is the mean of the posterior distribution for each contrast.

Table 9: Posterior Estimates Out Degree

Resource Speed	Payoff Condition	Contrast	Mean	90% HPDI
.	FP	slow - fast	0.01	[-0.04, 0.06]
.	NP	slow - fast	0.02	[-0.03, 0.07]
.	VP	slow - fast	0.02	[-0.03, 0.07]
**fast**				
fast	.	FP - NP	**0.07**	**[0.02, 0.12]**
fast	.	FP - VP	0.04	[-0.01, 0.09]
fast	.	VP - NP	0.03	[-0.02, 0.08]
**slow**				
slow	.	FP - NP	**0.06**	**[0.01, 0.11]**
slow	.	FP - VP	0.03	[-0.02, 0.08]
slow	.	VP - NP	0.03	[-0.02, 0.08]

Note: Contrasts where the 90% HPDI does not include zero are highlighted in bold. '90% HPDI' refers to the 90% highest posterior density interval. 'Mean' is the mean of the posterior distribution for each contrast.

Table 10: Posterior Estimates Adaptive Connectivity: Probability to Follow Adaptive Social Information.

Resource	Payoff	State	Contrast	Mean	90% HPDI
.	FP	searching	slow - fast	**0.03**	**[0.01, 0.05]**
.	NP	searching	slow - fast	**0.11**	**[0.09, 0.14]**
.	VP	searching	slow - fast	**0.08**	**[0.05, 0.10]**
.	FP	tracking	slow - fast	**-0.02**	**[-0.04, -0.01]**
.	NP	tracking	slow - fast	**0.07**	**[0.05, 0.09]**
.	VP	tracking	slow - fast	**0.05**	**[0.03, 0.07]**
**fast**					
fast	FP	.	searching - tracking	**-0.02**	**[-0.03, -0.01]**
fast	NP	.	searching - tracking	**-0.08**	**[-0.10, -0.07]**
fast	VP	.	searching - tracking	**-0.05**	**[-0.06, -0.04]**
fast	.	searching	FP - NP	**0.20**	**[0.18, 0.23]**
fast	.	searching	FP - VP	**0.13**	**[0.11, 0.15]**
fast	.	searching	VP - NP	**0.08**	**[0.06, 0.10]**
fast	.	tracking	FP - NP	**0.14**	**[0.12, 0.16]**
fast	.	tracking	FP - VP	**0.10**	**[0.08, 0.12]**
fast	.	tracking	VP - NP	**0.04**	**[0.02, 0.07]**
**slow**					
slow	FP	.	searching - tracking	**0.03**	**[0.02, 0.05]**
slow	NP	.	searching - tracking	**-0.04**	**[-0.06, -0.03]**
slow	VP	.	searching - tracking	**-0.03**	**[-0.04, -0.01]**
slow	.	searching	FP - NP	**0.12**	**[0.10, 0.15]**
slow	.	searching	FP - VP	**0.08**	**[0.06, 0.11]**
slow	.	searching	VP - NP	**0.04**	**[0.02, 0.06]**
slow	.	tracking	FP - NP	**0.05**	**[0.03, 0.06]**
slow	.	tracking	FP - VP	**0.02**	**[0.01, 0.04]**
slow	.	tracking	VP - NP	**0.02**	**[0.01, 0.04]**

Note: All contrasts are calculated conditional on **adaptive social information being available**. Contrasts where the 90% HPDI does not include zero are highlighted in bold. '90% HPDI' refers to the 90% highest posterior density interval. 'Mean' is the mean of the posterior distribution for each contrast.

Table 11: Number of Resource Discovery Events per Condition

**Payoff Condition**	**Resource Speed**	**Unique Events**
A	Fast	383
NP	Fast	126
VP	Fast	173
FP	Fast	147
A	Slow	212
NP	Slow	17
VP	Slow	21
FP	Slow	11

Note: The table shows the number of unique resource discovery events by payoff sharing and resource speed conditions. A: Asocial Condition, NP: No Payoff-Sharing; VP: Voluntary Payoff-Sharing; FP: Full Payoff-Sharing.

Table 12: Percentage of Time Participants Have Others in Their FOV

**Others in FOV**	**Overall**	**Searching**	**Tracking**
0	42.85%	43.51%	42.35%
1	25.96%	24.58%	27.00%
2	16.26%	15.51%	16.83%
3	10.03%	10.46%	9.70%
4	4.90%	5.94%	4.12%

Note: The table shows the percentage of time participants have 0, 1, 2, 3, or 4 others in their field of view, split by searching and tracking behavior states. Only group conditions (NP, VP, and FP) were included.

Table 13: Posterior Estimates for Temporal Dynamics for Rotation Magnitude Model - Tracking

Resource	Payoff	Decision Weight	Contrast	Mean	90% HPDI
.	.	.	Second - First Half	**-0.11**	**[-0.15, -0.07]**
slow	.	.	Second - First Half	**0.16**	**[0.12, 0.21]**
.	NP	.	Second - First Half	0.01	[-0.06, 0.07]
.	VP	.	Second - First Half	0.05	[-0.01, 0.10]
.	FP	.	Second - First Half	**0.13**	**[0.08, 0.18]**
slow	NP	.	Second - First Half	-0.02	[-0.09, 0.06]
slow	VP	.	Second - First Half	-0.05	[-0.11, 0.02]
slow	FP	.	Second - First Half	**-0.15**	**[-0.22, -0.09]**
slow	A	Detector Change	Second - First Half	**-0.14**	**[-0.19, -0.08]**
slow	A	Distance	Second - First Half	0.02	[-0.03, 0.07]
slow	NP	Detector Change	Second - First Half	**-0.13**	**[-0.17, -0.09]**
slow	NP	Distance	Second - First Half	**0.06**	**[0.02, 0.10]**
slow	NP	Position	Second - First Half	-0.02	[-0.06, 0.02]
slow	NP	Movement	Second - First Half	**0.08**	**[0.04, 0.13]**
slow	NP	Position x Quality	Second - First Half	0.05	[-0.01, 0.11]
slow	NP	Movement x Quality	Second - First Half	0.01	[-0.04, 0.08]
slow	VP	Detector Change	Second - First Half	**-0.05**	**[-0.09, -0.02]**
slow	VP	Distance	Second - First Half	0.02	[-0.02, 0.07]
slow	VP	Position	Second - First Half	0.00	[-0.04, 0.05]
slow	VP	Movement	Second - First Half	0.04	[-0.00, 0.09]
slow	VP	Position x Quality	Second - First Half	**-0.10**	**[-0.17, -0.04]**
slow	VP	Movement x Quality	Second - First Half	0.04	[-0.02, 0.09]
slow	FP	Detector Change	Second - First Half	**-0.08**	**[-0.12, -0.04]**
slow	FP	Distance	Second - First Half	**-0.14**	**[-0.19, -0.09]**
slow	FP	Position	Second - First Half	**0.09**	**[0.05, 0.13]**
slow	FP	Movement	Second - First Half	0.02	[-0.02, 0.07]
slow	FP	Position x Quality	Second - First Half	0.00	[-0.06, 0.06]
slow	FP	Movement x Quality	Second - First Half	**0.11**	**[0.05, 0.18]**
fast	A	Detector Change	Second - First Half	**-0.14**	**[-0.20, -0.08]**
fast	A	Distance	Second - First Half	0.05	[-0.01, 0.12]
fast	NP	Detector Change	Second - First Half	**-0.11**	**[-0.18, -0.04]**
fast	NP	Distance	Second - First Half	**-0.08**	**[-0.17, -0.01]**
fast	NP	Position	Second - First Half	-0.04	[-0.16, 0.07]
fast	NP	Movement	Second - First Half	-0.03	[-0.13, 0.08]
fast	NP	Position x Quality	Second - First Half	-0.04	[-0.18, 0.08]
fast	NP	Movement x Quality	Second - First Half	-0.03	[-0.15, 0.09]
fast	VP	Detector Change	Second - First Half	-0.01	[-0.06, 0.04]
fast	VP	Distance	Second - First Half	0.03	[-0.03, 0.09]
fast	VP	Position	Second - First Half	0.05	[-0.04, 0.13]
fast	VP	Movement	Second - First Half	-0.04	[-0.11, 0.04]
fast	VP	Position x Quality	Second - First Half	0.08	[-0.03, 0.17]
fast	VP	Movement x Quality	Second - First Half	-0.02	[-0.11, 0.07]
fast	FP	Detector Change	Second - First Half	**-0.09**	**[-0.13, -0.04]**
fast	FP	Distance	Second - First Half	0.01	[-0.05, 0.07]
fast	FP	Position	Second - First Half	0.06	[-0.01, 0.13]
fast	FP	Movement	Second - First Half	0.03	[-0.03, 0.10]
fast	FP	Position x Quality	Second - First Half	0.05	[-0.03, 0.14]
fast	FP	Movement x Quality	Second - First Half	0.01	[-0.09, 0.09]

Note: Posterior means and 90% highest posterior density intervals (HPDIs) for the differences in the computational model parameters (baseline rotation and decision weights) between the second and first half of the experiment during tracking. Contrasts where the 90% HPDI does not include zero are highlighted in bold. Note that these estimates present only a relevant subset of parameters from the full computational model.

Table 14: Posterior Estimates for Temporal Dynamics for Rotation Magnitude Model - Search

Resource	Payoff	Decision Weight	Contrast	Mean	90% HPDI
**Resource Speed: .**					
.	.	.	Second - First Half	**-0.18**	**[-0.20, -0.17]**
slow	.	.	Second - First Half	**0.08**	**[0.05, 0.12]**
.	NP	.	Second - First Half	**0.20**	**[0.18, 0.23]**
.	VP	.	Second - First Half	**0.23**	**[0.21, 0.26]**
.	FP	.	Second - First Half	**0.27**	**[0.24, 0.30]**
slow	NP	.	Second - First Half	0.03	[-0.03, 0.08]
slow	VP	.	Second - First Half	**-0.17**	**[-0.24, -0.11]**
slow	FP	.	Second - First Half	**-0.10**	**[-0.15, -0.03]**
slow	NP	Position	Second - First Half	-0.08	[-0.17, 0.00]
slow	NP	Movement	Second - First Half	**0.24**	**[0.16, 0.33]**
slow	NP	Position x Quality	Second - First Half	-0.07	[-0.16, 0.01]
slow	NP	Movement x Quality	Second - First Half	**0.17**	**[0.08, 0.25]**
slow	VP	Position	Second - First Half	-0.03	[-0.15, 0.10]
slow	VP	Movement	Second - First Half	-0.09	[-0.20, 0.01]
slow	VP	Position x Quality	Second - First Half	**0.13**	**[0.02, 0.25]**
slow	VP	Movement x Quality	Second - First Half	**0.30**	**[0.19, 0.41]**
slow	FP	Position	Second - First Half	0.11	[-0.01, 0.23]
slow	FP	Movement	Second - First Half	**-0.18**	**[-0.29, -0.05]**
slow	FP	Position x Quality	Second - First Half	-0.08	[-0.20, 0.03]
slow	FP	Movement x Quality	Second - First Half	0.12	[-0.00, 0.23]
fast	NP	Position	Second - First Half	**0.14**	**[0.09, 0.20]**
fast	NP	Movement	Second - First Half	**-0.06**	**[-0.12, -0.01]**
fast	NP	Position x Quality	Second - First Half	0.04	[-0.02, 0.09]
fast	NP	Movement x Quality	Second - First Half	-0.00	[-0.06, 0.05]
fast	VP	Position	Second - First Half	**0.07**	**[0.03, 0.12]**
fast	VP	Movement	Second - First Half	**0.10**	**[0.06, 0.15]**
fast	VP	Position x Quality	Second - First Half	**0.10**	**[0.05, 0.14]**
fast	VP	Movement x Quality	Second - First Half	-0.00	[-0.05, 0.04]
fast	FP	Position	Second - First Half	**0.15**	**[0.09, 0.21]**
fast	FP	Movement	Second - First Half	**0.09**	**[0.03, 0.15]**
fast	FP	Position x Quality	Second - First Half	**0.16**	**[0.10, 0.22]**
fast	FP	Movement x Quality	Second - First Half	0.04	[-0.02, 0.09]

Note: Posterior means and 90% highest posterior density intervals (HPDIs) for the differences in the computational model parameters (baseline rotation and decision weights) between the second and first half of the experiment during the search. Contrasts where the 90% HPDI does not include zero are highlighted in bold. Note that these estimates present only a relevant subset of parameters from the full computational model.

Table 15: Posterior Estimates Average Median Distance

Resource Speed	Payoff Condition	Contrast	Mean	90% HPDI
.	.	fast - slow	**50.38**	**[44.93, 55.87]**
.	Nominal	fast - slow	**49.53**	**[44.05, 55.03]**
.	NP	fast - slow	**58.30**	**[43.69, 72.19]**
.	VP	fast - slow	**51.32**	**[39.28, 62.57]**
.	FP	fast - slow	**42.35**	**[32.35, 52.22]**
**fast**				
fast	.	Nominal - FP	**20.77**	**[9.91, 31.46]**
fast	.	Nominal - NP	-3.12	[-16.94, 12.08]
fast	.	Nominal - VP	6.31	[-5.91, 18.21]
fast	.	NP - FP	**23.89**	**[6.55, 40.48]**
fast	.	NP - VP	9.43	[-8.14, 27.00]
fast	.	VP - FP	**14.46**	**[0.03, 29.33]**
**slow**				
slow	.	Nominal - FP	**13.58**	**[9.69, 17.35]**
slow	.	Nominal - NP	**5.65**	**[0.96, 10.70]**
slow	.	Nominal - VP	**8.09**	**[3.66, 12.74]**
slow	.	NP - FP	**7.93**	**[2.40, 13.34]**
slow	.	NP - VP	2.44	[-3.73, 8.32]
slow	.	VP - FP	**5.49**	**[0.34, 10.66]**

Note: Results from a Bayesian Generalized Linear Model predicting the average median distance of groups to the resource center. Contrasts where the 90% HPDI does not include zero are highlighted in bold.

Table 16: Posterior Estimates Average Median Distance: Temporal Contrast

Resource Speed	Payoff Condition	Contrast	Mean	90% HPDI
**fast**				
fast	Nominal	Second - First Half	1.70	[-3.47, 6.79]
fast	NP	Second - First Half	-12.95	[-26.89, 1.04]
fast	VP	Second - First Half	-4.27	[-15.26, 6.76]
fast	FP	Second - First Half	-2.34	[-11.84, 7.18]
**slow**				
slow	Nominal	Second - First Half	1.67	[-0.59, 3.84]
slow	NP	Second - First Half	-1.39	[-5.83, 3.27]
slow	VP	Second - First Half	0.06	[-4.12, 4.39]
slow	FP	Second - First Half	-0.26	[-3.51, 3.01]

Note: Results from a Bayesian Generalized Linear Model predicting the average median distance of groups to the resource center, split by the first half (0–7.5 min) and second half (7.5–15 min) of the experimental session. Negative values indicate that performance improved in the second half of the experiment compared to the first. Contrasts where the 90% HPDI does not include zero are highlighted in bold.

Supplementary Note 1: Optimal rotation based on private information

Here, we derive a normative model to determine the optimal rotation an agent should make based solely on the change in its distance to a moving resource (δ_t). The derivation consists of two parts. First, a **forward model** calculates how the distance to the resource changes as a function of the agent's movement relative to resource. This answers the question: Given my movement, what distance change should I expect? Second, an **inverse model** uses Bayesian inference to invert this logic[1,2], answering the question: Given the distance change I just observed, what is the optimal way to turn?

1.1 Forward model: calculating distance change

The forward model calculates the rate of change in distance ($\frac{\mathrm{d}}{\mathrm{d}t}r$) between an agent and a resource, given their speeds and relative orientation. We establish a reference frame that moves with the resource, with the resource's velocity vector, $\mathbf{v}_p$ (see Fig. 1A). The relevant variables are: The agent's constant speed (v_a); The resource's constant speed (v_p); The agent's heading, relative to the resource's direction of motion (ϕ_a); The angle of the agent's position, relative to the resource's direction of motion (ξ).

The rate of change in distance is the projection of the relative velocity vector ($\mathbf{v}_a - \mathbf{v}_p$) onto the unit vector ($\mathbf{u}_r$) that points from the resource to the agent.

$$\frac{\mathrm{d}}{\mathrm{d}t}r = (\mathbf{v}_a - \mathbf{v}_p) \cdot \mathbf{u}_r \tag{1}$$

In the resource frame of reference, the vectors are: resource velocity: $\mathbf{v}_p = v_p \begin{bmatrix} \cos(0) \\ \sin(0) \end{bmatrix}$; agent velocity: $\mathbf{v}_a = v_a \begin{bmatrix} \cos(\phi_a) \\ \sin(\phi_a) \end{bmatrix}$; unit vector to agent: $\mathbf{u}_r = \begin{bmatrix} \cos(\xi) \\ \sin(\xi) \end{bmatrix}$. Performing the dot product:

$$\begin{aligned} \frac{\mathrm{d}}{\mathrm{d}t}r &= \begin{bmatrix} v_a\cos(\phi_a) - v_p\cos(0) \\ v_a\sin(\phi_a) - v_p\sin(0) \end{bmatrix} \cdot \begin{bmatrix} \cos(\xi) \\ \sin(\xi) \end{bmatrix} \\ &= \begin{bmatrix} v_a\cos(\phi_a) - v_p \\ v_a\sin(\phi_a) \end{bmatrix} \cdot \begin{bmatrix} \cos(\xi) \\ \sin(\xi) \end{bmatrix} \\ &= (v_a\cos(\phi_a) - v_p)\cdot\cos(\xi) + v_a\sin(\phi_a)\cdot\sin(\xi) \\ &= v_a\cdot(\cos(\phi_a)\cdot\cos(\xi) + \sin(\phi_a)\cdot\sin(\xi)) - v_p\cdot\cos(\xi) \end{aligned} \tag{2}$$

Using the trigonometric identity for the cosine of a difference (i.e., $\cos(\alpha-\beta) = \cos(\alpha)\cos(\beta) + \sin(\alpha)\sin(\beta)$) we arrive at the final expression for the forward model (see Fig. 1B):

$$\frac{\mathrm{d}}{\mathrm{d}t}r = v_a\cos(\phi_a - \xi) - v_p\cos(\xi) \tag{3}$$

1.2 Optimal rotation

To find the optimal rotation, ψ, an agent must choose the turn that will most rapidly decrease its distance to the resource. This corresponds to minimizing the future rate of distance change. After a rotation ψ, the agent's new heading will be $\phi_a' = \phi_a + \psi$. We therefore minimize the following function (forward model) with respect to ψ:

$$f(\psi) = v_a \cos(\phi_a + \psi - \xi) - v_p \cos(\xi) \tag{4}$$

The first derivative with respect to ψ is:

$$\frac{\mathrm{d}f}{\mathrm{d}\psi} = -v_a \sin(\phi_a + \psi - \xi) \tag{5}$$

Setting the first derivative to zero finds the extrema:

$$-v_a \sin(\phi_a + \psi - \xi) = 0 \Rightarrow \psi = \pm\pi + \xi - \phi_a \vee \psi = \xi - \phi_a \tag{6}$$

The second derivative with respect to ψ is:

$$\frac{\mathrm{d}^2 f}{\mathrm{d}\psi^2} = -v_a \cos(\phi_a + \psi - \xi) \tag{7}$$

The second derivative of the function should be positive (indicating a minimum):

$$\begin{aligned} -v_a \cos(\phi_a + \psi - \xi) &> 0 \\ \cos(\phi_a + \psi - \xi) &< 0 \\ -\frac{\pi}{2} > \phi_a + \psi - \xi &\vee \phi_a + \psi - \xi > \frac{\pi}{2} \\ -\frac{\pi}{2} + \xi - \phi_a > \psi &\vee \psi > \frac{\pi}{2} + \xi - \phi_a \end{aligned} \tag{8}$$

Thus, the optimal rotation is (see Fig. 1C):

$$\psi_{\text{opt}} = \pm\pi + \xi - \phi_a \tag{9}$$

Note that based on the distance change δ alone there are **two** possible optimal solutions.

1.3 Inference model: inferring rotation from distance change

In the hidden resource tracking task, an agent doesn't know the true angles ξ and ϕ_a; it only observes the resulting distance change, $\delta_t \approx \frac{\mathrm{d}}{\mathrm{d}t} r$. The inverse model uses this observation to infer a probability distribution over possible optimal rotations. Using Bayes' rule, we seek the posterior probability of a rotation ψ given an observed distance change δ: $P(\psi|\delta)$. We can find this by marginalizing over the unknown (latent) angles ξ and ϕ_a:

$$P(\psi|\delta) = \int_{-\pi}^{+\pi} \int_{-\pi}^{+\pi} p(\psi|\delta, \xi, \phi_a) \cdot p(\xi, \phi_a)\, \mathrm{d}\xi\, \mathrm{d}\phi_a \tag{10}$$

We make three simplifying assumptions. First, we assume the optimal rotation is determined by the latent angles. Once ξ and ϕ_a are known, the optimal rotation ψ_{opt} is fixed (see Eq. 9).

Second, we assume conditional independence of ψ and δ given ξ and ϕ_a, and therefore express $P(\psi|\xi, \phi_a, \delta)$ as following:

$$P(\psi|\xi, \phi_a, \delta) \propto P(\psi|\xi, \phi_a) \cdot P(\delta|\xi, \phi_a) \cdot P(\xi, \phi_a) \tag{11}$$

Third, we assume a uniform prior over angles, meaning the agent has no prior knowledge of its relative orientation, so $p(\xi, \phi_a)$ is a uniform distribution. Under these assumptions, Bayes' rule simplifies the posterior to:

$$P(\psi|\delta) \propto \int_{-\pi}^{+\pi} \int_{-\pi}^{+\pi} p(\psi|\xi, \phi_a) \cdot p(\delta|\xi, \phi_a)\, \mathrm{d}\xi\, \mathrm{d}\phi_a \tag{12}$$

Here, $p(\delta|\xi, \phi_a)$ is the likelihood of observing δ given a specific orientation, which is derived from the forward model. $p(\psi|\xi, \phi_a)$ is the policy that executes the optimal rotation ψ_{opt} for a given orientation and could be substituted with the expression from Eq. 9. To compute this numerically, we approximate the integral by summing over a discrete grid of $N \times N$ possible values for ξ and ϕ_a:

$$P(\psi|\delta) \approx \sum_{i=1}^{N} \sum_{j=1}^{N} p(\psi|\xi_i, \phi_{a,j}) \cdot p(\delta|\xi_i, \phi_{a,j})\, \Delta\xi\, \Delta\phi_a \tag{13}$$

where $\Delta\xi$ and $\Delta\phi_a$ are the steps in the discretization of the domain $[-\pi, \pi] \times [-\pi, \pi]$. For an illustration of the inference model, see Fig. 1D-F and Fig. 2 for the inference model for the different resource speeds (v_p).

Supplementary Note 2: Voluntary Payoff-Sharing Condition

In the Voluntary Payoff-Sharing condition, participants could decide whether to share their current payoff with group members. Sharing came at a small cost of 1 point per second. Out of 165 participants, only 12 (7.3%) never shared payoff information (2 in the slow-resource condition, 10 in the fast-resource condition). On average, participants initiated payoff sharing posterior mean = 6.58 and 90% HPDI = [5.60, 7.59] times in the fast-resource condition and posterior mean = 20.88 and 90% HPDI = [17.79, 23.96] times in the slow-resource condition (posterior estimates from a Bayesian Poisson regression). These sharing episodes corresponded to average sharing durations of posterior mean = 1.64 and 90% HPDI = [1.37, 1.91] minutes in the fast condition and posterior mean = 2.24 and 90% HPDI = [1.90, 2.55] minutes in the slow condition (posterior estimates from a Bayesian Beta regression), with the individual sharing for the longest period reaching 13.33 minutes.

The frequency of sharing decisions was positively associated with individual performance (Fig. 8A), and the total number of sharing decisions within a group was associated with the group's average score (Fig. 8B). Both associations were credibly stronger in the slow-resource condition. By contrast, the total time spent sharing was not reliably linked to higher scores at either the individual or group level.

We next examined payoff sharing at a more fine-grained scale by modeling the probability of sharing at one-second time steps. As expected, participants were more likely to share when

tracking the resource compared to searching for it, in both the fast (posterior mean contrast = 0.12 and 90% HPDI = [0.09, 0.14]) and slow (posterior mean contrast = 0.07 and 90% HPDI = [0.06, 0.08]) condition (Fig. 8C). Over time, sharing became more common in the fast condition when participants were tracking (posterior mean contrast = 0.06 and 90% HPDI = [0.04, 0.08]). Interestingly, in the slow condition participants shared payoff information more selectively compared to the fast condition, sharing less during tracking when they were far from the resource center, but equally when very close to it (Fig. 8D).

Supplementary Note 3: Rotation Direction Analysis

This supplementary analysis complements the rotation–magnitude results presented in the main text. Here we ask when participants reverse their rotation direction (i) in response to changes in detector values (Fig. 17) and (ii) as a function of the spatial configuration and quality of social information (Fig. 18).

Across payoff conditions, participants changed rotation direction more often in the fast-resource condition, consistent with adaptation to higher environmental volatility. Beyond this general effect, rotation direction changes followed a simple heuristic: Keep turning in the same direction when moving toward the resource center; reverse when moving away. As in the rotation–magnitude analysis, groups were less responsive to the detector than singletons, especially while approaching the resource (Fig. 17 left).

Social information was classified as *aligned with rotation* when it appeared in the FOV sector toward which the participant had just rotated. Figure 18 shows how the probability of a rotation direction change at time t depends on social information alignment at $t-1$. We also distinguished between two levels of social information quality, depending on whether the observed peer was closer to (good) or farther from (bad) the resource center than the focal individual.

In all group conditions, alignment with social information lowered the chance of a rotation direction change. However, when the social information was of low quality, the probability of a rotation direction change was substantially higher than when the participant was aligned with high-quality social information. The fact that participants were sensitive to the quality of social information when it was aligned with a rotation direction change in all payoff conditions might indicate that it was simple for them to evaluate the quality of social information in this case.

When social information was not aligned with the rotation direction, rotation direction change probability increased overall. However, its interaction with social information quality differed across payoff conditions. NP groups treated high- and low-quality social information similarly, whereas FP groups changed the rotation direction more often when misaligned with high-quality social information (see Fig. 18 right).

Taken together, decisions about rotation direction integrate private cues, the spatial alignment of social cues, and their relative quality. Payoff conditions govern sensitivity to social information quality. These patterns complement the findings reported in the main text.

Supplementary Note 4: Rotation Magnitude Model Random Effects Analysis

To assess the relationship between individual decision-making strategies and success, we extracted subject-level random effects (i.e., individual-specific weights) from the multilevel computational model of rotation magnitude. We then used these weights to predict behavioral outcomes. For tracking, we modeled tracking efficiency as a function of each individual's private and social weights. We modeled tracking efficiency separately for each weight using experimental conditions as fixed effects. The results are shown in Fig. 21 and discussed in the Results section of the main text. We repeated this analysis for rotation magnitude model of searching, using tracking time as the outcome. The results are shown in Fig. 22.

Supplementary Note 5: Rotation Magnitude Model Temporal Analysis

To investigate whether participants' movement strategies evolved over the course of the 15-minute experimental session, we extended the rotation magnitude computational models (described in main text). We introduced a binary temporal epoch feature to divide the data into the first half (0–7.5 min) and the second half (7.5–15 min) of the experiment. We then estimated the interactions between this temporal feature and all baseline parameters and decision weights, analyzing the search and tracking phases separately. The posterior estimates for these temporal contrasts (Second Half - First Half) are presented in Tables 13 and 14, and visualized in Figures 23 and 24. Taking the evidence together, we observed three main temporal shifts:

1. Overall decrease in baseline rotation. Across both tracking and searching phases, the overall baseline rotation magnitude decreased slightly but noticeably between the second and first halves of the study (tracking: posterior mean contrast = -0.11 and 90% HPDI = $[-0.15, -0.07]$; search: posterior mean contrast = -0.18 and 90% HPDI = $[-0.20, -0.17]$). This general reduction in rotation may indicate an increase in proficiency — as participants learned to avoid unnecessary, erratic movements — or a natural decrease in task engagement and energy over time. Interestingly, while the overall baseline dropped, rotation magnitude actually increased substantially in all group conditions relative to singletons during search in the second half of the study (posterior mean contrast NP-A = 0.20 and 90% HPDI = [0.18, 0.23]; posterior mean contrast VP-A = 0.23 and 90% HPDI = [0.21, 0.26]; posterior mean contrast FP-A = 0.27 and 90% HPDI = [0.24, 0.30]), highlighting an increased engagement with social features.

2. Adaptation of social strategies during search. A closer examination of the decision weights shows that participants systematically adjusted how they used social features while searching for the resource. Overall engagement with social features increased for fast environments over time. Towards the end of the study, participants in fast environments became more sensitive to social position and the interaction between position and social information quality (e.g., fast FP Position x Quality posterior mean contrast = 0.16 and 90% HPDI = [0.10, 0.22]).

In slow environments, groups became more selectively responsive to the interaction between observed participant movements and social information quality (slow VP Movement x Quality posterior mean contrast = 0.30 and 90% HPDI = [0.19, 0.41]; slow FP Movement x Quality posterior mean contrast = 0.12 and 90% HPDI = [0.00, 0.23]; slow NP Movement x Quality posterior mean contrast = 0.17 and 90% HPDI = [0.08, 0.25]). This suggests that as the session progressed, participants learned to better identify and rely on high-quality social cues to locate the resource.

3. Stability of tracking strategies. In contrast to the search, strategy changes during the tracking were minor. One coherent shift observed across almost all conditions was a slight decrease in sensitivity to private detector changes (e.g., slow A Detector Change posterior mean contrast = −0.14 and 90% HPDI = [−0.19, −0.08]). However, it is important to contextualize this effect: this decrease is approximately ten times weaker than the main effect of detector change (compare Main Text Fig. 4C with Fig. 23). This minor drop could be attributed to growing fatigue, as constantly monitoring and reacting to rapid detector changes is arguably the most cognitively demanding component of the task. Crucially, the social decision weights during tracking did not exhibit systematic or major shifts, indicating that the core strategies driving the performance differences between conditions were established rapidly and remained robust throughout the session.

Supplementary Note 6: Rotation Magnitude Model Additional Social Information Features

In approximately 31% of the observations, participants had two or more peers visible within their field of view (FOV) at the same time (see Table 12). In these instances, the simple spatial average of social cues might obscure important structural features of the observed collective, such as the number of visible peers (out-degree) or the alignment of their movements (cohesion). To test whether these higher-order structural features modulate social information use, we fitted an extended version of our rotation magnitude model specifically on the subset of data where participants observed two or more peers. We kept the overall model structure identical to the main analysis (see Main Text Fig. 4) but added interaction terms for out-degree (centered on 3) and Movement Cohesion. We defined cohesion quantitatively as the magnitude of the average social movement vector. Since the vast majority of our data falls into either the tracking in slow resource environment condition (≈ 80%) or the search in fast resource environment condition (≈ 80%), we focus our interpretation primarily on these contexts. Posterior estimates are presented in Fig. 25 (Tracking) and Fig. 26 (Search).

Overall, we did not observe a strong or consistent impact of the raw number of observed peers (Out-degree) on how social features were weighted. However, the cohesion of the observed peers noticeably modulated social information use. Across almost all conditions in both search and tracking, high movement cohesion among observed peers strongly reduced the participants' reliance on the group's spatial position (indicated by consistently negative Cohesion x Position interactions; see Figs. 25 and 26). Simultaneously, observing a cohesive group increased reliance on the group's average movement direction (positive Cohesion x Movement interactions). Fur-

thermore, during tracking, this shift toward movement alignment was particularly strong when the observed cohesive group was also experiencing high payoffs (positive Cohesion x Movement x Quality interactions, especially across FP/VP conditions). These results illustrate the complexity and adaptiveness of human social learning strategies providing initial evidence that people integrate additional properties of the observed groups of peers.

Impact of spatial distance on social information use. When constructing visibility network, we defined visual access strictly based on the FOV without imposing a hard cut-off for maximum visible distance. In a 3D virtual environment, the apparent size of a peer decreases with distance, which could theoretically reduce the salience and subsequent utilization of their social cues e.g.,[3,4]. To test whether physical distance devalues social cues in our task, we conducted a supplementary analysis. To ensure the methodologically clearest case and isolate the effect of distance without the confounding factor of aggregating multiple peers, we focused on search and subset the data to instances where participants had exactly one peer in their FOV. We then introduced peer distance as an interacting binary factor ("near" vs. "far") based on a median split of the observed peer distances. The posterior estimates of this model are shown on Fig. 27. Our analysis indicates that distant peers still impact participant's movement decisions across conditions. In fact, participants in the FP condition were substantially more impacted by the position and movement of distant peers than near peers. For example, in the fast resource environment, the posterior mean estimate for social position feature of a near peer was 0.47 and 90% HPDI = [0.36, 0.58], compared to 0.72 and 90% HPDI = [0.61, 0.84] for a far peer. While the effect sizes were smaller in the NP and VP conditions, participants in these groups also demonstrated that their movement decisions are impacted by the social cues from distant peers. These results provide evidence that rather than discounting distant peers, participants actively use their social cues – potentially because distant individuals provide information about previously unexplored areas of the environment, which is highly valuable during search. Consequently, physical distance does not inherently devalue social information in our task.

Supplementary Note 7: Interaction Between Private Sensitivity and Social Selectivity (ABM)

We used the agent-based model presented in the main text to explore how private and social information use interacted in shaping performance. Fig. 28 plots relative (to solitary agents) tracking time (left), tracking efficiency (center) and total score (right) for different degrees of selectivity to social information (on the x-axis) and sensitivity to the detector, i.e., changes in payoffs (on the y-axis). Increasing detector sensitivity allows agents to rotate more optimally, with a theoretical maximum detector sensitivity of 90 degrees. Blue cells corresponds to regions in parameter space where social agents perform worse than solitary agents, red cells correspond to regions where social agents perform better. The results show that the selectivity of social information plays a larger role when social agents are imperfect individual trackers. This is particularly evident in the lower part of Fig. 28 (middle panel), where agents with lower detector sensitivity tend to perform worse than singletons unless they are highly selective in their use of social information. Conversely, in the upper part of the plot - where agents have high detector

sensitivity - there is no indication that social agents fall below singleton performance, even at the lowest levels of selectivity. This suggests that when agents are near-perfect individual trackers, less selective use of social information still provides a comparative performance advantage over solitary conditions. They can closely track changes in payoffs, limiting the risks of maladaptive herding and information cascades. In addition, they may benefit more from copying, as others in the group are more likely to make optimal decisions and thus provide adaptive information.

References

[1] Wolpert, D. M. & Ghahramani, Z. Computational principles of movement neuroscience. *Nature Neuroscience* **3**, 1212–1217 (2000).

[2] Körding, K. P. & Wolpert, D. M. Bayesian integration in sensorimotor learning. *Nature* **427**, 244–247 (2004).

[3] Bastien, R. & Romanczuk, P. A model of collective behavior based purely on vision. *Science Advances* **6**, eaay0792 (2020).

[4] Mezey, D., Deffner, D., Kurvers, R. H. & Romanczuk, P. Visual social information use in collective foraging. *PLOS Computational Biology* **20**, e1012087 (2024).
