# Supplementary material for: Payoff selectivity drives collective intelligence during dynamic resource tracking in humans
Source: Nat Commun. 2026 Sep 23;17:10133. doi: 10.1038/s41467-026-77762-z (PMC13601515; doi:10.1038/s41467-026-77762-z)
Supplement: Supplementary file 2 [file 41467_2026_77762_MOESM2_ESM.pdf]

Description of Additional Supplementary Files

File Name: Supplementary Video 1
Description: Demonstration of resource movement (15× time-lapse). The black line shows the resource path; the red wireframe sphere (radius 40 m) represents the resource.

File Name: Supplementary Video 2
Description: Full session of a solitary (A) condition. View from a first-person perspective with the resource detector in the center of the screen.

File Name: Supplementary Video 3
Description: First 5 minutes of the voluntary payoff condition, reconstructed from the perspective of one participant. (3D avatar asset: Stylized Astronaut by PULSAR BYTES, used with permission).
