# Supplementary material for: Payoff selectivity drives collective intelligence during dynamic resource tracking in humans
Source: Nat Commun. 2026 Sep 23;17:10133. doi: 10.1038/s41467-026-77762-z (PMC13601515; doi:10.1038/s41467-026-77762-z)
Supplement: Supplementary file 3 [file 41467_2026_77762_MOESM6_ESM.pdf]

nature portfolio

Corresponding author(s): Valerii Chirkov

Last updated by author(s): Jul 3, 2026

Reporting Summary

Nature Portfolio wishes to improve the reproducibility of the work that we publish. This form provides structure for consistency and transparency in reporting. For further information on Nature Portfolio policies, see our Editorial Policies and the Editorial Policy Checklist.

Statistics

For all statistical analyses, confirm that the following items are present in the figure legend, table legend, main text, or Methods section.

n/a	Confirmed	
☐	☒	The exact sample size (*n*) for each experimental group/condition, given as a discrete number and unit of measurement
☐	☒	A statement on whether measurements were taken from distinct samples or whether the same sample was measured repeatedly
☐	☒	The statistical test(s) used AND whether they are one- or two-sided<br>*Only common tests should be described solely by name; describe more complex techniques in the Methods section.*
☐	☒	A description of all covariates tested
☐	☒	A description of any assumptions or corrections, such as tests of normality and adjustment for multiple comparisons
☐	☒	A full description of the statistical parameters including central tendency (e.g. means) or other basic estimates (e.g. regression coefficient) AND variation (e.g. standard deviation) or associated estimates of uncertainty (e.g. confidence intervals)
☐	☒	For null hypothesis testing, the test statistic (e.g. *F*, *t*, *r*) with confidence intervals, effect sizes, degrees of freedom and *P* value noted<br>*Give P values as exact values whenever suitable.*
☐	☒	For Bayesian analysis, information on the choice of priors and Markov chain Monte Carlo settings
☐	☒	For hierarchical and complex designs, identification of the appropriate level for tests and full reporting of outcomes
☐	☒	Estimates of effect sizes (e.g. Cohen's *d*, Pearson's *r*), indicating how they were calculated

Our web collection on statistics for biologists contains articles on many of the points above.

Software and code

Policy information about availability of computer code

Data collection	The 3D experiment was implemented using the Unity game engine (2021.3.14f1) and compiled for WebGL. The WebGL build was integrated and hosted using React JS (18.2.0), the React Unity WebGL module (9.4.0), and Next.js (13.1.51), served via an NGINX web server. Network synchronization and multiplayer server hosting were handled via PlayFab and the Mirror (2022.10.0) Unity package.
Data analysis	Data preprocessing, visibility network reconstruction, and agent-based modeling were performed using Python 3.9 with the mesa (2.2.4), numpy (1.24.2), pandas (1.5.3), xarray (2023.4.0), and NetworkX (3.0) libraries. Statistical and computational modeling was conducted in a Bayesian framework using Hamiltonian Monte Carlo in Stan, implemented in R version 4.5.3 using the brms (2.22.0), tidybayes (3.0.7), bayesplot (1.12.0), emmeans (1.11.1), and tidyverse (2.0.0) packages.

For manuscripts utilizing custom algorithms or software that are central to the research but not yet described in published literature, software must be made available to editors and reviewers. We strongly encourage code deposition in a community repository (e.g. GitHub). See the Nature Portfolio guidelines for submitting code & software for further information.

Data

Policy information about availability of data

All manuscripts must include a data availability statement. This statement should provide the following information, where applicable:

- Accession codes, unique identifiers, or web links for publicly available datasets
- A description of any restrictions on data availability
- For clinical datasets or third party data, please ensure that the statement adheres to our policy

The raw time series data, metadata, and processed tables of computed measures generated in this study have been deposited in the Open Science Framework (OSF) database under accession code https://doi.org/10.17605/OSF.IO/ZRHWT. All custom code used for the analysis and simulation in this study has been deposited in the OSF database under accession code https://doi.org/10.17605/OSF.IO/ZRHWT.

Research involving human participants, their data, or biological material

Policy information about studies with human participants or human data. See also policy information about sex, gender (identity/presentation), and sexual orientation and race, ethnicity and racism.

Reporting on sex and gender	The sample consisted of 239 females, 381 males, and 1 participant who preferred not to say. Sex and gender were not considered in the study design, and sex- and gender-based analyses were not performed, as the primary focus of the study was on general mechanisms of collective spatial search and tracking. Sex along with other demographic information was determined via self-reporting on the Prolific platform and is present in the anonymized open dataset.
Reporting on race, ethnicity, or other socially relevant groupings	Data on race, ethnicity, or other socially relevant groupings were not considered in the study design and no related analyses were performed, as the primary focus of this study was on general cognitive mechanisms of collective spatial search and tracking. General anonymized demographic information collected via self-reporting on the Prolific is included in the open data repository.
Population characteristics	The final sample's mean age was 33.56 years (SD = 10.17). We restricted the age of invited participants to 18-65, because we did not want to introduce additional sources of variation due to motor skills.
Recruitment	Participants were recruited through the online platform Prolific. Inclusion criteria required participants to be between 18-65 years old, fluent in English, have a minimum Prolific approval rate of 95%.
Ethics oversight	The research complies with all relevant ethical regulations and was approved by the Institutional Review Board of the Max Planck Institute for Human Development (approval number: A 2022-26). Informed consent was obtained from all participants.

Note that full information on the approval of the study protocol must also be provided in the manuscript.

Field-specific reporting

Please select the one below that is the best fit for your research. If you are not sure, read the appropriate sections before making your selection.

☐ Life sciences ☒ Behavioural & social sciences ☐ Ecological, evolutionary & environmental sciences

For a reference copy of the document with all sections, see nature.com/documents/nr-reporting-summary-flat.pdf

Behavioural & social sciences study design

All studies must disclose on these points even when the disclosure is negative.

Study description	This was a quantitative experimental study utilizing an online 3D immersive-reality task. The experiment employed a 4x2 between-subjects design, varying the type of social information available (Alone, No Payoff-Sharing, Voluntary Payoff-Sharing, and Full Payoff-Sharing) and the speed of the moving resource (slow or fast).
Research sample	The research sample consisted of adult participants recruited online via the Prolific platform. The final sample included 621 participants. The mean age was 33.56 years (SD = 10.17). The sex (self-reporting on the Prolific platform) breakdown was 239 females, 381 males, and 1 participant who preferred not to say. Participants were required to have a minimum Prolific approval rate of 95% and be fluent in English. The sample represents a broad online adult participant pool typical of crowdsourced behavioral experiments.
Sampling strategy	Participants were recruited using convenience sampling via the Prolific online platform. To maximize the number of complete groups formed, we simultaneously opened 50 participation slots per experimental condition on Prolific at a time. We aimed at collecting data from 15-20 groups of 5 participants per condition with equivalent number of participants in the solitary conditions. This target was chosen based on practical feasibility and alignment with sample sizes typical in recent immersive-reality collective behavioral experiments. This sample size was deemed sufficient to provide enough individual- and group-level observations for the Bayesian multilevel regression models to reliably estimate posterior distributions and detect behavioral differences across the experimental conditions.

Data collection	Data was collected completely online. Participants used their own personal computers and web browsers to access the experiment, which was developed using the Unity game engine (WebGL). The PlayFab game server automatically recorded participants' positional coordinates, rotations, and scores at a sampling rate of 10 Hz. Because the study was conducted entirely online via participants' personal devices, no researchers or third parties were present during data collection.
Timing	Data collection was conducted over three periods in the year 2023. The first half of the dataset was collected in April 11-12 (Alone, No Payoff-Sharing, and Full Payoff-Sharing conditions) and May 3 (Voluntary Payoff-Sharing condition). The remaining half of the data across all experimental conditions was collected from November 13-17.
Data exclusions	Yes, data were excluded based on pre-established criteria. From an initial pool of 796 participants, the following exclusions were made: participants who did not complete the experiment (n=21) ; participants with a low frame rate (> 5% below target) causing faulty rendering (n=27) ; participants who did not move for more than 25% of the experiment (n=29) ; participants who could not be matched into a group within 10 minutes (n=59) ; and participants assigned to groups with more than one invalid participant (n=39). This resulted in the final analyzed sample of 621 participants.
Non-participation	All cases of dropouts or incomplete participation are accounted for in the pre-established data exclusions detailed above. No additional participants dropped out or declined participation.
Randomization	Participants were pseudo-randomly allocated to conditions based on the time they logged into the Prolific platform and joined an available session.

Reporting for specific materials, systems and methods

We require information from authors about some types of materials, experimental systems and methods used in many studies. Here, indicate whether each material, system or method listed is relevant to your study. If you are not sure if a list item applies to your research, read the appropriate section before selecting a response.

Materials & experimental systems

n/a	Involved in the study
☒	☐ Antibodies
☒	☐ Eukaryotic cell lines
☒	☐ Palaeontology and archaeology
☒	☐ Animals and other organisms
☒	☐ Clinical data
☒	☐ Dual use research of concern
☒	☐ Plants

Methods

n/a	Involved in the study
☒	☐ ChIP-seq
☒	☐ Flow cytometry
☒	☐ MRI-based neuroimaging

Plants

Seed stocks	*Report on the source of all seed stocks or other plant material used. If applicable, state the seed stock centre and catalogue number. If plant specimens were collected from the field, describe the collection location, date and sampling procedures.*
Novel plant genotypes	*Describe the methods by which all novel plant genotypes were produced. This includes those generated by transgenic approaches, gene editing, chemical/radiation-based mutagenesis and hybridization. For transgenic lines, describe the transformation method, the number of independent lines analyzed and the generation upon which experiments were performed. For gene-edited lines, describe the editor used, the endogenous sequence targeted for editing, the targeting guide RNA sequence (if applicable) and how the editor was applied.*
Authentication	*Describe any authentication procedures for each seed stock used or novel genotype generated. Describe any experiments used to assess the effect of a mutation and, where applicable, how potential secondary effects (e.g. second site T-DNA insertions, mosiacism, off-target gene editing) were examined.*
