# Supplementary material for: Payoff selectivity drives collective intelligence during dynamic resource tracking in humans
Source: Nat Commun. 2026 Sep 23;17:10133. doi: 10.1038/s41467-026-77762-z (PMC13601515; doi:10.1038/s41467-026-77762-z)
Supplement: Supplementary file 4 [file 41467_2026_77762_MOESM7_ESM.pdf]

nature portfolio

Peer Review File

Payoff selectivity drives collective intelligence during dynamic resource tracking in humans

Corresponding Author: Mr Valerii Chirkov

This file contains all reviewer reports in order by version, followed by all author rebuttals in order by version.

Version 0:

Reviewer comments:

Reviewer #1

(Remarks to the Author)
This paper presents an impressive 3D immersive-reality experiment where participants search for a moving resource under naturalistic visuospatial constraints. However, I have significant concerns about the clarity of the core theoretical contribution. Specifically, I found key aspects of the authors' central claims to be underdeveloped, making it difficult to evaluate the extent to which they are warranted by the evidence. First, while the authors critique prior work for using "simplified paradigms," their own task remains quite abstract (sparse environment; veridical, unidimensional payoff signal). The introduction needs a more focused argument for the theoretical gap this particular design addresses beyond general appeals to naturalism. Second, and most critically, the paper's central construct—"payoff selectivity"—is never defined despite being featured in the title and key claims. This concept needs explicit theoretical development in the introduction, clear operationalization in the methods, and more careful discussion of how the evidence supports claims about its causal role. With substantial revision addressing these conceptual issues, this could make an important contribution. However, currently the theoretical contribution feels diffuse.

(1) The task used in this paper is impressive, and the authors' efforts to make sense of players' exploration in this 3D environment is compelling and rigorous. But, beyond the obvious technical merits of this paper, I find it hard to pin down the scientific advance being made here. The introduction is quite dense, focusing on how prior work does not fully capture social learning dynamics in naturalistic environments because they use simplified decisions and non-immersive tasks that lack real-world visuospatial dynamics. I think the authors' efforts to bring naturalism back into their task are admirable. However, the task is still quite simplified—while players navigate a 3D environment, the environment itself is mostly bare, and players are still fed a veridical, unidimensional "payoff" signal. While the visuospatial dynamics of this task are indeed interesting, they are still vastly simplified compared to the challenge of figuring out what to value in a messy, multifaceted world where rewards are often opaque, as in the cultural processes that have led to the development of many real-world food processing techniques (e.g., Henrich & Henrich, 2010). The social environment is also quite simplified—participants are placed in a barren environment with up to four other strangers, and the richest form of communication available between them is directly sharing payoffs.

To be clear, I don't think any of these design decisions are problematic—the process by which participants calibrate what to look at, what to explore, and what to communicate is indeed rich and interesting. The problem is that this paper claims to address gaps in more simplified paradigms, while the task remains quite simplified and stylized. This paper needs a more specific argument for what specific insights the 3D environment enables that couldn't be achieved otherwise.

(2) What exactly is payoff selectivity? This term is prominently featured in the title and in the key scientific claims of the paper, but it is never defined. The closest I could find to an explanation of this concept is tucked into the agent-based modeling section—where "higher selectivity indicates that agents only use social information if others were doing better than they were at the time". But this term needs to be defined clearly and prominently; the introduction should be revised to provide the theoretical motivation for looking at this construct; and the computational modeling results section, in particular, needs to be revised to clarify how it provides evidence that "payoff selectivity was the key factor determining collective outcomes".

(3) I found the Voluntary Payoff Sharing condition particularly interesting. This condition seems like a notable departure from similar, recent work using game environments (e.g., Hawkins et al., 2023; Wu et al., 2025), and the decisions about whether

and when to communicate is an interesting one. However, the discussion of this condition feels underdeveloped throughout the paper. For example, it would be helpful to know:

a. What's the best possible performance attainable in this condition? Is there any normative baseline that participant's behavior could be compared to, to establish whether they are underutilizing information sharing vs. appropriately weighing the costs and benefits?
b. I wonder if performance in the VP condition is artificially deflated because participants have to pay a small cost to communicate. Why include this cost at all? Would performance be quite this low if participants could choose to share cost information freely (barring the small opportunity cost of activating information sharing)?

(Remarks on code availability)
I have checked the repository, but have not tried downloading and running the code locally. At first glance, the project files are well-organized, and the authors have adhered to certain best practices in computational reproducibility, including sharing a requirements.txt file with the exact Python environment needed to reproduce Python-based analyses.

It would be helpful to add a README with minimal instructions on how to reproduce key analyses (e.g., where should one go to reproduce the result in Figure 2?), and if the authors similarly packaged the environment needed to reproduce R-based analyses.

Reviewer #2

(Remarks to the Author)
This is a well-crafted manuscript investigating social responses to collective adaptation and complex social learning through a social virtual 3D immersive-reality experiment involving 621 participants seeking resources under different levels of participation (solitary, groups), movement speed, and access to reward information (no payoff/NP, voluntary payoff/VP, and full payoff/FP). The authors' computational, technical, and visualization skills are evident throughout the manuscript, making it highly engaging for the reader. The careful work undertaken in developing and conducting the experiment, visualizing the results, producing video material, and presenting diagrams captures the reader's attention and sets high expectations regarding the research content and the anticipated impact of the findings. Having developed such high expectations, I note that the main limitation of the manuscript concerns its currently limited interdisciplinary positioning in terms of how the information is communicated and structured.

As a reader and reviewer with a different background from the fields the manuscript addresses, I am left wondering whether the manuscript's contribution statement (Contrasting earlier work, participants in groups outperformed solitary individuals, but only when payoff information was available") represents an advance of significant importance. I also question the realism claimed by the manuscript in the simulation, considering that the variables examined in the experiment (position: continuous variable, speed: binary variable, aggregation/collectivity level: categorical variable, payoff state: categorical variable) could be counted almost on one hand. In particular, the visibility criterion used creates a setting that appears to contradict the realism claim, as it is based on a fundamental algebraic formulation in a two-dimensional space, without a clear indication of whether proximity/apparent size is considered (see comment below).

Moreover, as a reader of a high-impact interdisciplinary journal, I am cautious when reading that the manuscript's realism is highlighted by comparison with "Previous theoretical work and experiments with fish". I share these reflections because the statement "We therefore predicted that participants in the Voluntary Payoff-Sharing condition would pay a small cost to share payoff information, and that their performance would fall in between the performance of participants in the No Payoff-Sharing and Full Payoff-Sharing conditions", as well as the manuscript's title, resonates strongly with frameworks I am familiar from economics (e.g., monopoly: incomplete-individualized information vs. perfect competition: full information), economic geography (e.g., Allan Pred's model, see comment below), and network science (see comment on evolutionary game-theoretic information in Easley and Kleinberg, below), which makes me wonder how the manuscript sufficiently advances contemporary research findings.

In this context, and to the extent that I represent a non-specialist readership in the manuscript's topic (whose engagement could multiply the article's potential impact) I encourage the authors to consider the possibility of revisiting the manuscript's motivation, contribution, and conclusions to better align with the broadened multidisciplinary scope implied by the manuscript's title and to appeal more effectively to a wider readership of the journal. For instance, while I read with particular interest the observations: "Participants who collected more points paid little visual attention to others (low out-degree), but received a lot of visual attention from others (high in-degree)…" (l.157–161) and "Discoverers who shared their payoffs received more visual attention and others increased their outward attention" (l.157–161), I notice that these are not explicitly stated as conclusions. Observations regarding participants' strategy-shifting conditions may warrant additional emphasis, and I encourage the authors to focus on them.

Without wishing to overextend my comments, I see promising potential in the manuscript, particularly in the opportunity to further embrace an interdisciplinary positioning.
Detailed Comments

- The manuscript could benefit from a typical structure (introduction, literature review, methods and data, results and discussion, conclusion). For instance, the first paragraph stands for an introduction/compact literature review on concepts, the second paragraph for an introduction/compact literature review on methods/techniques, the third paragraph for a methods section, etc.

- The first two paragraphs (l.1-24) in the introduction would benefit from a real-world paradigm of how individuals “arbitrate between dynamically changing social and non-social features” (l.13-14) and what, e.g., "social features” (l.14) may be.
- In l.25, it is not clear whether the participants are human entities or are simulated agents. A multidisciplinary/non-specialized reader should seek this information within the manuscript (l.60, l.469) and supplementary information, a fact that can harm the manuscript’s readability. The manuscript would benefit from such a clarification/explanation to acclimatize the unfamiliar reader
- The reward/payoff model used for the simulation (l.51) appears to be centrality-based ("the closer participants were to its (the resource’s) center, the more points they collected”). I believe that the manuscript should benefit from a methodological explanation about the choice of this barycentric/core model, instead of, e.g., a payoff model proportional to the resource area scanned by the avatar.
- Stating that “we predicted that search and tracking would be more efficient for collectives than for solitary individuals” (l.71) may not, on its own, constitute a novel research contribution of this manuscript. The benefits of collectivity, agglomeration, or clustering are well-documented across multiple disciplines under concepts such as “economies of agglomeration”, “economies of concentration”, or “network effects”. In this context, the observed ordering NP < VP < FP, highlighted as a major finding of the manuscript, might appear somewhat expected within a broader theoretical framework spanning various fields. For example, elements of the trade-offs among (i) the amount of available information, (ii) the skills required to process that information, and (iii) the optimal location (payoff) can be traced in spatial economics theories, including classic behavioral and location models such as Allan Pred’s (1970s) Behavior and Location: Foundations for a Geographic and Dynamic Location Theory. Similarly, models of spatial clustering, as a trade-off between transportation costs and gains in firms’ information availability, draw on economic geography frameworks dating back to Alfred Weber’s Theory of the Location of Industries (1929, University of Chicago Press). Furthermore, Easley and Kleinberg (2010) in Networks, Crowds, and Markets: Reasoning About a Highly Connected World (Vol. 1, Cambridge University Press) provide evolutionary game-theoretic insights regarding collective dynamics, associated trade-offs, and payoffs from the perspectives of network science and network economics. In general, enough models across disciplines have examined agglomeration and network effects. Accordingly, while the NP < VP < FP finding (l.81–82) is interesting, it may not, by itself, constitute a fully sufficient contribution without further contextualization relative to the existing theoretical framework.
- The statement “simulation work has shown that unconditional sharing of payoff information can evolve in collective search, acting as a recruitment signal that facilitates the self-organization of groups (Tump et al., 2019)” would benefit from an alternative phrasing denoting that the findings verify the Tump et al. (2019) approach instead of showing that …
- It is not clear under which criteria groups of five (l.466) were formed to proxy social collectivity. Under what theoretical/literature context/assumption was this adopted? Please support with references.
- Using the term 'visibility network' (l.506) in a manuscript claiming a multidisciplinary space of publication should be done after defining it. Beyond visibility networks in social sciences, the term is also widely used in time series graph analysis, as introduced by Lacasa et al. (2008) (https://www.pnas.org/doi/abs/10.1073/pnas.0709247105). Using the term without a proper conceptualization suffers from disciplinary constraints.
- The visibility rule described in l.511–512 appears somewhat based on intuition and may benefit from further justification. First, the forward-facing field of view (FOV) criterion should be supported with appropriate references. Second, the visibility criterion in a 2D space is typically defined as $|\theta_{i\rightarrow j} - \theta_i| < FOV/2$. In the manuscript, the choice of FOV = 90 degrees (rather than, for example, 120 degrees; see e.g., [link]) is neither fully justified nor explained in context, nor is it supported by citations. In this regard, it would be helpful to clarify how sensitive the results are to the choice of FOV = 90 degrees versus 120 degrees.
Finally, since the experiment is conducted in a virtual space, not accounting for the apparent size of objects (i.e., based on their distance from the observer) may limit the completeness of the visibility network modeling. It could be useful to consider a second visibility criterion, such as $|\theta_{i\rightarrow j} - \theta_i| < FOV/2$ and $distance(i,j) \leq D_{max}$, to more fully capture participants’ perceptual constraints in the simulated environment.
- In general, all equations in the manuscript lack citations. This omission gives the impression that they are novel equations introduced in this paper, an assumption that necessitates providing their proofs in the supplementary information file. Conversely, they need to be supported by references.

(Remarks on code availability)

Reviewer #3

(Remarks to the Author)
The paper presents a collective search experiment that finds that people are able to flexibly integrate individual and social sources of information. Behavioral results are accompanied by computational modeling of the obtained results and an agent-based model to test boundary conditions. It's a well-designed study, which is interesting both for the obtained results and a novel experimental paradigm.

My concerns are mostly about how information is presented, and I will go more or less in order.

1. Hypothesis 1 is well motivated by prior literature. Hypothesis 2, however, creates some tension with the Hawkins et al. (2023) study mentioned in the introduction, which found that social information was sufficient for collective search. HP 2 states that having payoff information will lead to better performance, but it does not clearly explain why. Is it because this study has a more realistic setting compared to the Hawkins et al. setup?

2. Hypothesis 3 feels exploratory rather than theory-driven and, in fact, doesn't appear in preregistration. While the authors cite Tump et al. (2019) showing that payoff sharing can evolve, it doesn't explain why we should expect this to happen in a

15-minute one-shot experiment with individual payoffs and information sharing costs. The authors should either: (a) justify the introduction of the voluntary sharing condition and their predictions in the specific paradigm used, or (b) frame this condition as exploratory but still explain the possible mechanisms of sharing in this task.

3. "Visibility networks" are first mentioned in line 89, but this term is not explained. Please provide an intuitive explanation when first introducing technical concepts.

4. The fact that groups were a size of 5 only appears implicitly in Figure 1 and the Methods. It would help to mention this more explicitly early on, for example, around lines 54-60.

5. Social features are defined based on self vs the average of others (position, movement direction). However, averaging removes information about social evidence strength. For example, I would imagine people are more likely to follow a cluster of 4 others moving in the same direction than a group of 2, but averaging their positions/direction would not capture this difference. The authors should either: (a) justify why averaging is appropriate, or (b) include additional features that capture the number of visible others, their coherence, etc.

6. Figure 4: I believe the legend should say NP, VP, FP, and not NS, VS, AS?

7. There appears to be considerable variability in individual performance in all conditions (figures S4, S5), and the main text mentions that people vary in their reliance on different cues, but these individual differences aren't analyzed in detail. I understand it's difficult to fit everything into 1 paper, but it would be nice to see a more focused paragraph on this issue, or an additional figure (e.g., computational model weights across individuals) in the main text or the Supplement.

8. Similarly, the paper doesn't examine temporal dynamics within the 15-minute session. Is there any evidence of improvement over the session? Either individually or with respect to group strategies? The authors could divide the data into epochs, or at least examine the beginning and the end of the session. This could tell us whether conditions differ because there are stable differences in people's strategies depending on available cues, or if they differ due to distinct effects of cue availability on collective learning. The changes over time are particularly important for the voluntary sharing condition.

9. The paper does many things - introduce a more complex task (continuous tracking in 3D), a notion of payoff selectivity, a result that NP groups do worse than individuals. It would help to make it clearer (explicitly stated) what the authors consider to be their main contribution/novelty.

10. I have concerns about framing the task that relies solely on individual payoffs as 'collective intelligence' (including in the title). Much of the analysis focuses on how individuals use various cues to improve their performance, and so it is definitely a paper about how individuals can do better (but sometimes worse) in group contexts. Collective intelligence would require that there are emergent group-level processes or outcomes that are not reducible to individuals (e.g., Woolley et al., 2010). The authors suggest that, for example, groups can stay on target longer because of how they learn from and follow each other (as a kind of social amplification of search patterns), but this is not highlighted or quantified as an emergent collective effect, distinct from individuals just benefiting from information collected by others. I recommend either (a) reframing the paper as being about 'adaptive social learning in groups' or (b) adding analyses that genuinely demonstrate collective-level phenomena.

(Remarks on code availability)
I opened the code link and saw the folders with files. I was able to download each file individually, but not the zip of everything ({"code": 503, "message": "Unable to connect to auth sever"}).
There's no README, so it's daunting to try to understand what to run and in which order.

Version 1:

Reviewer comments:

Reviewer #1

(Remarks to the Author)
In this revised manuscript, the authors have made the following changes in response to my comments:

(1) Reworked the introduction to clarify the theoretical claims of the paper, particularly by specifying in what ways the task is "naturalistic"
(2) Reworked the introduction, discussion, and results to define the concept of "payoff selectivity" and explicitly tie it to analyses
(3) Justified the small additional cost in the VP condition and run additional analyses to check whether performance is artificially deflated in this condition because of this cost

Overall, I am impressed by the latest round of revisions and feel that all of my comments have been addressed. I particularly appreciated the new explanation of "naturalism"; I expect that I'll refer back to and cite the authors' explanation here often in future reviews.

My one suggestion is that the discussion of payoff selectivity in LL.19-41 is a bit dense. Looking through the analyses that

now explicitly refer to payoff selectivity, it seems that what the authors *actually* looked at is whether people can adaptively tune their information use based on others' payoffs, particularly in more complex environments where they don't have all the information available to them at once (compared to, say, a birds'-eye view) and where others may not always disclose their payoffs. It would help to simplify this section a bit so that the research question comes through more clearly—as a reader, my sense was that the list of payoff-based processes in LL.29-41 is a bit too complex, so it's hard to tell what's most important and relevant to the paper here. (For example, it's interesting to consider that others' payoffs may not always directly inform the best action to take—as in the case of Witt et al., 2024, where partners' preferences can differ from our own. But that doesn't seem relevant to the current paper, so it threw me for a loop.)

(Remarks on code availability)

Reviewer #2

(Remarks to the Author)
The authors have addressed my concerns with seriousness and professionalism. For example, I appreciate that each of my suggestions led to revisions or modifications in the text, resulting in improved readability and presentation. For instance, I welcome the additional discussions regarding the integration mechanisms of fish or mushrooms, the clarifications on “naturalistic” versus “real-world” or realistic scenarios, the definition of visibility networks in this research context, and the selection of forward-facing fields of view.

However, my remaining concern stems from comment numbered “Point P2,” which pertains to group size. Although the authors have provided a convincing response regarding the specific framework of Reviewer Point P2.8, I still have reservations about the sensitivity of the overall research design on the outcomes. In particular, while I feel the design is over-parameterized, I would like to see a sensitivity assessment regarding group size and a more comprehensive discussion about the sensitivity of the research parameters overall. As a non-specialist reader, I find that articles published in NCOMMS should not be overly case-sensitive (such papers would, e.g., be better suited for SciRep) and should present generalizable outcomes.

Therefore, I kindly ask the authors to provide further evidence supporting the group size sensitivity and generality of their approach and to better highlight their contributions for a multidisciplinary audience, which is the target readership of this journal.

(Remarks on code availability)

Reviewer #3

(Remarks to the Author)
The authors have significantly improved the manuscript. I find that most of my concerns have been addressed by either clarification or additional analysis. It was especially interesting to see new results that take into account additional social features and temporal dynamics - I think they both strengthen the current paper and open avenues for further research. I also appreciated an explicit discussion of different definitions of collective intelligence and how they relate to the paper.

I have two remaining concerns, both of which have to do with the conceptual clarity of the Introduction. I would recommend revising these before accepting for publication.

1. Voluntary Payoff Sharing rationale (lines 110-116): The term "unconditional sharing" from Tump et al. means sharing freely, but as a term feels in tension with the contrast between "voluntary" and "full" (that is, intuitively I would see "full sharing" as "unconditional" as "voluntary" is conditional on the individual's choice).
More importantly, the prediction that voluntary sharing would fall "between" NP and FP conditions isn't well justified. I could well imagine two other outcomes: 1) VP could fall below both NP and FP, for example, due to cost (in points) or cognitive cost (trying to decide whether to share); 2) VP could fall above FP because strategic sharing might be more informative. If the authors insist on making a specific prediction for VP, they would need to justify it better. However, I think it would be better (more consistent with the condition's exploratory nature) to frame it as an open empirical question with 3 possible outcomes.

2. The last paragraph of the Introduction (lines 117-137) is the most crucial in delineating the paper's contribution. It should clearly define the key concept, "payoff selectivity," as soon as it is introduced. Then, the paragraph would be better structured as a clear explanation of how individual cognition, collective behavior, and environmental variables are mechanistically linked. Currently, it reads more like a results summary (a list) than a contribution statement (clarification of how everything fits together). Consider restructuring to: (a) define payoff selectivity explicitly, (b) show the chain of logic connecting individual mechanisms -> collective patterns -> environmental contingencies (back to individual mechanisms?), and (c) clarify how this multi-level integration is your novel contribution.

(Remarks on code availability)
I installed and ran the code with small issues:
- I think it is better to specify Python version in the venv line: `python3.9 -m venv venv`

- The tables package requires HDF5 to be installed
- Add `pip install ipykernel' to the commands in step 3

The rest run successfully and replicated.

Open Access This Peer Review File is licensed under a Creative Commons Attribution 4.0 International License, which permits use, sharing, adaptation, distribution and reproduction in any medium or format, as long as you give appropriate credit to the original author(s) and the source, provide a link to the Creative Commons license, and indicate if changes were made.
In cases where reviewers are anonymous, credit should be given to 'Anonymous Referee' and the source.
The images or other third party material in this Peer Review File are included in the article's Creative Commons license, unless indicated otherwise in a credit line to the material. If material is not included in the article's Creative Commons license and your intended use is not permitted by statutory regulation or exceeds the permitted use, you will need to obtain permission directly from the copyright holder.
To view a copy of this license, visit https://creativecommons.org/licenses/by/4.0/

Reviewer 1

Reviewer Point P 1.1 — This paper presents an impressive 3D immersive-reality experiment where participants search for a moving resource under naturalistic visuospatial constraints. However, I have significant concerns about the clarity of the core theoretical contribution. Specifically, I found key aspects of the authors' central claims to be underdeveloped, making it difficult to evaluate the extent to which they are warranted by the evidence. First, while the authors critique prior work for using "simplified paradigms," their own task remains quite abstract (sparse environment; veridical, unidimensional payoff signal). The introduction needs a more focused argument for the theoretical gap this particular design addresses beyond general appeals to naturalism. Second, and most critically, the paper's central construct—"payoff selectivity"—is never defined despite being featured in the title and key claims. This concept needs explicit theoretical development in the introduction, clear operationalization in the methods, and more careful discussion of how the evidence supports claims about its causal role. With substantial revision addressing these conceptual issues, this could make an important contribution. However, currently the theoretical contribution feels diffuse.

Reply: We thank the reviewer for their overall positive evaluation of our task and analyses and for pointing out the lack of conceptual clarity in the previous version of our manuscript. As explained in detail below, we have (1) considerably reworked the theoretical rationale of our study, clarifying the core theoretical contributions, (2) better explained what we mean by "naturalistic" experiments, as well as (3) clearly defined the term "payoff selectivity" and explained how the computational and agent-based models jointly demonstrate its causal role in the emergence of collective intelligence.

Reviewer Point P 1.2 — The task used in this paper is impressive, and the authors' efforts to make sense of players' exploration in this 3D environment is compelling and rigorous. But, beyond the obvious technical merits of this paper, I find it hard to pin down the scientific advance being made here. The introduction is quite dense, focusing on how prior work does not fully capture social learning dynamics in naturalistic environments because they use simplified decisions and non-immersive tasks that lack real-world visuospatial dynamics. I think the authors' efforts to bring naturalism back into their task are admirable. However, the task is still quite simplified—while players navigate a 3D environment, the environment itself is mostly bare, and players are still fed a veridical, unidimensional "payoff" signal. While the visuospatial dynamics of this task are indeed interesting, they are still vastly simplified compared to the challenge of figuring out what to value in a messy, multifaceted world where rewards are often opaque, as in the cultural processes that have led to the development of many real-world food processing techniques (e.g., Henrich & Henrich, 2010). The social environment is also quite simplified—participants are placed in a barren environment with up to four other strangers, and the richest form of communication available between them is directly sharing payoffs. To be clear, I don't think any of these design decisions are problematic—the process by which participants calibrate what to look at, what to explore, and what to communicate is indeed rich and interesting. The problem is that this paper claims to address gaps in more simplified paradigms, while the task remains quite simplified and stylized. This paper needs a more specific argument for what specific insights the 3D environment enables that couldn't be achieved otherwise.

Reply: We fully agree with the reviewer that developing more naturalistic experimental paradigms should not be a research goal in itself, unless the added complexity provides additional theoretical insights. By "naturalistic", we thus do not mean scenarios that are simply more complex or that appear prima facie more similar to the real world. Instead, we refer to heterogeneous and dynamically changing environments characterized by temporal and spatial autocorrelation as well as perceptual and spatial constraints (Beauchamp, 2008; Rahmani et al., 2020); in such environments, the cues participants encounter and base their decisions on are not directly imposed by the experimenter but are shaped by one's own previous actions as well as the actions of others (Brunswik, 1955; Fawcett et al., 2014). Because it has been argued that the ecological validity of such personal and social cues (i.e., the degree to which they reflect statistical patterns in the world) also determines the ecological validity of the experiment itself (Kihlstrom, 2021), naturalistic approaches in this stricter sense also help us to close the gap between experimental work and real-world behavior. Following from this understanding of "naturalism", we want to emphasize that our goal was not to include as much real-world complexity as possible (our task was still highly stylized in many ways), but to include key features of real-world decision environments that fundamentally shape the costs and benefits of social information use, but have been largely omitted in previous paradigms. These features include (1) perceptual and spatial constraints, (2) temporal and spatial autocorrelation, and (3) endogenous choice ecologies. We agree that our previous contrast between "simplified" and "naturalistic" experiments was too stark. We have now substantially re-written the respective sections in the manuscript (LL. 51–58), emphasizing how our task differs from previous approaches and why this might be more reflective of decision-making outside of the lab and, hence, more "naturalistic".

Lastly, we totally agree that our paradigm does not directly speak to the multi-generational cultural evolutionary processes underlying, for example, food taboos or processing techniques (e.g., Henrich & Henrich, 2010). These processes are undoubtedly key for our understanding of human adaptability, but would require a different paradigm. We now mention potential multi-session setups as an exciting opportunity for further research into multi-generational cultural adaptation, citing the Henrich & Henrich, 2010, study as a prime real-world example (L. 503).

Reviewer Point P 1.3 — What exactly is payoff selectivity? This term is prominently featured in the title and in the key scientific claims of the paper, but it is never defined. The closest I could find to an explanation of this concept is tucked into the agent-based modeling section—where "higher selectivity indicates that agents only use social information if others were doing better than they were at the time". But this term needs to be defined clearly and prominently; the introduction should be revised to provide the theoretical motivation for looking at this construct; and the computational modeling results section, in particular, needs to be revised to clarify how it provides evidence that "payoff selectivity was the key factor determining collective outcomes".

Reply: We apologize for not clearly defining this key term before and thank the reviewer for pointing out this omission. By payoff selectivity we mean "the selective tuning of attentional and behavioral responses depending on others' current payoffs", a definition now also provided in a prominent place in the introduction (LL. 19–41). In other words, payoff selectivity means that people do not attend to or follow social cues (e.g., the position and movement direction of others) indiscriminately, but proportional to how well observed others are currently doing. In addition to including this definition, we have thoroughly revised the introduction to outline different payoff-based processes discussed in the literature and, thereby, provide a clearer theoretical motivation for our study. Moreover, payoff selectivity (in either visual attention or social information use) plays a crucial role across several analyses. We agree

that this was not clear enough before, so we now explicitly mention where payoff selectivity is studied throughout the manuscript (e.g., L. 222, L. 256, L. 289, L. 319, L. 337).

Most importantly, both the computational and the agent-based models include payoff selectivity as unique parameters which tune the influence of social features depending on observed payoffs. This allowed us to directly test the influence of payoff selectivity on participants' and agents' behavior. The computational model demonstrates such selectivity empirically (Fig. 4) and shows that it is associated with performance (Figs S21, S22); the agent-based model provides causal evidence that manipulating the selectivity of agents influences collective performance, with high levels of selectivity being required to outperform solitary individuals, i.e., for collective intelligence to emerge (Fig. 5).

Reviewer Point P 1.4 — I found the Voluntary Payoff Sharing condition particularly interesting. This condition seems like a notable departure from similar, recent work using game environments (e.g., Hawkins et al., 2023; Wu et al., 2025), and the decisions about whether and when to communicate is an interesting one. However, the discussion of this condition feels underdeveloped throughout the paper. For example, it would be helpful to know:

a. What's the best possible performance attainable in this condition? Is there any normative baseline that participant's behavior could be compared to, to establish whether they are underutilizing information sharing vs. appropriately weighing the costs and benefits?

b. I wonder if performance in the VP condition is artificially deflated because participants have to pay a small cost to communicate. Why include this cost at all? Would performance be quite this low if participants could choose to share cost information freely (barring the small opportunity cost of activating information sharing)?

Reply: We thank the reviewer for highlighting the Voluntary Payoff-Sharing (VP) condition and for raising these excellent points. We have expanded our discussion of this condition in the revised manuscript to address both the impact of the cost and the complexity of optimal signaling.

Regarding point (**a**) and a normative baseline for optimal sharing: determining the strictly optimal amount of signaling in our paradigm is highly non-trivial because the value of social information is inherently context-dependent. For instance, if a participant is far from the resource while others are tracking it, this participant's signal has little value. Conversely, if all group members are searching, signaling a lack of resource detection can help the group avoid unsuccessful areas. Given the dynamic and spatial nature of our task, formally deriving an exact normative baseline for communication is beyond the scope of this manuscript—if possible at all.

Regarding point (**b**) and whether performance in the VP condition is artificially deflated by the signaling cost: to test this, we re-ran our performance models using the "gross score" (i.e., adding the incurred signaling costs back to the participants' final scores). These results are now presented in the new Table S3. Comparing Table S2 (net score) with Table S3 (gross score) shows that adding the costs slightly improves performance in the VP condition, but does not alter our overall findings. To put the cost into perspective: the performance gap between the Full Payoff-Sharing (FP) and No Payoff-Sharing (NP) conditions is approximately $2,545$ points (fast resource) and $3,950$ points (slow resource). By contrast, the *maximum* possible signaling cost a participant could incur—if they shared continuously for the entire 15-minute experiment—is only 900 points (15 mins $\times$ 60 secs $\times$ 1 point/sec). Therefore, even if participants paid the maximum possible cost to constantly broadcast their payoff, the benefits of full information sharing would on average still far outweigh the costs. We have clarified this cost-benefit relationship in the Results section (LL. 154–159).

Why include the cost at all? We introduced this (small but still significant) cost to create a social dilemma. By imposing an individual cost for sharing information, the VP condition creates scenarios similar to the free-rider problem and the tragedy of the commons, where individuals need to pay a small cost to benefit others. We now elaborate on this theoretical motivation in the Discussion (LL. 491–496).

Finally, we completely agree that understanding the specific strategies people use to optimize signaling under these dilemmas is a fascinating direction for future research. In the discussion, we now mention exciting follow-up experiments focused entirely on the evolution of optimal signaling strategies over extended, multi-session interactions, including the systematic manipulation of signaling costs (LL. 496–503).

Remarks on code availability

Reviewer Point P 1.5 — I have checked the repository, but have not tried downloading and running the code locally. At first glance, the project files are well-organized, and the authors have adhered to certain best practices in computational reproducibility, including sharing a requirements.txt file with the exact Python environment needed to reproduce Python-based analyses.

It would be helpful to add a README with minimal instructions on how to reproduce key analyses (e.g., where should one go to reproduce the result in Figure 2?), and if the authors similarly packaged the environment needed to reproduce R-based analyses.

Reply: Thank you. We are glad you found the repository well-organized. We have now added a comprehensive README.md file detailing how to reproduce the results reported in the manuscript. We have tested installation and code execution on macOS and Linux operating systems. Please note that, as specified in the README.md file, the expected Python version is 3.9.

Reviewer 2

Reviewer Point P 2.1 — This is a well-crafted manuscript investigating social responses to collective adaptation and complex social learning through a social virtual 3D immersive-reality experiment involving 621 participants seeking resources under different levels of participation (solitary, groups), movement speed, and access to reward information (no payoff/NP, voluntary payoff/VP, and full payoff/FP). The authors' computational, technical, and visualization skills are evident throughout the manuscript, making it highly engaging for the reader. The careful work undertaken in developing and conducting the experiment, visualizing the results, producing video material, and presenting diagrams captures the reader's attention and sets high expectations regarding the research content and the anticipated impact of the findings. Having developed such high expectations, I note that the main limitation of the manuscript concerns its currently limited interdisciplinary positioning in terms of how the information is communicated and structured.

Reply: We thank the reviewer for the overall positive evaluation of our work and for considering the manuscript an engaging read. We have reworked both the introduction and discussion section to improve the overall structure and communication of our core contributions and to better connect our approach to related disciplines, such as economics and network science.

As a reader and reviewer with a different background from the fields the manuscript addresses, I am left wondering whether the manuscript's contribution statement (Contrasting earlier work, participants in groups outperformed solitary individuals, but only when payoff information was available") represents an advance of significant importance. I also question the realism claimed by the manuscript in the simulation, considering that the variables examined in the experiment (position: continuous variable, speed: binary variable, aggregation/collectivity level: categorical variable, payoff state: categorical variable) could be counted almost on one hand. In particular, the visibility criterion used creates a setting that appears to contradict the realism claim, as it is based on a fundamental algebraic formulation in a two-dimensional space, without a clear indication of whether proximity/apparent size is considered (see comment below).

Reply: We agree that our core advances were not communicated clearly enough in the first version of the manuscript. As now stated explicitly both in the introduction (L. 119) and the discussion (L. 532), beyond the technical innovations (which we consider important as well), our key contribution lies in mechanistically linking individual cognition, collective behavior and environmental variables in freely-interacting collectives, explaining the emergence of adaptive or maladaptive collective outcomes (L. 119).

We note that our claims to increased realism do not primarily pertain to the simulation as suggested by the reviewer but to our experimental paradigm. As also explained in response to point 1.2 by reviewer 1, we agree that "naturalistic" paradigms shouldn't be pursued for their own sake unless added complexity yields theoretical insights. By "naturalistic", we thus do not mean scenarios that are simply more complex or that appear prima facie more similar to the real world. Instead, we refer to heterogeneous and dynamically changing environments characterized by temporal and spatial autocorrelation as well as perceptual and spatial constraints (Beauchamp, 2008; Rahmani et al., 2020); in such environments, the cues participants encounter and base their decisions on are not directly imposed by the experimenter but are shaped by one's own previous actions as well as the actions of others (Brunswik, 1955; Fawcett et al., 2014). Because it has been argued that the ecological validity of such personal and social cues (i.e., the degree to which they reflect statistical patterns in the world) also determines the ecological validity of the experiment itself (Kihlstrom, 2021), naturalistic approaches in this stricter sense also help us to close the gap between experimental work and real-world behavior. Following from this understanding of "naturalism", we want to emphasize that our goal was not to include as much real-world complexity as possible (our task was still highly stylized in many ways), but to include key features of real-world decision environments that fundamentally shape the costs and benefits of social information use, but have been largely omitted in previous paradigms. These features include (1) perceptual and spatial constraints, (2) temporal and spatial autocorrelation, and (3) endogenous choice ecologies. We agree that our previous contrast between "simplified" and "naturalistic" experiments was too stark. We have now substantially re-written the respective sections in the manuscript (LL. 51–58), emphasizing how our task differs from previous approaches and why this might be more reflective of decision-making outside of the lab and, hence, more "naturalistic".

We respond to the comment on the visibility criterion below (Reviewer point 2.10).

Moreover, as a reader of a high-impact interdisciplinary journal, I am cautious when reading that the manuscript's realism is highlighted by comparison with "Previous theoretical work and experiments with fish". I share these reflections because the statement "We therefore predicted that participants in the Voluntary Payoff-Sharing condition would pay a small cost to share payoff information, and that their performance would fall in between the performance of participants in the No Payoff-Sharing and Full Payoff-Sharing conditions", as well as the manuscript's title,

resonates strongly with frameworks I am familiar from economics (e.g., monopoly: incomplete-individualized information vs. perfect competition: full information), economic geography (e.g., Allan Pred's model, see comment below), and network science (see comment on evolutionary game-theoretic information in Easley and Kleinberg, below), which makes me wonder how the manuscript sufficiently advances contemporary research findings.

Reply: We note that the previous experimental work on fish was not used as an argument to highlight the realism of our work, but to introduce the theoretical rationale of our study based on collective sensing. A guiding principle—backed by ample empirical evidence—from the fields of complex systems and collective behavior is that often very similar dynamics are at play across different social systems. These similarities allow researchers to adopt tools and results from seemingly unrelated study systems to gain deeper insights into the general forces shaping complex social systems (Mitchell, 2009; Galesic et al., 2023). In our case, different social organisms, from fish to humans, need to solve very similar challenges adapting to dynamic resource landscapes; they of course do so using very different cognitive architectures, allowing researchers to study how individual cognition interacts with environmental demands in producing adaptive or maladaptive collective outcomes across species.

We admit that we were not familiar with these studies from economics before and thank the reviewer very much for pointing us to this important line of research. We have now included a link to the economics literature in the discussion (L. 538); see more detailed response on this point below.

In this context, and to the extent that I represent a non-specialist readership in the manuscript's topic (whose engagement could multiply the article's potential impact) I encourage the authors to consider the possibility of revisiting the manuscript's motivation, contribution, and conclusions to better align with the broadened multidisciplinary scope implied by the manuscript's title and to appeal more effectively to a wider readership of the journal. For instance, while I read with particular interest the observations: "Participants who collected more points paid little visual attention to others (low out-degree), but received a lot of visual attention from others (high in-degree)..." (l.157–161) and "Discoverers who shared their payoffs received more visual attention and others increased their outward attention" (l.157–161), I notice that these are not explicitly stated as conclusions. Observations regarding participants' strategy-shifting conditions may warrant additional emphasis, and I encourage the authors to focus on them.

Reply: We are glad you found these observations interesting. Due to the richness of the paradigm and the great number of analyses we conducted, we had to be selective in which results to highlight in the discussion. The observation: "Participants who collected more points paid little visual attention to others (low out-degree), but received a lot of visual attention from others (high in-degree)..." has already been reported in a previous immersive-foraging experiment (Wu et al., 2025), which is why we only mentioned it relatively briefly. Concerning participants' strategy-shifting conditions: we have now included a new analysis comparing how participants' behavioral strategies differed between the first and second halves of the experiment, revealing both stability and temporal adaptation (see new Supplementary Section S8, Figs S23- S24, Tables S13- S14). This analysis revealed three main findings. First, baseline rotation magnitudes decreased slightly overall between the first and second halves, which might indicate increasing proficiency (avoiding unnecessary movements) or slight fatigue. Second, during *search*, we observed distinct learning dynamics: participants in group conditions seemed to evolve their reliance on social features, becoming more selectively sensitive to the position and movement of successful peers over time. These observations align with the social feature usage strategies described in the Results section. Third, and perhaps most importantly, decision weights during *tracking* remained

overall stable. While there was a minor, systematic decrease in sensitivity to private detector changes (likely due to fatigue from constant monitoring), social tracking strategies did not shift noticeably.

Reviewer Point P 2.2 — The manuscript could benefit from a typical structure (introduction, literature review, methods and data, results and discussion, conclusion). For instance, the first paragraph stands for an introduction/compact literature review on concepts, the second paragraph for an introduction/compact literature review on methods/techniques, the third paragraph for a methods section, etc.

Reply: We agree that the more traditional structure could also work well for our manuscript. However, we have decided to stick to the typical "methods-in-the-back"-style of Nature Communications. This is also meant to make the paper more accessible to a general and less technical audience that might not be interested in minute computational details.

Reviewer Point P 2.3 — The first two paragraphs (l.1-24) in the introduction would benefit from a real-world paradigm of how individuals "arbitrate between dynamically changing social and non-social features" (l.13-14) and what, e.g., "social features" (l.14) may be.

Reply: This is a great idea. We have now included social foraging (e.g., collective fishing or mushroom picking) as a real-world paradigm, also listing several examples of relevant non-social and social features in this context (L. 19).

Reviewer Point P 2.4 — In l.25, it is not clear whether the participants are human entities or are simulated agents. A multidisciplinary/non-specialized reader should seek this information within the manuscript (l.60, l.469) and supplementary information, a fact that can harm the manuscript's readability. The manuscript would benefit from such a clarification/explanation to acclimatize the unfamiliar reader

Reply: We now better clarify that our experiment is studying "groups of human participants" (L. 60), which we augmented by agent-based simulations.

Reviewer Point P 2.5 — The reward/payoff model used for the simulation (l.51) appears to be centrality-based ("the closer participants were to its (the resource's) center, the more points they collected"). I believe that the manuscript should benefit from a methodological explanation about the choice of this barycentric/core model, instead of, e.g., a payoff model proportional to the resource area scanned by the avatar.

Reply: Yes, this is correct! Participants collected points when they were positioned on the resource and collected more points if they were closer to the resource center. Such distance-dependent payoffs reflect the fundamental properties of foraging scenarios where the exploitation potential of a patchy resource (e.g., a school of fish, bush of berries, or field of strawberries) depends on the distance to the center of the patch. We have now clarified this point (L. 611).

Reviewer Point P 2.6 — Stating that "we predicted that search and tracking would be more efficient for collectives than for solitary individuals" (l.71) may not, on its own, constitute a novel research contribution of this manuscript. The benefits of collectivity, agglomeration, or clustering

are well-documented across multiple disciplines under concepts such as "economies of agglomeration", "economies of concentration", or "network effects". In this context, the observed ordering NP < VP < FP, highlighted as a major finding of the manuscript, might appear somewhat expected within a broader theoretical framework spanning various fields. For example, elements of the trade-offs among (i) the amount of available information, (ii) the skills required to process that information, and (iii) the optimal location (payoff) can be traced in spatial economics theories, including classic behavioral and location models such as Allan Pred's (1970s) Behavior and Location: Foundations for a Geographic and Dynamic Location Theory. Similarly, models of spatial clustering, as a trade-off between transportation costs and gains in firms' information availability, draw on economic geography frameworks dating back to Alfred Weber's Theory of the Location of Industries (1929, University of Chicago Press). Furthermore, Easley and Kleinberg (2010) in Networks, Crowds, and Markets: Reasoning About a Highly Connected World (Vol. 1, Cambridge University Press) provide evolutionary game-theoretic insights regarding collective dynamics, associated trade-offs, and payoffs from the perspectives of network science and network economics. In general, enough models across disciplines have examined agglomeration and network effects. Accordingly, while the NP < VP < FP finding (l.81–82) is interesting, it may not, by itself, constitute a fully sufficient contribution without further contextualization relative to the existing theoretical framework.

Reply: We thank the reviewer for directing us to this exciting body of literature from spatial economics and network science. While the theoretical benefits of collectivity, agglomeration, and network effects are well-documented across these disciplines, demonstrating that interacting human groups actually achieve these benefits in a naturalistic, dynamically changing resource landscape is not a trivial empirical finding. In fact, previous works utilizing similar, more naturalistic experimental paradigms did not show this difference at the level of overall performance (Deffner et al., 2024; Wu et al., 2025). By evaluating these dynamics within a dynamic resource landscape — an aspect that remains largely under-explored in the literature — our study provides clear advances beyond simply establishing basic collective benefits. Furthermore, our results show that simply putting individuals together does not automatically improve—and can even worsen—performance compared to singletons. Groups only outperformed solitary individuals when they selectively attended to and responded to certain social cues based on observed payoffs, and they could only do so in certain environments. Demonstrating this interplay between individual cognition, collective behavior and environmental variables, which generates (or prevents) collective intelligence, is arguably the key contribution of this work. We now clearly state this as our main theoretical contribution both in the introduction (L. 119) and the discussion (L. 428, L. 532).

We agree that there are several fascinating parallels, including the emphasis on dynamic interactions and network approaches, which we now highlight in the discussion (L. 538). However, we also want to note that the dynamics studied in our manuscript occur on a vastly different temporal and spatial scale compared to the phenomena described by Pred (1970) and Easley & Kleinberg (2010). While these research traditions study long-term and large-scale economic processes involving decisions by companies and economic institutions, we describe the fine-grained decisions processes underlying collective intelligence in dynamically interacting groups with one-second resolution. We thus want to be careful with suggesting that both observations are governed by similar decision making processes.

Reviewer Point P 2.7 — The statement "simulation work has shown that unconditional sharing of payoff information can evolve in collective search, acting as a recruitment signal that facilitates the self-organization of groups (Tump et al., 2019)" would benefit from an alternative phrasing

denoting that the findings verify the Tump et al. (2019) approach instead of showing that ...

Reply: We are not sure we fully understand this concern. The simulation work we are referencing here to explain the predictions for the voluntary payoff-sharing condition is by Tump et al. (2019). In the discussion, we state that our experimental findings from the voluntary payoff-sharing condition indeed support those theoretical predictions. Still we have now changed the respective statement in the introduction to clarify the wording (L. 112).

Reviewer Point P 2.8 — It is not clear under which criteria groups of five (l.466) were formed to proxy social collectivity. Under what theoretical/literature context/assumption was this adopted? Please support with references.

Reply: We thank the reviewer for prompting us to clarify our rationale for choosing a group size of five. While not derived from a single theoretical model, $N = 5$ was a deliberate design choice optimized for this paradigm. Group sizes of 4-6 have been routinely used in interactive group experiments (e.g., Wu et al., 2025; Deffner et al., 2024; McElreath et al., 2008, 2005), as they allow complex collective dynamics to emerge while posing manageable demands on participant recruitment and data collection. Importantly, the empirical distribution of visibility of others suggests that $N = 5$ provides a reasonably balanced rate of social encounters (see the newly added Table S12). Participants saw no other players around 43% of the time and one to two players around 42% of the time. Smaller groups would have limited the amount of social information available to participants, while larger ones might risk saturating their visual field. Additionally, from a technical standpoint, supporting larger interacting groups in a continuous, real-time 3D environment significantly increases the synchronization pressure on the network, leading to higher latency and an increased risk of participants disconnecting and being lost from the session. Making $N = 5$ a potential "sweet spot" for balancing social and private exploration, creating a dynamic that we believe is particularly relevant to the goals of our experiment. We now provide clear justifications for this design choice in the main text (Methods, L. 573).

While it would have been very interesting to also systematically compare groups of different sizes, we decided to not manipulate group size, which would have resulted in an even larger number of experimental conditions (we already had 8 between-subjects conditions). However, in the discussion, we suggest that manipulating group size would be a very interesting direction for future research and outline expectations from the theoretical and empirical literature (L. 475, L. 506).

Reviewer Point P 2.9 — Using the term 'visibility network' (l.506) in a manuscript claiming a multidisciplinary space of publication should be done after defining it. Beyond visibility networks in social sciences, the term is also widely used in time series graph analysis, as introduced by Lacasa et al. (2008) (https://www.pnas.org/doi/abs/10.1073/pnas.0709247105). Using the term without a proper conceptualization suffers from disciplinary constraints.

Reply: We thank the reviewer for highlighting the multidisciplinary usage of the term "visibility network" and agree that providing a clear definition is critical to avoid disciplinary confusion. By "visibility network," we strictly refer to physically embedded spatial interaction networks representing who can physically see whom at a given point in time, actively accounting for participant's field of view constraints. We are referring to the tradition of analyzing and modeling animal social networks (Strandburg-Peshkin et al., 2013; Poel et al., 2021). We now explicitly define this in the text (L. 127, L. 199) and provide necessary citations.

Reviewer Point P 2.10 — The visibility rule described in l.511–512 appears somewhat based on intuition and may benefit from further justification. First, the forward-facing field of view (FOV) criterion should be supported with appropriate references. Second, the visibility criterion in a 2D space is typically defined as $|\theta_i \rightarrow j - \theta_i| < FOV/2$. In the manuscript, the choice of FOV = 90 degrees (rather than, for example, 120 degrees; see e.g., [link]) is neither fully justified nor explained in context, nor is it supported by citations. In this regard, it would be helpful to clarify how sensitive the results are to the choice of FOV = 90 degrees versus 120 degrees.

Reply: Horizontal FOVs of 90-100 degrees are typical of video games for a PC where players sit relatively close to the screen (e.g., Unity Technologies, 2021; Lin et al., 2002). Moreover, we chose a relatively narrow FOV because we were interested in how participants searched for social information in the environment. As shown in the newly added Table S12, this restricted FOV meant that participants saw no peers around 43% of the time, and one or two peers around 42% of the time (now also mentioned in the main text: L. 201). This forced them to actively rotate in order to gather social information, rather than having constant peripheral awareness (which a 120-degree FOV would provide). Furthermore, as we did not record eye movements, narrow FOVs allowed us to more reliably infer social search processes from observed rotations. We now provide clear justifications for this design choice in the main text (LL. 599–603).

Reviewer Point P 2.11 — Finally, since the experiment is conducted in a virtual space, not accounting for the apparent size of objects (i.e., based on their distance from the observer) may limit the completeness of the visibility network modeling. It could be useful to consider a second visibility criterion, such as $|\theta_i \rightarrow j - \theta_i| < FOV/2$ and distance$(I, j) \leq D_{\max}$, to more fully capture participants' perceptual constraints in the simulated environment.

Reply: We thank the reviewer for this important point and for highlighting the potential role of apparent object size in our 3D virtual environment. Indeed, in many theoretical models and simulations of collective dynamics, the influence of social information on decision making is modeled as proportional to the width of the visual projection field (e.g., Bastien & Romanczuk, 2020; Mezey et al., 2024). However, we believe that implementing a hard vision range cut-off is unjustified in our current paradigm for several reasons. First, unlike physical environments, our virtual 3D environment was explicitly designed to maximize the saliency of social cues. The crater environment (400m diameter) lacks fog, atmospheric perspective, or physical obstacles. Furthermore, the avatars, and particularly the bright, towering payoff beams in the FP and VP conditions, strongly contrasted with the barren landscape. Second, when looking at the distance to an observed peer during search (participants are not on the resource themselves), there is a vanishingly small percentage of data points where peers are further than 150m ($\approx 6\%$) or 200m ($\approx 1.5\%$). Third, if introduced, the cut-off for vision range should differ between the FP, VP, and NP conditions. Finally, while systematically testing for the impact of social information salience is a fascinating direction for follow-up research – particularly integrating VR and eye-tracking to fully capture gaze movements – it falls outside the scope of the current manuscript.

Still, compelled by this line of inquiry, we tested whether participants' movement decisions were affected less by more distant peers when searching for the resource. To ensure the methodologically clearest case and avoid the complexities of aggregating multiple peers, we restricted this analysis to instances where participants observed exactly one peer. We introduced peer distance as a binary factor by splitting the observed distances at the median. The results of this model demonstrate that participants in the FP condition appear to be even more impacted by distant peers than by close ones (see Supplementary Section S9 and Fig. S27). Furthermore, despite smaller effect sizes, the decisions of

participants in the NP and VP conditions were also influenced by distant peers. Overall, we believe this analysis presents evidence *against* introducing a hard distance cut-off and indicates that physical distance does not necessarily devalue social cues in our task. We have added clarifications to the main text (L. 365, L. 448, L. 481, L. 651) and included this new analysis in Supplementary Section S9.

Reviewer Point P 2.12 — In general, all equations in the manuscript lack citations. This omission gives the impression that they are novel equations introduced in this paper, an assumption that necessitates providing their proofs in the supplementary information file. Conversely, they need to be supported by references.

Reply: We have now added citations for the key equations (see main text: L. 586, L. 655, L. 689, L. 780, L. 804; see supplement: L 22).

Reviewer 3

Reviewer Point P 3.1 — The paper presents a collective search experiment that finds that people are able to flexibly integrate individual and social sources of information. Behavioral results are accompanied by computational modeling of the obtained results and an agent-based model to test boundary conditions. It's a well-designed study, which is interesting both for the obtained results and a novel experimental paradigm. My concerns are mostly about how information is presented, and I will go more or less in order.

Reply: We are glad the reviewer found our study well-designed and considers the obtained results interesting.

Reviewer Point P 3.2 — Hypothesis 1 is well motivated by prior literature. Hypothesis 2, however, creates some tension with the Hawkins et al. (2023) study mentioned in the introduction, which found that social information was sufficient for collective search. HP 2 states that having payoff information will lead to better performance, but it does not clearly explain why. Is it because this study has a more realistic setting compared to the Hawkins et al. setup?

Reply: We thank the reviewer for this opportunity to clarify. Yes, in Hawkins et al. (2023), it was almost trivially easy for participants to identify successful participants, as they could see when someone stopped moving from a birds-eye view. In our more dynamic 3D environment, inferring success from locations and movement alone was far more challenging, which is why we predicted (and found) that social information alone would not suffice in such scenarios and explicit payoff information would be necessary for full collective benefits to show. Moreover, our task also included the simultaneous integration of personal and social features, which also made social inference harder. We believe this reasoning is explained clearly in the introduction (L. 77, L. 110)

Reviewer Point P 3.3 — Hypothesis 3 feels exploratory rather than theory-driven and, in fact, doesn't appear in preregistration. While the authors cite Tump et al. (2019) showing that payoff sharing can evolve, it doesn't explain why we should expect this to happen in a 15-minute one-shot experiment with individual payoffs and information sharing costs. The authors should either: (a)

justify the introduction of the voluntary sharing condition and their predictions in the specific paradigm used, or (b) frame this condition as exploratory but still explain the possible mechanisms of sharing in this task.

Reply: We thank the reviewer for encouraging us to clarify the theoretical basis of the Voluntary Payoff-Sharing (VP) condition. We agree that the VP condition is exploratory in nature; we have updated the manuscript accordingly (L. 116) and outlined the mechanisms that might drive sharing in this task (L. 116, L. 506).

Regarding the concern about why we might expect sharing to occur despite the costs in a short, 15-minute experiment: we set the signaling cost low enough that the average performance benefits of sharing far outweigh the costs. As detailed in our response to Reviewer 1 (Point 1.4), the maximum possible cost a participant could incur for signaling during the entire experiment is 900 points. In contrast, the performance advantage of full payoff sharing (FP) over no payoff sharing (NP) is approximately $2,500$ and $3,900$ points for the fast and slow conditions, respectively. To explicitly demonstrate that this cost was not prohibitively high for participants, we added a new supplementary analysis (Table S3), which recalculates performance by adding the signaling costs back to the participants' scores (gross score).

Indeed, after developing and piloting the study, it became clear that participants were generally quite willing to incur this small cost—with some even noting in post-experiment feedback that they actively enjoyed the interactive signaling mechanism. Our behavioral data support this observation, showing that only about 7% of participants refrained entirely from sharing payoff information (detailed in SI Section S5). Consequently, our main exploratory question was not whether people would signal at all, but rather whether the total volume of voluntary signaling during the session would be sufficient to elevate the VP condition's performance beyond the performance of the NP condition.

Reviewer Point P 3.4 — "Visibility networks" are first mentioned in line 89, but this term is not explained. Please provide an intuitive explanation when first introducing technical concepts.

Reply: We apologize for not defining this important term more clearly, which has also been mentioned by reviewer 2 (response to P 2.9). By "visibility network", we simply mean who was seeing whom at a given point in time, as we now explain in the text (L. 127, L. 199).

Reviewer Point P 3.5 — The fact that groups were a size of 5 only appears implicitly in Figure 1 and the Methods. It would help to mention this more explicitly early on, for example, around lines 54-60.

Reply: We thank the reviewer for pointing out this omission. We now clearly state in the introduction that we always recruited groups of 5 individuals (L. 93).

Reviewer Point P 3.6 — Social features are defined based on self vs the average of others (position, movement direction). However, averaging removes information about social evidence strength. For example, I would imagine people are more likely to follow a cluster of 4 others moving in the same direction than a group of 2, but averaging their positions/direction would not capture this difference. The authors should either: (a) justify why averaging is appropriate, or (b) include additional features that capture the number of visible others, their coherence, etc.

Reply: We agree with the reviewer that averaging spatial features obscures the potential strength of the social evidence, such as the difference between following a large, coherent cluster versus a single

individual. We used averaged features because, based on the empirical distribution of visibility events, instances involving large clusters within a participant's limited FOV were relatively infrequent. As shown in the newly added Table S12, participants observed one other group member for around 26% of the total time and two other members for around 16%. Observations of groups of three or four individuals were comparatively rare, happening only 10% and 5% of the time, respectively. As the majority of social observations involved only one or two peers, we concluded that an averaged metric would accurately reflect the core social dynamics while ensuring the model remained parsimonious.

Nevertheless, to fully address the reviewer's really interesting point, we have now explicitly tested whether higher-order structural features of the crowd modulate social information use in those instances where multiple peers are visible. We followed the reviewer's suggestion and fitted an extended version of our computational model exclusively to observations where participants had two or more peers in their FOV ($\approx 31\%$ of the data). We added two new features to capture the structure of the observed group: Movement Cohesion (the directional alignment among observed peers, quantified by the magnitude of the average social movement vector) and Out-degree (the number of visible peers). This new analysis (presented in Supplementary Section S9, Figures S25 and S26) revealed a highly adaptive, context-dependent shift in social information use that aligns with the reviewer's intuition. We found that when observed peers moved cohesively, participants down-weighted the group's static average position and instead aligned more with the group's movement direction. However, we did not observe consistent impact of the raw number of observed peers (out-degree), suggesting behavioral coherence is a stronger cue than the size of the observed group.

We have now added an Supplementary Section S9 describing this new model and updated the main text to both justify our use of averaging (Methods L. 724) and highlight these new findings (Results L. 362; Discussion L. 445).

Reviewer Point P 3.7 — Figure 4: I believe the legend should say NP, VP, FP, and not NS, VS, AS?

Reply: Fixed.

Reviewer Point P 3.8 — There appears to be considerable variability in individual performance in all conditions (figures S4, S5), and the main text mentions that people vary in their reliance on different cues, but these individual differences aren't analyzed in detail. I understand it's difficult to fit everything into 1 paper, but it would be nice to see a more focused paragraph on this issue, or an additional figure (e.g., computational model weights across individuals) in the main text or the Supplement.

Reply: We agree that a more in-depth analysis of the causes and consequences of individual variability would be very interesting but would warrant its own manuscript. In this manuscript, we did indeed analyze how participants' reliance on different (social and non-social) cues was associated with their performance in terms of tracking efficiency (Fig. S21) and tracking time (Fig. S22). As explained in the main text, these analyses showed, for example, that higher detector sensitivity was generally associated with success, that movement direction was a more reliable cue than location, and, most importantly, that payoff selectivity with respect to both position and movement direction of others was predictive of tracking efficiency.

To visualize the full individual heterogeneity in decision strategies, we have now included additional plots showing individual random effects for the feature weights of the computational models (See Figs. S19, S20).

Reviewer Point P 3.9 — Similarly, the paper doesn't examine temporal dynamics within the 15-minute session. Is there any evidence of improvement over the session? Either individually or with respect to group strategies? The authors could divide the data into epochs, or at least examine the beginning and the end of the session. This could tell us whether conditions differ because there are stable differences in people's strategies depending on available cues, or if they differ due to distinct effects of cue availability on collective learning. The changes over time are particularly important for the voluntary sharing condition.

Reply: We thank the reviewer for this excellent suggestion. We agree that analyzing these temporal dynamics is key to understanding if participants use stable strategies or adapt over time. This is indeed particularly interesting for the Voluntary Payoff-Sharing (VP) condition.

As briefly noted in our supplementary materials (Fig. S8C) and discussed in Section S5, there are already indications that payoff sharing behavior evolves: over time, sharing became more common in the fast-resource condition when participants were tracking, whereas participants shared less often over time when searching in the slow-resource environment. While this provides an exciting initial indication of collective learning, analyzing temporal dynamics in our continuous, unconstrained task presents unique challenges. Because we cannot control the prevalence and order of tracking versus searching states, different participants experience vastly different state exposures (e.g., one participant might spend the first half of the experiment searching, while another primarily tracks). Given these individual variations and the relatively short 15-minute timeframe, we initially hesitated to place strong emphasis on the learning dynamics.

Nevertheless, we agree that it is important to check whether there has been a systematic shift in performance throughout the study. So we first tested for the high-level change in the individual performance, via comparing collected points for participants in the first and the second halves of the experiment. We did not find evidence for a change in performance in any of the conditions (see Fig. S4 and Table S4). Next we looked into the group-level performance, analyzing the change in group median distance to the resource center. Again, we did not find evidence to support a change in performance (see Fig. S30 and Table S16). While the overall performance seems to be stable over the course of the experiment, behavioral strategies might still evolve even if this did not lead to a noticeable performance change. Therefore, we have now introduced a temporal feature in the rotation models to control for these dynamics, dividing the data into two epochs: the first half (0–7.5 min) versus the second half (7.5–15 min) of the experiment. We integrated this feature into our computational models by including interaction terms between the epoch (early vs. late) and the participants' private and social feature weights (see new Supplementary Section S8, Figs S23- S24, Tables S13- S14). Our new temporal analysis revealed three main findings. First, baseline rotation magnitudes decreased slightly overall between the first and second halves, which might indicate increasing proficiency (avoiding unnecessary movements) or slight fatigue. Second, during *search*, we observed distinct learning dynamics: participants in group conditions seemed to evolve their reliance on social features, becoming more selectively sensitive to the position and movement of successful peers over time. These observations align with the social feature usage strategies described in the Results section. Third, and perhaps most importantly, decision weights during *tracking* remained overall stable. While there was a minor, systematic decrease in sensitivity to private detector changes (likely due to fatigue from constant monitoring), social tracking strategies did not shift noticeably.

We have added these new temporal analyses to the Supplementary Information and updated the main manuscript to discuss these dynamics (see Results, L. 179, L. 348, and Discussion, L. 503).

Reviewer Point P 3.10 — The paper does many things - introduce a more complex task (continuous tracking in 3D), a notion of payoff selectivity, a result that NP groups do worse than individuals. It would help to make it clearer (explicitly stated) what the authors consider to be their main contribution/novelty.

Reply: We agree that our core advances were not communicated clearly enough in the first version of the manuscript. As now stated explicitly both in the introduction (L. 119) and the discussion (L. 532), beyond the technical innovations (which we consider important as well), our key contribution lies in mechanistically linking individual cognition, collective behavior and environmental variables in freely-interacting collectives, explaining the emergence of adaptive or maladaptive collective outcomes (L. 119).

Reviewer Point P 3.11 — I have concerns about framing the task that relies solely on individual payoffs as 'collective intelligence' (including in the title). Much of the analysis focuses on how individuals use various cues to improve their performance, and so it is definitely a paper about how individuals can do better (but sometimes worse) in group contexts. Collective intelligence would require that there are emergent group-level processes or outcomes that are not reducible to individuals (e.g., Woolley et al., 2010). The authors suggest that, for example, groups can stay on target longer because of how they learn from and follow each other (as a kind of social amplification of search patterns), but this is not highlighted or quantified as an emergent collective effect, distinct from individuals just benefiting from information collected by others. I recommend either (a) reframing the paper as being about 'adaptive social learning in groups' or (b) adding analyses that genuinely demonstrate collective-level phenomena.

Reply: We thank the reviewer for bringing up these important conceptual points. In our analysis, we focused on comparing average performance between groups and solitary individuals, aiming primarily to disentangle the concrete mechanisms that distinguish groups showing collective intelligence (CI; below we discuss different definitions of the CI concept in the literature) from those that do not. We took *collective sensing* (Berdahl et al., 2013; Hein et al., 2015; Puckett et al., 2018; Hawkins et al., 2023)—an unambiguous example of CI—as our theoretical starting point, which by design most closely corresponds to our FP condition. The FP condition serves as an "upper bound" for CI in our paradigm, as participants could immediately recognize whether a peer was in a more favorable location and adapt their behavior accordingly. Analogous to collective sensing research, our analysis confirms that participants in FP groups achieve superior performance while relying less on their private detectors (see Fig. 4). Crucially, however, we went further by demonstrating the boundary conditions under which CI fails to emerge, our NP condition. It is also important to note that, in our view, CI is not a binary trait, but rather something that can be manifested to varying degrees in different groups.

Moreover, we would argue that, in a sense, all our analyses focus on genuinely collective-level phenomena. Unlike more constrained experiments, where participants respond to static social environments, in our study, individuals always respond to social environments created by previous choices of others, generating rich dynamical feedback loops between individual choices and collective phenomena. We consider individual cue integration and choices as the basis of collective behavior, with the agent-based simulations explicitly linking the results obtained about individual decisions to group outcomes. Furthermore, the dynamic visibility network analysis in particular (Fig. 3) focuses on inherently collective adaptation processes, strongly suggesting that groups (especially in the FP condition) adapted to the environment *as a whole*.

In general, we believe there are multiple—only partly overlapping—definitions of CI across different disciplines (Galesic et al., 2023). While the definition by Woolley et al. (2010) ("the general ability of the group to perform a wide variety of tasks") focuses on the transferability of group performance across domains independently of individual ability; other prominent definitions focus on the comparison between individuals acting alone with individuals acting in groups, with CI being characterized by participants in groups outperforming singletons (e.g., "the ability of individuals to collectively act in ways that seem intelligent, often displaying intelligence surpassing that of individuals acting alone on tasks such as idea generation, problem-solving, estimation, inference and decision-making" (Burton et al., 2024; Malone & Bernstein, 2015)). The latter definition is also in line with views from computer science and biology where CI is used to describe novel collective-level adaptive dynamics emerging from local, individual-level cue integration (Galesic et al., 2023). Lastly, there is also the especially strict definition of CI as only referring to group-living animals "solving cognitive problems that go beyond the capacity of single animals" (Krause et al., 2010). While both our experimental and simulation work demonstrate CI in the sense of groups outperforming individuals *on average* (in line with the definitions of Burton et al. (2024)), the experiments arguably did not provide evidence for CI exceeding the capacity of the most-skilled individuals (as demanded by Krause et al. (2010); see Fig. S3). Still, our simulation work reveals CI even in this strongest sense with (selective) social agents outperforming the best individuals. We have now added a discussion of the different uses of the term in the literature and explained which kind(s) of CI are supported by our findings (LL. 509–527).

Lastly, to explicitly quantify the emergent nature of collective intelligence in our paradigm, we have added a new analysis comparing the interacting groups against synthetic "nominal groups" – constructed by randomly combining the time-series data of five solitary participants (see Fig. S29, Table S15). While nominal groups mathematically benefit from maximum spatial dispersion (where one individual might randomly come close to the resource), they lack the mechanisms to pull the rest of the group to optimal areas in the environment. To isolate the synergistic benefits of social interaction, we measured the *median* distance of the group to the resource center at each time step. This specifically captures the ability of the group to cohesively coalesce the *majority* of their members around the resource. As expected, this group-level analysis clearly indicates that FP groups are substantially closer to the resource center than the average nominal group ($20.77[9.91, 31.46]$m closer in fast environments, and $13.58[9.69, 17.35]$m closer in slow environments). Therefore, by allowing participants to observe each other, we created a situation where interacting groups as a whole outperformed the "nominal baseline". This, in our view, is a clear indication of collective intelligence. Unsurprisingly, this group-level performance benefit is also reflected in the individual performance scores of the group members. We have added this note to the Results section (L. 183).

Remarks on code availability

Reviewer Point P 3.12 — I opened the code link and saw the folders with files. I was able to download each file individually, but not the zip of everything ("code": 503, "message": "Unable to connect to auth sever"). There's no README, so it's daunting to try to understand what to run and in which order.

Reply: We have now added a comprehensive README.md file detailing how to reproduce the results reported in the manuscript. We have tested installation and code execution on macOS and Linux operating systems. Please note that, as specified in the README.md file, the expected Python version is 3.9.

References

Bastien, R. & Romanczuk, P. (2020). A model of collective behavior based purely on vision. *Science advances*, 6(6), eaay0792.

Beauchamp, G. (2008). A spatial model of producing and scrounging. *Animal Behaviour*, 76(6), 1935–1942.

Berdahl, A., Torney, C. J., Ioannou, C. C., Faria, J. J., & Couzin, I. D. (2013). Emergent Sensing of Complex Environments by Mobile Animal Groups. *Science*, 339(6119), 574–576.

Brunswik, E. (1955). Representative design and probabilistic theory in a functional psychology. *Psychological Review*, 62(3), 193–217.

Burton, J. W., Lopez-Lopez, E., Hechtlinger, S., Rahwan, Z., Aeschbach, S., Bakker, M. A., Becker, J. A., Berditchevskaia, A., Berger, J., Brinkmann, L., et al. (2024). How large language models can reshape collective intelligence. *Nature human behaviour*, 8(9), 1643–1655.

Deffner, D., Mezey, D., Kahl, B., Schakowski, A., Romanczuk, P., Wu, C. M., & Kurvers, R. H. (2024). Collective incentives reduce over-exploitation of social information in unconstrained human groups. *Nature Communications*, 15(1), 2683.

Fawcett, T. W., Fallenstein, B., Higginson, A. D., Houston, A. I., Mallpress, D. E., Trimmer, P. C., & McNamara, J. M. (2014). The evolution of decision rules in complex environments. *Trends in Cognitive Sciences*, 18(3), 153–161.

Galesic, M., Barkoczi, D., Berdahl, A. M., Biro, D., Carbone, G., Giannoccaro, I., Goldstone, R. L., Gonzalez, C., Kandler, A., Kao, A. B., et al. (2023). Beyond collective intelligence: Collective adaptation. *Journal of the Royal Society Interface*, 20(200), 20220736.

Hawkins, R. D., Berdahl, A. M., Pentland, A. S., Tenenbaum, J. B., Goodman, N. D., & Krafft, P. M. (2023). Flexible social inference facilitates targeted social learning when rewards are not observable. *Nature Human Behaviour*, (pp. 1–10).

Hein, A. M., Rosenthal, S. B., Hagstrom, G. I., Berdahl, A., Torney, C. J., & Couzin, I. D. (2015). The evolution of distributed sensing and collective computation in animal populations. *eLife*, 4.

Kihlstrom, J. F. (2021). Ecological validity and "ecological validity". *Perspectives on Psychological Science*, 16(2), 466–471.

Krause, J., Ruxton, G. D., & Krause, S. (2010). Swarm intelligence in animals and humans. *Trends in ecology & evolution*, 25(1), 28–34.

Lin, J. J.-W., Duh, H. B., Abi-Rached, H., Parker, D. E., & Iii, T. A. F. (2002). Effects of field of view on presence, enjoyment, memory, and simulator sickness in a virtual environment. In *Proceedings IEEE Virtual Reality 2002* (pp. 164). Los Alamitos, CA, USA: IEEE Computer Society.

Malone, T. W. & Bernstein, M. (2015). *Handbook of collective intelligence*. MIT press.

McElreath, R., Bell, A. V., Efferson, C., Lubell, M., Richerson, P. J., & Waring, T. (2008). Beyond existence and aiming outside the laboratory: estimating frequency-dependent and pay-off-biased social learning strategies. *Philosophical Transactions of the Royal Society B: Biological Sciences*, 363(1509), 3515–3528.

McElreath, R., Lubell, M., Richerson, P. J., Waring, T. M., Baum, W., Edsten, E., Efferson, C., & Paciotti, B. (2005). Applying evolutionary models to the laboratory study of social learning. *Evolution and Human Behavior*, 26(6), 483–508.

Mezey, D., Deffner, D., Kurvers, R. H., & Romanczuk, P. (2024). Visual social information use in collective foraging. *PLOS Computational Biology*, 20(5), e1012087.

Mitchell, M. (2009). *Complexity: A guided tour*. Oxford university press.

Poel, W., Winklmayr, C., & Romanczuk, P. (2021). Spatial Structure and Information Transfer in Visual Networks. *Frontiers in Physics*, 9.

Puckett, J. G., Pokhrel, A. R., & Giannini, J. A. (2018). Collective gradient sensing in fish schools. *Scientific Reports*, 8(1), 7587.

Rahmani, P., Peruani, F., & Romanczuk, P. (2020). Flocking in complex environments—attention trade-offs in collective information processing. *PLoS computational biology*, 16(4), e1007697.

Strandburg-Peshkin, A., Twomey, C. R., Bode, N. W., Kao, A. B., Katz, Y., Ioannou, C. C., Rosenthal, S. B., Torney, C. J., Wu, H. S., Levin, S. A., et al. (2013). Visual sensory networks and effective information transfer in animal groups. *Current Biology*, 23(17), R709–R711.

Unity Technologies (2021). *Unity Game Engine Documentation*. Version 2021.3.14f1, built-in rendering pipeline.

Woolley, A. W., Chabris, C. F., Pentland, A., Hashmi, N., & Malone, T. W. (2010). Evidence for a collective intelligence factor in the performance of human groups. *science*, 330(6004), 686–688.

Wu, C. M., Deffner, D., Kahl, B., Meder, B., Ho, M. H., & Kurvers, R. H. (2025). Adaptive mechanisms of social and asocial learning in immersive collective foraging. *Nature Communications*, 16(1), 3539.

Response to Reviewers: Second Review

Reviewer #1 (Remarks to the Author):

In this revised manuscript, the authors have made the following changes in response to my comments:

(1) Reworked the introduction to clarify the theoretical claims of the paper, particularly by specifying in what ways the task is "naturalistic"
(2) Reworked the introduction, discussion, and results to define the concept of "payoff selectivity" and explicitly tie it to analyses
(3) Justified the small additional cost in the VP condition and run additional analyses to check whether performance is artificially deflated in this condition because of this cost

Overall, I am impressed by the latest round of revisions and feel that all of my comments have been addressed. I particularly appreciated the new explanation of "naturalism"; I expect that I'll refer back to and cite the authors' explanation here often in future reviews.

Authors' Response

> We are pleased that the reviewer is satisfied with our latest revisions and thank them for their valuable feedback.

My one suggestion is that the discussion of payoff selectivity in LL.19-41 is a bit dense. Looking through the analyses that now explicitly refer to payoff selectivity, it seems that what the authors *actually* looked at is whether people can adaptively tune their information use based on others' payoffs, particularly in more complex environments where they don't have all the information available to them at once (compared to, say, a birds'-eye view) and where others may not always disclose their payoffs. It would help to simplify this section a bit so that the research question comes through more clearly—as a reader, my sense was that the list of payoff-based processes in LL.29-41 is a bit too complex, so it's hard to tell what's most important and relevant to the paper here. (For example, it's interesting to consider that others' payoffs may not always directly inform the best action to take—as in the case of Witt et al., 2024, where partners' preferences can differ from our own. But that doesn't seem relevant to the current paper, so it threw me for a loop.)

Authors' Response

> We agree that this section is quite dense because it references multiple processes. While we believe it is important to explicitly compare the various payoff-based processes discussed in the literature, we have followed the reviewer's suggestion and removed the discussion on interindividual reward differences, which was indeed only tangentially related to the present work.

Reviewer #2 (Remarks to the Author):

The authors have addressed my concerns with seriousness and professionalism. For example, I appreciate that each of my suggestions led to revisions or modifications in the text, resulting in improved readability and presentation. For instance, I welcome the additional discussions regarding the integration mechanisms of fish or mushrooms, the clarifications on "naturalistic" versus "real-world" or realistic scenarios, the definition of visibility networks in this research context, and the selection of forward-facing fields of view.

Authors' Response

> We are glad the reviewer considers our revisions both serious and professional, and thank them for suggesting to explicitly quantify the role of group size.

However, my remaining concern stems from comment numbered "Point P2," which pertains to group size. Although the authors have provided a convincing response regarding the specific framework of Reviewer Point P2.8, I still have reservations about the sensitivity of the overall research design on the outcomes. In particular, while I feel the design is over-parameterized, I would like to see a sensitivity assessment regarding group size and a more comprehensive discussion about the sensitivity of the research parameters overall. As a non-specialist reader, I find that articles published in NCOMMS should not be overly case-sensitive (such papers would, e.g., be better suited for SciRep) and should present generalizable outcomes.

Therefore, I kindly ask the authors to provide further evidence supporting the group size sensitivity and generality of their approach and to better highlight their contributions for a multidisciplinary audience, which is the target readership of this journal.

Authors' Response

> We are glad the reviewer considers our revisions both serious and professional, and thank them for suggesting to explicitly quantify the role of group size.
>
> In response, we have run our agent-based simulations (which had already explored the generality of our findings with respect to the environment) with different group sizes ranging from 3 to 99 (see new Fig. S31). This analysis showed that our core results remained the same irrespective of group size, but also added some interesting nuance. In particular, the dangers of maladaptive herding for non-selective agents systematically increased with group size, as uninformative social cues became more prevalent.

Reviewer #3 (Remarks to the Author):

The authors have significantly improved the manuscript. I find that most of my concerns have been addressed by either clarification or additional analysis. It was especially interesting to see new results that take into account additional social features and temporal dynamics - I think they both strengthen the current paper and open avenues for further research. I also appreciated an explicit discussion of different definitions of collective intelligence and how they relate to the paper.

Authors' Response

> We also thank reviewer #3 for their positive evaluation of our revisions. We agree that they have significantly improved the manuscript.

I have two remaining concerns, both of which have to do with the conceptual clarity of the Introduction. I would recommend revising these before accepting for publication.

1. Voluntary Payoff Sharing rationale (lines 110-116): The term "unconditional sharing" from Tump et al. means sharing freely, but as a term feels in tension with the contrast between "voluntary" and "full" (that is, intuitively I would see "full sharing" as "unconditional" as "voluntary" is conditional on the individual's choice). More importantly, the prediction that voluntary sharing would fall "between" NP and FP conditions isn't well justified. I could well imagine two other outcomes: 1) VP could fall below both NP and FP, for example, due to cost (in points) or cognitive cost (trying to decide whether to share); 2) VP could fall above FP because strategic sharing might be more informative. If the authors insist on making a specific prediction for VP, they would need to justify it better. However, I think it would be better (more consistent with the condition's exploratory nature) to frame it as an open empirical question with 3 possible outcomes.

Authors' Response

> We agree with the reviewer that the term "unconditional sharing" might be confusing in the context of our study. We therefore re-worded this to "active sharing". We also agree that it is more transparent to not list any predictions for this condition as this was also not included in the preregistration. In response, we have removed the prediction and stated that we "did not formulate any prior predictions on their resulting performance" (l.100).

2. The last paragraph of the Introduction (lines 117-137) is the most crucial in delineating the paper's contribution. It should clearly define the key concept, "payoff selectivity," as soon as it is introduced. Then, the paragraph would be better structured as a clear explanation of how individual cognition, collective behavior, and environmental variables are mechanistically linked. Currently, it reads more like a results summary (a list) than a contribution statement (clarification of how everything fits together). Consider restructuring to: (a) define payoff selectivity explicitly, (b) show the chain of logic connecting individual mechanisms -> collective patterns -> environmental contingencies (back to individual mechanisms?), and (c) clarify how this multi-level integration is your novel contribution.

Authors' Response

> We thank the reviewer for this final comment. Please note that the term "payoff selectivity" is already defined earlier in the introduction (l. 18).
> The reviewer is correct that the paragraph reads like a results summary which is also what we intended. We believe it is appropriate to end the introduction with a high-level overview guiding the reader through our complex set of analyses. A dedicated contribution statement is already given in the discussion (ll. 510-524).

Reviewer #3 (Remarks on code availability):

I installed and ran the code with small issues:
- I think it is better to specify Python version in the venv line: `python3.9 -m venv venv`
- The tables package requires HDF5 to be installed
- Add `pip install ipykernel' to the commands in step 3

The rest run successfully and replicated.

Authors' Response

Thank you. We have made those changes to our repo.
